# Supplementary material for: Efficacy and safety of temperature-sensitive acellular dermal matrix in prevention of postoperative adhesion after thyroidectomy: A randomized, multicenter, double-blind, non-inferiority study
Source: PLoS One. 2022 Sep 19;17(9):e0273215. doi: 10.1371/journal.pone.0273215 (PMC9484646; doi:10.1371/journal.pone.0273215)
Supplement: S1 Table — (DOCX) [file pone.0273215.s002.docx]

**S1 Table: Total setting of the enrolled patients**

|  | MegaShield (Test device) | | | | Guardix-SG (Control device) | | | | Total | | | |
| --- | --- | --- | --- | --- | --- | --- | --- | --- | --- | --- | --- | --- |
| Investigator site | SEV | KBSMC | CMC | Subtotal | SEV | KBSMC | CMC | Subtotal | SEV | KBSMC | CMC | Total |
| Target number of subject |  |  |  | 70 (100.0) |  |  |  | 70 (100.0) |  |  |  | 140 (100.0) |
| Analysis sets |  |  |  |  |  |  |  |  |  |  |  |  |
| ITT set | 35 | 22 | 13 | 70 (100.0) | 35 | 22 | 13 | 70 (100.0) | 70 | 44 | 26 | 140 (100.0) |
| mITT set | 35 | 19 | 8 | 62 (88.6) | 35 | 22 | 8 | 65 (92.8) | 70 | 41 | 16 | 127 (90.7) |
| PP set | 35 | 19 | 7 | 61 (87.1) | 35 | 22 | 8 | 65 (92.8) | 70 | 41 | 15 | 126 (90.0) |
| N (%); SEV: Severance hospital; KBSMC: Kangbuk samsung hospital; CMC: Catholic university Seoul st. Mary's hospital | | | | | | | | | | | | |
