# Supplementary material for: Efficacy and safety of temperature-sensitive acellular dermal matrix in prevention of postoperative adhesion after thyroidectomy: A randomized, multicenter, double-blind, non-inferiority study
Source: PLoS One. 2022 Sep 19;17(9):e0273215. doi: 10.1371/journal.pone.0273215 (PMC9484646; doi:10.1371/journal.pone.0273215)
Supplement: S1 File — (PDF) [file pone.0273215.s003.pdf]

【임상시험연구계획서】

유착방지재 메가쉴드(MegaShield)와 가딕스-에스지(Guardix-SG) 의 갑상선 전절제술 후 유착방지효과 및 안전성을 비교 평가하기 위한 다기관, 이중맹검, 비열등, 무작위배정, 전향적 확증 임상시험

임상시험계획서 번호 : LNC-MS-001

Version : 2.11

(2020. 07. 06)

㈜엘앤씨바이오

< 목 차 >

[임상시험 연구계획서 개요] 5
[임상시험 진행 일정표] 7
1. 임상시험의 제목 8
2. 임상시험기관의 명칭 및 소재지 8
3. 임상시험의 책임자 . 담당자 및 공동연구자의 성명 및 직명 8
4. 임상시험용 의료기기를 관리하는 관리자의 성명 및 직명 9
5. 임상시험을 하려는 자의 성명 및 주소 9
5.1 의뢰자 9
5.2 임상시험수탁기관 (모니터 업무) 9
6. 임상시험의 목적 및 배경 9
6.1 임상시험의 목적 9
6.2 임상시험의 배경 9
7. 임상시험용 의료기기의 개요 (사용목적, 대상질환 또는 적응증) 11
8. 임상시험 의료기기의 적용 대상이 되거나 대조군에 포함되어 임상시험에 참여하는 사람(이하 "피험자"라 한다)의 선정기준 · 제외기준·인원 및 그 근거 11
8.1 선정기준 11
8.2 제외기준 12
8.3 피험자 수의 선정 12
8.4 산출 근거 12
8.5 연구 대상자 모집 계획 13
9. 임상시험기간 13
10. 임상시험 방법 (사용량·사용방법·사용기간·병용요법 등) 14
10.1 임상시험 디자인 14
10.2 임상시험용 의료기기 (시험군) 14
10.3 대조시험용 의료기기 (대조군) 15
10.4 임상시험방법 16
10.5 병용 금기 약물 18
11. 관찰항목•임상검사항목 및 관찰검사 방법 18
11.1. 관찰항목 및 임상검사 항목 19
11.2. 관찰검사 방법 20
11.2.1 피험자 동의서 서명 21
11.2.2 인구학적 조사 및 병력 조사 21
11.2.3 피험자 적합성 평가 21
11.2.4 피험자 식별코드 부여 21
11.3 눈가림 배정 방법 21
11.3.1 무작위 배정 21
11.3.2 눈가림해제(Unblinding) 22

11.4 독립적 평가자의 평가 23
11.5 1차 유효성 평가 : Marshmallow 식도 조영술을 이용한 식도 운동능력 비교 평가 23
11.6 2차 유효성 평가 : 유착 점수 (Adhesion Scores) 23
11.7 안전성 평가 24
12. 예측되는 부작용 및 사용시 주의 사항 24
12.1 예측되는 부작용 24
12.2 시험용 의료기기 사용시 주의사항 25
12.2.1 일반적 주의사항 25
12.2.2 의료기기의 사용 결과 발생할 수 있는 이상반응 사용상의 부주의에 따른 치명적인 부작용 · 사고발생 등에 대한 주의사항 25
12.2.3 임부, 수유부, 가임여성, 신생아, 유아, 소아, 고령자에 대한 사용 26
12.2.4 적용상의 주의 26
12.2.5 안전사고의 예방에 필요한 사항 26
12.3 대조용 의료기기 사용시 주의사항 26
12.3.1 일반적인 주의사항 26
12.3.2 취급 시 주의사항 26
12.3.3 금기사항 27
12.3.4 예측 부작용 27
13. 중지·탈락 기준 27
13.1 중지 기준 27
13.2 중지의 처리 27
13.3 탈락기준 28
13.4 탈락의 처리 28
14. 유효성의 평가기준, 평가방법 및 해석방법(통계분석법에 의함) 28
14.1 1차 유효성 평가 28
14.2 2차 유효성 평가 29
14.3 통계 분석 대상 집단 30
14.4 결측치 처리 30
14.5 인구학적 및 기초자료 분석 30
15. 부작용을 포함한 안전성의 평가 기준 · 평가 방법 및 보고방법 31
15.1 이상반응의 정의 31
15.2 중대한 이상반응/의료기기이상반응(Serious AE/ADE) 정의 31
15.3 이상반응의 평가 31
15.3.1 중증도 평가 32
15.3.2 임상시험용 의료기기와의 인과관계 평가 32
15.4 안전성의 평가기준 33
15.4.1 예측되는 부작용 33
15.5 안전성 평가방법 (통계분석방법) 33
15.6 이상반응 교육 및 보고방법 34

15.6.1이상반응 교육 34
15.6.2 이상반응의 문서화 34
15.6.3 중대한 이상반응/이상의료기기반응 교육 35
15.6.4 이상반응 발생 시 조치사항 36
15.6.5 이상반응의 추적관찰 36
16. 피험자 동의서 서식 37
17. 피해자 보상에 대한 규약 45
17.1 피해자 보상 사유 45
17.2 보상요건 45
17.3 보상 제외사유 45
17.4 보상기준 45
17.5 보상절차 46
17.6 적용범위 46
18. 임상시험 후 피험자의 진료에 관한 사항 47
19. 피험자의 안전보호에 관한 대책 47
19.1 임상시험실시기관 47
19.2 임상시험심사위원회 47
19.3 시험자 47
19.4 의뢰자 48
19.5 모니터링 48
19.6 임상시험계획서의 변경 48
19.7 피험자 동의 49
19.8 피험자 기록의 비밀보장 49
19.9 기록의 보존 49
20. 그 밖의 임상시험을 안전하고 과학적으로 실시하기 위하여 필요한 사항 49
20.1 임상시험용 의료기기의 사용 및 관리 49
20.2 임상시험용 의료기기의 공급과 취급 50
21. 참고문헌 50

【임상시험 연구계획서 개요】

제목	**유착방지재 메가쉴드(MegaShield)와 가딕스-에스지(Guardix-SG)의 갑상선 전절제술 후 유착방지효과 및 안전성을 비교 평가하기 위한 다기관, 이중맹검, 비열등, 무작위배정, 전향적 확증 임상시험**
시험목적	메가쉴드(MegaShield)의 갑상선 수술 후 유착 방지 효과를 평가하기 위해, 갑상선 전절제 환자에게 메가쉴드(MegaShield) 또는 가딕스-에스지(Guardix-SG)를 적용하여 유착방지 효과 및 안전성을 비교 평가하고자 한다.
의뢰자	㈜엘앤씨바이오
시험기관	연세대학교 의과대학 세브란스 병원<br>의료법인 삼성의료재단 강북삼성병원<br>가톨릭대학교 서울성모병원
연구기간	승인일로부터 32 개월 (2018 년 4 월~2020 년 12 월)
피험자수	총 140 명 (시험군과 대조군 각각 70 명)
의료기기정보	· 시험의료기기 : 메가쉴드 (MegaShield): 무세포동종진피 분말, 가교 히알루론산, 온도감응성 고분자를 혼합하여 제조한 투명 혹은 유백색을 띠는 겔 타입의 액체<br>· 대조의료기기 : 가딕스-에스지(Guardix-SG): 플록사머와 알긴산 나트륨으로 구성된 제품.
피험자<br>선정 및 제외<br>기준	<선정기준><br>모든 피험자는 아래의 기준에 모두 부합할 때에만 임상시험에 참여할 수 있다.<br>1) 연구참여에 대한 피험자 서면동의에 자발적으로 서명, 날인한 자<br>2) 연령이 만 20세 이상, 70세 미만인 환자<br>3) 임상시험기간 동안 참여 가능한 환자<br>4) 갑상선 질환으로 갑상선 전절제술이 필요한 환자<br>5) 갑상선 질환과 관련된 갑상선 절제에 대한 최초 수술 예정인 환자<br>6) 수술 전 검사상 간 기능 이상 및 빈혈, 신장 기능 저하 등이 없는 환자<br>7) 임상시험 의료기기 적용 후 임상시험 참여 기간동안 피임에 동의한 자<br><제외기준><br>다음의 항목 중 하나라도 해당되면 피험자는 임상 시험에서 제외한다.<br>1) 임신 중이거나 수유중인 여성 혹은 임상 시험용 제품 적용 후 1개월 이내에 임신할 여성<br>2) 중대한 간장 혹은 신장 질환이 있는 자<br>3) 림프액 혹은 혈액 응고질환이 있는 환자 혹은 항혈액응고제를 투여하는 자<br>4) 당뇨용 경구적 혹은 비경구적 혈당강하제를 투여하는 자

	5) 면역 억제된 환자 혹은 자가면역질환이 있는 자<br>6) 중대한 전신성 질환을 가진 자<br>7) 동반된 후속 수술이 예정된 환자<br>8) 갑상선암 이외의 암으로 항암치료중인 환자<br>9) 다른 유착방지재를 처치하는 환자<br>10) 연구자가 부적절하다고 판단하는 자(비협조성 등 포함)
시험방법	선정제외기준에 부합하는 환자는 눈가림 배정표에 따라 배정된다. 수술 당일에 대상환자는 갑상선전절제술을 시행하고 배정받은 의료기기를 적용한다. 의료기기 적용 시, 시험군의 경우 MegaShield 5ml를 , 대조군의 경우 Guardix-SG 5ml를 갑상선 절제부위와 띠근육 표면에 도포한다. 수술 후 1주와 6주째에 방문하여 유효성 및 안전성을 평가한다.
평가방법 및 기준	**유효성 평가**<br>· 1차 유효성 평가 : 유착의 정도를 평가하기 위해 수술 6주 후 Marshmallow 식도조영술을 이용하여 두 군간 식도 운동 능력을 비교 평가한다.<br>· 2차 유효성 평가 : 두 군간 유착 점수 (Adhesion Scores)를 스크리닝 시, 수술 1주 후, 수술 6주 후 비교한다.<br>**안전성 평가**<br>모든 이상반응 : 수술후 매방문시
통계방법	주분석군은 mITT set이며, PP set을 추가로 분석한다. 안전성의 분석군은 ITT set이다.<br>· ITT Set: 본 임상시험에 등록된 모든 피험자 중 임상시험용 의료기기로 적어도 한 번 이상 치료를 받은 피험자를 대상으로 한다.<br>· Modified ITT Set: 본 임상시험에 등록된 모든 피험자 중 임상시험용 의료기기로 적어도 한 번 이상 치료를 받고 1차 유효성 평가에 참여한 피험자를 대상으로 한다<br>· PP set: ITT set 대상자 중 연구계획서의 중대한 위반 없이 본 계획서에 따라 시험 일정이 완료된 피험자를 대상으로 한다.<br>1차 유효성 통계분석방법은 식도운동능력점수의 빈도, 비율 및 평균, 표준편차, 중위수, 최소값, 최대값을 제시하고, 새로운 치료법의 효과가 기존 치료법보다는 비열등한가를 확인하기 위해 두 군 간의 '식도운동능력' 평균 점수 차이에 대한 단측 97.5% 신뢰구간을 사용하여 판단한다.<br>2차 유효성 평가 분석은 '시점별 군별 기술통계량(평균, 표준편차, 중위수, 최소값, 최대값)을 제시하고 스크리닝 대비 수술 후 시점별 군별 평균변화량의 기술통계량을 제시한다<br>안전성의 분석군은 ITT set을 대상으로 한다. 시험기간동안 보고된 모든 부작용은 군별 발생명수, 비율 및 건수를 구한다. 시험군과 대조군 간 이상반응 발생율을 chi-square test (Fisher's exact test로 분석한다.

【임상시험 진행 일정표】

수행항목	스크리닝	적용 및 평가		
Visit	Visit 1	Visit 2	Visit 3	Visit 4
방문일(Day)	-30~0	Day 0	Day 7±3	Day 42±7
서면동의서 수령	●			
선정 제외 기준 확인	●			
인구학적조사/신체검사	●			
신체활력증후 검사	●			●
병력/투약력(1)	●			
피험자 등록		●		
수술 및 임상용 의료기기 적용		●		
식도조영술				●
유착점수 평가	●		●	●
소변 β-HCG 검사 (가임여성)	●			●
혈액학적검사	●		●	●
혈청생화학검사	●		●	●
소변 검사	●		●	●
병용약물수집(2)	●	●	●	●
이상반응수집(3)		●(수술후)	●	●

(1) 임상시험 참여 전 5년 이내 병력을 수집한다.

(2) 스크리닝 방문일로부터 4주 이내에 투여되었거나 현재 투약중인 약물을 수집한다.

(3) 이상반응은 증상이 소실될 때까지 추적해야 하며, 피험자 방문이 어려울 경우 유선으로 추적조사해야 한다. (단, 갑상선절제 후 갑상선 호르몬 기능검사를 통해 갑상선저하증이나 부갑상선기능저하증 등이 확인되면 이에 대한 이상반응은 피험자의 임상시험 마지막 방문까지 추적 조사한다.)

1. 임상시험의 제목

유착방지재 메가쉴드(MegaShield)와 가딕스-에스지(Guardix-SG)의 갑상선 전절제술 후 유착방지효과 및 안전성을 비교 평가하기 위한 다기관, 이중맹검, 비열등, 무작위배정, 전향적 확증 임상시험

2. 임상시험기관의 명칭 및 소재지

	임상기관	소재지	전화
1	연세대학교 의과대학<br>세브란스병원	서울특별시 서대문구 연세로 50	02-2228-5650
2	의료법인 삼성의료재단<br>강북삼성병원	서울특별시 종로구 새문안로 29	02-2001-1730
3	가톨릭대학교<br>서울성모병원	서울시 서초구 반포대로 222	1588-1511

3. 임상시험의 책임자 · 담당자 및 공동연구자의 성명 및 직명

1) 임상 책임 연구자 : 연세대학교 의과대학 세브란스병원 남기현 교수

2) 기관별 연구자

① 연세대학교 신촌 세브란스 병원
시험책임자 : 남기현 (갑상선암센터장)
시험담당자 : 정종주 (갑상선내분비외과 부교수)
시험담당자 : 강예원 (세브란스병원 임상시험센터 연구간호사)
독립적평가자(식도조영술평가) : 조성래 (재활의학과 교수)
독립적평가자(유착점수평가) : 강상욱 (갑상선내분비외과 조교수)

② 의료법인 삼성의료재단 강북삼성병원
시험책임자 : 윤지섭 (갑상선 내분비외과 교수)
시험담당자 : 김은영 (갑상선 내분비외과 임상 조교수)
시험담당자 : 김혜정 (강북삼성병원)
독립적평가자(식도조영술평가) : 김미성 (영상의학과 교수)
독립적평가자(유착점수평가) : 박상훈 (외과 임상강사)

③ 가톨릭대학교 서울성모병원
시험책임자 : 배자성 (갑상선암센터장)
시험담당자 : 김광순 (갑상선 내분비외과 임상강사)
시험담당자 : 유지은 (갑상선내분비외과 연구간호사)
독립적평가자(식도조영술평가) : 전소연 (재활의학과 임상강사)
독립적평가자(유착점수평가) : 김정수 (갑상선 내분비외과 교수)

4. 임상시험용 의료기기를 관리하는 관리자의 성명 및 직명

임상 기관	성 명	부서
연세대학교 의과대학 세브란스병원	김진경	갑상선 내분비외과
의료법인 삼성의료재단 강북삼성병원	김은영	갑상선 내분비외과
가톨릭대학교 서울 성모병원	배자성	갑상선 내분비외과

5. 임상시험을 하려는 자의 성명 및 주소

5.1 의뢰자

회사명	대표	소재지	연락처
㈜엘앤씨바이오	이환철	경기도 성남시 중원구 둔촌대로 474 선텍시티 1 차 605,606,607 호	031-731-7050

5.2 임상시험수탁기관(모니터 업무)

회사명	대표	소재지	전화
서울 CRO	김 존	서울시 강남구 봉은사로 6 길 10, 4-6 층, 06123	02-3447-0181

6. 임상시험의 목적 및 배경

6.1 임상시험의 목적

수술 후 유착방지재의 투여가 수술 부위와 주변 조직의 유착 방지 효과가 있음은 널리 알려져 있다. 본 임상 시험에서는 메가쉴드(Megashield)의 갑상선 수술 후 유착 방지 효과를 평가하기 위해, 갑상선 전절제 환자에서 메가쉴드(Megashield) 또는 가딕스-에스지(Guardix-SG)를 갑상선 수술 후 적용하여 유착방지 효과 및 안전성을 비교 평가하고자 한다.

6.2 임상시험의 배경

유착(Adhesion)이란 염증, 창상, 마찰, 수술 등에 의한 상처의 치유 과정에서 섬유조직이 과도하게 생성되거나, 혈액이 유출되어 응고하여, 서로 분리되어 있어야 할 주변 장기 또는 조직이 서로 달라붙는 현상을 의미하는 것이다. 유착 현상은 일반적으로 모든 종류의 수술 후 발생할 수 있으며, 과도한 유착이나 의도하지 않은 다른 장기 및 조직과의 유착은 장기의 기능

장애를 초래하고 경우에 따라서는 유착 박리 재수술이 필요하며 생명을 위협하는 요인이 되기도 한다. 이러한 유착에 의하여 발생할 수 있는 후유증의 종류는 매우 다양하다. [1-4] 미국의 통계 자료에 의하면, 수술 후 유착에 의하여 발생하는 주된 증상으로서, 소장 폐색이 49~74%, 불임이 15~20%, 만성 골반증이 20~50%[5-6], 후속 수술 시 장천공이 19% 정도에 이르는 것으로 알려져 있다. [7]

유착형성의 일반적 예방법으로는 수술 시 유착을 최소화하는 방법, 염증성 반응과 유착 형성에 필요한 병리 생리학적 과정에서 유착기전에 근거하여 항염증제, 혈액응고 방지제, 섬유소 용해제, 항생제 등의 약물치료로 억제하는 방법, 수술 후 유착방지재를 사용하여 상처 부위를 감싸거나 덮어줌으로써 주변조직과의 접촉을 차단시켜 유착을 방지하는 방법이 있지만[8-11], 이러한 방법들은 유착형성을 줄일 수는 있지만 없앨 수는 없는 한계가 있어 많은 주의가 필요하다.

수술 후 유착 방지는 합병증을 줄일 수 있는 매우 중요한 요인이므로 이를 위해 세심한 수술적 방법과 유착방지재가 사용되고 있으나 현재 유착방지의 효과가 아주 뛰어난 독보적인 제품은 없는 실정이다. [6,8]

기존 유착방지재 중 용액 타입의 경우, 체내 적용 시 흘러내림 현상이 심하여 상처에 정확히 도포되기 어렵거나 너무 일찍 분해되어 유착방지 기능을 제대로 하지 못하는 경우가 많고,

필름 혹은 멤브레인 타입의 경우, 내부 장기 적용 시 부착성이 떨어지고 이물반응 있으며 필름의 접힘과 붙는 성질로 최소 침습법이나 복강경에 적용하기 어려움이 있고 봉합이 필요하며 봉합부위에 유착이 발생한다. 젤 타입은 상처가 치유되기 전에 녹아서 배출되어 상처 조직에 머무르는 시간이 부족하여 유착 방지 효과를 제대로 나타내지 못하고 비생체 유래 물질들은 생체 내에서 이물반응이 있다.[8]

유착방지재의 조건으로는 우선 안전하고 효과가 있어야 하고 손상 조직의 치유 기간동안 남아 있으면서 상처 인접 조직들과 섬유조직이 형성되지 못하도록 유착 방지 작용을 하다가 이후에는 자연스럽게 분해, 흡수 및 제거되어야 한다[12]. 또한 유착방지 기능을 확실하게 하기 위해서는 수술 시 발생하는 상처부위에 지속적으로 잘 부착할 수 있도록 부착성이 우수하여야 하고 최소 침습수술법이나 복강경수술 등에도 적합하도록 주사 가능한 제형이어야 한다.

최근 이러한 4 가지 조건을 만족시키도록 온도감응성 고분자를 응용한 유착방지재들이 출시되고 있는데, 조성물의 주요 성분은 대표적인 온도감응성 고분자 Poloxamer 407(Pluronic F-127), Poloxamer 188(Pluronic F-68)이고, 온도감응성 고분자와 소디움 알지네이트(sodium alginate)와의 혼합 조성 혹은 온도감응성 고분자와 젤라틴, 키토산 등의 혼합 조성물들이 그 대표적인 예이다. (한국등록특허공보 제 10-1452041 호 및 제 10 호-1330652 호).

Poloxamer 는 특정 농도 이상에서 자체-집합성이 있어 졸(sol)-젤(gel)전이 온도를 갖는 특이한 성질을 가지며, 생체 적합적이고 독성이 적다[13]. 또한, 소디움 알지네이트(sodium alginate)는 갈색 해초에서 추출된 천연 다당류이며, β-D-mannuronate 와 α-L-glucuronate 로 구성되어 있고, 칼슘 이온과 가교에 의한 젤을 형성하고, 면역학적 활성이 없으며, 동물세포에 의해 소화되지 않는다.

이러한 온도감응성 조성물들은 상온에서는 용액상태로 존재하고, 체온 근처에서는 점성이 있는 젤 상태로 존재함으로써 사용 편리성을 만족시키고 있으나, 여전히 유착방지 기능 및 체내 지속성, 부착성 등에 대해서는 보다 더 장기적인 모니터링이 필요한 실정이다.[12,14]

이러한 배경하에서, 본 임상 시험 의뢰자는 졸-겔 상전이 (sol-to-gel phase transition)가 가능한 온도감응성, 사용자 편의적인 사용 편리성, 우수한 생체 적합성과 체내 지속성 조직 부착성 및 생분해성을 통하여, 궁극적으로 뛰어난 유착방지 효과를 나타내는 유착방지 조성물을 개발하기 위하여 노력하였다. 그 결과, 콜라젠을 주성분으로 하는 입자형 무세포 진피와 히알루론산 및 온도감응성 고분자가 혼합된, 인체 조직 기반 온도감응성 유착방지재 메가쉴드(MegaShield)를 개발, 완성하게 되었다. 메가쉴드(MegaShield)의 장점은 다음과 같다. 즉, 체온에 의해 졸 상태에서 겔 상태로 상전이되어 조직 내에 안정하게 도포될 수 있어 사용이 편리하고, 우수한 생체 적합성과 체내 지속성, 조직 부착성 및 생분해성을 나타내어 효과적으로 유착 발생을 저해할 수 있다. 따라서 메가쉴드(MegaShield)는 차세대 유착방지재로서 사용 편리성 및 유착방지 기능성을 모두 갖춘 제품이라 할 수 있다. 메가쉴드(MegaShield)의 원자재 중 하나인 무세포동종진피는 많은 외과적 치료에서 유착방지의 효과를 가진다는 보고가 되고 있으며 갑상선 수술 후 무세포동종진피를 이식하였을 때 유착방지 효과가 있다는 연구결과가 있다.[15] 또한 메가쉴드(MegaShield)의 유착방지 효과는 Rat 모델의 동물 실험을 통해 유착방지재로서의 기능성을 충분히 갖추고 있음을 증명하였다. 본 임상시험을 통해 메가쉴드(MegaShield)의 효과와 안전성을 확인하고자 한다.

7. 임상시험용 의료기기의 개요 (사용목적, 대상질환 또는 적응증)

사용목적 : 메가쉴드(Megashield)는 육안으로 보이는 모든 갑상선 조직을 제거하는 갑상선 전절제술 후[16] 유착방지를 위해 사용하고자 개발되는 제품이다. 메가쉴드(Megashield)는 졸-겔 전이가 가능한 생체적합성이 높은 수용성 고분자 조성물 제제를 혼합한 용액으로 상온에서는 졸(액체)로 존재하고 인체 내에 적용된 후 체온에 도달하면 점성이 높은 겔 상태로 된다. 이것은 물리적 장벽(physical barrier)으로서 조직의 상처가 치유되는 동안 인접한 조직 혹은 장기사이에 윤활성 표면을 제공함으로써 유착이 형성되는 것을 방지하는 효과를 나타낸다.

대상질환 : 갑상선 전절제술을 시행한 환자

8. 임상시험 의료기기의 적용 대상이 되거나 대조군에 포함되어 임상시험에 참여하는 사람(이하 "피험자"라 한다)의 선정기준·제외기준·인원 및 그 근거

8.1 선정 기준

모든 피험자는 아래의 기준에 모두 부합할 때에만 임상시험에 참여할 수 있다.

(1) 연구참여에 대한 피험자 서면동의에 자발적으로 서명, 날인한 자
(2) 연령이 만 20세 이상, 70세 미만인 환자
(3) 임상시험기간 동안 참여 가능한 환자
(4) 갑상선 질환으로 갑상선 전절제술이 필요한 환자

(5) 갑상선 질환과 관련된 갑상선 절제에 대한 최초 수술 예정인 환자
(6) 수술 전 검사 상 간 기능 이상 및 빈혈, 신장 기능 저하 등이 없는 환자
(7) 임상시험 의료기기 적용 후 임상시험 참여 기간동안 피임에 동의한 자

8.2. 제외 기준

다음 사항 중 하나라도 해당하는 피험자는 임상시험에서 제외한다.

1) 임신 중이거나 수유 중인 여성 혹은 임상 시험용 제품 적용 후 1개월 이내에 임신할 여성
2) 중대한 간장 혹은 신장 질환이 있는 자
3) 림프액 혹은 혈액 응고질환이 있는 환자 혹은 항혈액응고제를 투여하는 자
4) 당뇨용 경구적 혹은 비경구적 혈당강하제를 투여하는 자
5) 면역 억제된 환자 혹은 자가면역질환이 있는 자
6) 중대한 전신성 질환을 가진 자
7) 동반된 후속 수술이 예정된 환자
8) 기타 암으로 항암 치료중인 환자
9) 다른 유착방지재를 처치하는 환자
10) 연구자가 부적절하다고 판단하는 자 (비협조성 등 포함)

8.3. 피험자 수의 선정

· 시험군(MegaShield 사용군) : 70명
· 대조군(Guardix-SG 사용군) : 70명
· 총 피험자 수 : 140 명

8.4. 산출 근거

갑상선 제거 수술 후 대조군(Guardix-SG)에 비해 시험군(MegaShield)의 식도운동능력 점수 (Marshmallow esophagography score)가 더 낮지는 않을 것으로 가정한다 (non-inferiority test). 즉, 시험군이 대조군에 비해 비열등한지를 검정하기 위한 것으로서 귀무가설은 시험군이 대조군보다 평가변수 평균에 있어서 비열등하지 않다는 것이고, 대립가설은 시험군이 대조군보다 평가변수평균에 있어서 비열등하다는 것이다. [17]

귀무가설 H_0 : $\varepsilon \leq -\delta$ 이고

대립가설 H_a : $\varepsilon > -\delta$ 이다.

$$n_1 = \frac{2\left(z_{\alpha/2} + z_{\beta}\right)^2 \sigma^2}{(\varepsilon - \delta)^2}$$

여기서 , α : significant level $1-\beta$: power, σ^2 : variation,

ε = 두 유착방지제의 식도운동능력점수 평균 차(시험군-대조군), 두 군간 차이가 없다고 가정하여 ε=0 으로 가정

δ = margin(비열등성 한계), 임상적으로 의미가 있다고 생각하는 정도 0.1

비열등성 한계 설정 시 기존 대조약과 위약효과를 모두 고려하기 위한 가정은 다음과 같다.
'새로운 치료법의 효과가 기존 치료법보다는 비열등하다' & '새로운 치료법의 효과가 Placebo보다는 우월하다'
위의 가정을 만족하는 margin 은 다음의 식에 의해 구해진다.

$$\Delta = \frac{r}{1+r}(\theta_A - \theta_P)$$

r 값은 active control 과 placebo 의 차이를 그대로 margin 으로 사용하지 않고 이보다는 작은 차이로 고려하기 위해 [0,1] 사이 값을 갖게 되고 margin 은 historical active control 과 placebo 의 차이에서 [0, 1/2]의 비율을 곱한 값을 갖게 된다.

선행연구[16]에서 대조약(Guardix)과 Placebo의 점수 차이는 2.93-2.73=0.2이므로 r을 1로 가정했을 때, margin은 0.2/2 = 0.1 이 된다.
α (Significance level) 5%,

β (power 80%) 0.2 ,

δ (margin) 0.1,

σ (variation)0.2 로 가정하였을 때의 표본크기는 다음과 같다.

$$n_1 = \frac{2\left(z_{\alpha/2} + z_{\beta}\right)^2 \sigma^2}{(\varepsilon - \delta)^2} = \frac{2(1.96 + 0.84)^2 0.2^2}{(0 - 0.1)^2} = 62.8$$

군당 63 명이 얻어지며 10% 탈락률을 고려하면 군당 70 명, 총 140 명의 피험자수가 요구된다.

8.5 연구 대상자 모집 계획

필요시, 연구대상자 모집 공고문을 원내 게시판에 게시하여 연구대상자를 모집할 수 있다.

9. 임상시험 기간

식품의약품안전처의 임상계획승인일로부터 총 32 개월

- 피험자 모집기간 : 21개월
- 유효성 관찰기간: 3개월

- 통계처리 기간 : 3개월
- 결과보고서 작성기간 : 2개월
- 임상시험심사위원회(IRB) 심사기간 : 3개월

10. 임상시험 방법 (사용량·사용방법·사용기간·병용요법 등)

10.1 임상시험 디자인

□ 다기관, 이중맹검(피험자-평가자), 무작위배정, 비열등성, 전향적 확증 시험
□ 임상시험용 의료기기(시험군) : 메가쉴드(MegaShield)
□ 대조시험용 의료기기(대조군) : 가딕스-에스지(Guardix-SG)
본 임상시험은 시험군과 대조군을 설정하여 다기관, 이중맹검(피험자-평가자), 무작위배정, 비열등성, 전향적 확증 시험이다.
갑상선 전절제술이 필요하다고 판단되는 환자 중, 선정/제외 기준에 적합하다고 판단되는 피험자에 한하여 피험자 식별코드를 부여한다. 또한 사전에 작성된 무작위 배정표에 따라 배정번호 (3자리 수)를 순서대로 부여하고 해당 투여군에 배정한다. 무작위 배정 번호와 피험자 식별코드는 임상시험 기간 동안 피험자 식별코드로 사용된다.
(상세 내용은 11.2.4 피험자 식별코드 부여, 11.3.1 무작위배정 참고)
본 임상 시험은 이중 맹검 시험으로서 피험자와 평가자가 맹검(눈가림)을 유지해야 한다. 평가자는 독립된 평가자로, 수술에 참여하지 않아 피험자에게 어떤 임상시험용 의료기기가 적용되었는지 알 수 없고, 평가시에만 피험자와 접촉하게 되어 맹검이 유지된다. 피험자는 수술 중 전신 마취된 상태에서 배정받은 의료기기가 적용되기 때문에 어떤 임상시험용 의료기기가 적용되었는지 알 수 없으며, 수술 이후에도 알 수 없다. 따라서 피험자와 독립된 평가자는 임상시험 기간 동안 맹검이 유지된다

10.2 임상시험용 의료기기 (시험군)

□ 품목명 : 심부체강창상피복재 (B07070.14, 3등급)
□ 형명(모델명) : AAB010 외 2(메가쉴드 (MegaShield))
□ 제조회사 : ㈜엘앤씨바이오
□ 원자재 : poloxamer 407 + 무세포동종진피분말(Acellular Dermal Matrix) + HA (Sodium Hyaluronate) +1,4- BDDE (Butanediol diglycidyl ether)
□ 형상 · 구조 및 치수 : 주사기에 충진 되어 있는 온도감응성 고분자에 인체 유래 무세포동종진피 분말과 가교 히알루론산이 혼합된 투명 혹은 유백색을 띠는 겔. 2ml/syringe, 3ml/syringe 각 1개
□ 사용목적: 갑상선 수술 시 유착 방지 (감소)
□ 사용방법:
가. 사용 전 준비사항

1) 포장상태의 이상 유무를 확인하고 멸균표시지의 부착여부 및 멸균 상태를 확인한다.
2) 유효기간을 확인한다.
3) 시술자는 취급조작을 충분히 숙지하고서 사용하여야 한다.
4) 사용설명서를 숙지한 후 사용하여야 한다.
5) 최종 사용 의사 및 사용 기관은 사용 전에 적절한 보관 환경에서 제품을 보관해야 한다.

나. 조작방법 및 사용방법
수술 후의 합병증에 의한 위험성을 최소화하기 위해 무균적 기술이 유지되어야 한다.
1) 수술 시 사용된 체내의 세척액을 제거하고, 상처 면에 지혈을 확인한다.
2) 이중 포장을 제거하고 멸균 복장을 착용한 수술 참가자에게 내부 쪽에 들어있는 포장을 오염되지 않도록 주의하면서 건넨다.
3) 내부 포장이 손상되지 않았는지 확인한다.
4) 내부 포장지를 제거한 후 주사기를 꺼내 주사기 앞의 캡을 제거하고 카테터를 장착한다.
5) 주사기 밀대를 천천히 밀어 내용물을 수술 부위에 완전히 도포될 수 있도록 주입해 준다.
6) 한번 사용하고 남은 내용물은 재사용하지 않는다.

다. 사용 후 보관 및 관리방법
본 제품은 1회용이므로, 사용 후 적절한 절차에 따라 폐기처분 한다.

라. 적용 후 제거 또는 분해 :
메가쉴드(MegaShield)의 원료인 poloxamer는 간에서 대사되어 신장을 통해 배설되는 것으로 알려져 있다.[18,19,20] 또한 입자형 무세포 진피는 단백질 분해 효소인 collagenase (Matrix Metalloproteinase, MMP-1)에 의해 생분해 될 수 있고, 히알루론산은 체내에 히알루론산 분해 효소인 Hyaluronidase에 의해 분해되며[21], 분해된 산물은 대부분은 폐를 통하여 호흡기체로 배출되거나 간이나 신장을 통하여 체외로 배출되는 것으로 알려져있다.[22]

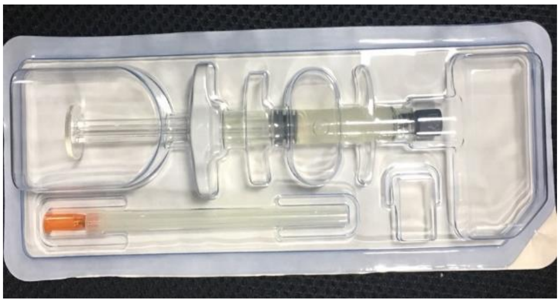

[MegaShield 2ml/syringe]

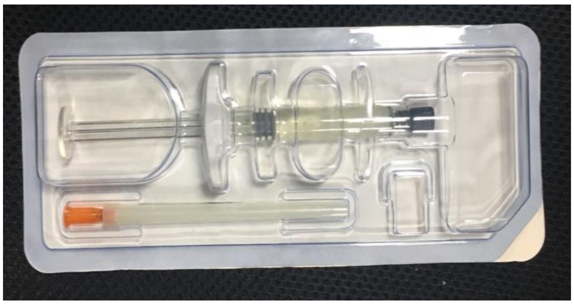

[MegaShield 3ml/syringe]

10.3 대조시험용 의료기기 (대조군)

□ 품목명 : 심부체강 창상피복재 (B07070.14, 3등급)
□ 형명(모델명) : Guardix-SGn-060 (가딕스-에스지(Guardix-SG))
□ 제조회사 : ㈜ 제네웰

□ 원자재 : poloxamer + sodium alginate
□ 형상 · 구조 및 치수 : 주사기에 충진 되어 있는 반투명 흰색의 액체, 6ml /syringe 1개
□ 사용목적: 갑상선 수술 시 유착 방지 (감소)
□ 사용방법:

가. 사용 전 준비사항

1) 사용하기 직전까지 포장을 뜯지 않도록 주의한다.
2) 사용 시 포장은 반드시 멸균된 장소에서 개봉해야 한다.

나. 조작방법 및 사용방법

1) 수술 시 사용된 세척액을 흡인기로 빨아들여 제거한다.
2) 수술 시 상처면에 충분한 지혈이 이루어 진 것을 확인한다.
3) 멸균된 장소에서 본 제품의 포장을 뜯고, 주사기의 마개를 제거한 다음, 카테터 튜브를 돌려 끼운다.
4) Guardix-SG 용액 주입 시 척추, 갑상선 수술 부위에 완전히 도포될 수 있도록 충분히 주입해 준다.

※시험군 의료기기와 동일한 양의 사용을 위해, 수술부위에 적용 시 syringe 의 눈금에 따라 5ml 를 사용하고 1ml 를 잔량으로 남긴다. 연구자는 대조용 의료기기를 5ml 만 사용하였다는 사실을 문서화하며, 의뢰자는 이를 확인하고 관리한다. (5ml 의 사용을 정확히 하기 위하여 수술 전 1ml 를 미리 제거하고 5ml 남은 가딕스를 모두 피험자에게 적용하는 방법도 가능하다.)

다. 사용 후 보관 및 관리방법

1)일회용이므로 투여 후 남은 용약은 폐기하여야 하며 재사용하면 안 된다.

라. 적용 후 제거 또는 분해 : 가딕스-에스지(Guardix-SG)는 온도감응성을 나타내는 원료인 poloxamer를 포함하고 있으며, 이 물질은 위의 시험용 의료기기인 메가쉴드(MegaShield)의 내용과 같이 간에서 대사되어 신장을 통해 배설된다.[18,19,20] 또한 alginate의 칼슘이온은 점차 젤 밖으로 확산되고, alginate는 천천히 분해되며 소변으로 배출되는 것으로 알려져 있다.[23]

10.4 임상 시험 방법

【임상시험 진행 일정표】

수행항목	스크리닝	적용 및 평가		
Visit	Visit 1	Visit 2	Visit 3	Visit 4
방문일(Day)	-30~0	Day 0	Day 7±3	Day 42±7
서면동의서 수령	●			

선정 제외 기준 확인	●			
인구학적조사/신체검사	●			
신체활력증후 검사	●			●
병력/투약력(1)	●			
피험자 등록		●		
수술 및 임상용 의료기기 적용		●		
식도조영술				●
유착점수 평가	●		●	●
소변 β-HCG 검사 (가임여성)	●			●
혈액학적검사	●		●	●
혈청생화학검사	●		●	●
소변 검사	●		●	●
병용약물수집(2)	●	●	●	●
이상반응수집(3)		●(수술후)	●	●

(1) 임상시험 참여 전 5년 이내 병력을 수집한다.
(2) 스크리닝 방문일로부터 4주 이내에 투여되었거나 현재 투약중인 약물을 수집한다.
(3) 이상반응은 증상이 소실될 때까지 추적해야 하며, 피험자 방문이 어려울 경우 유선으로 추적 조사해야 한다. (단, 갑상선절제 후 갑상선 호르몬 기능검사를 통해 갑상선저하증이나 부갑상선기능저하증 등이 확인되면 이에 대한 이상반응은 피험자의 임상시험 마지막 방문까지 추적 조사한다.)

갑상선 전절제술이 필요하다고 판단되는 환자에게 임상시험 참여 동의서를 수령한다. 혈액학적검사, 혈청생화학검사, 요화학 검사 및 선정제외 확인 후 기준에 적합하다고 판단되는 피험자에 한하여 피험자 식별코드를 부여한다. 또한 사전에 작성된 무작위 배정표에 따라 배정번호 (3 자리 수)를 순서대로 부여하고 해당 투여군에 배정한다. 무작위 배정 번호와 피험자 식별코드는 임상시험 기간 동안 피험자 식별코드로 사용된다. 수술 당일에 대상환자는 양측 갑상선전절제술을 시행하고 배정받은 의료기기를 적용한다. 의료기기 적용 시, 시험군의 경우 메가쉴드(MegaShield) 5ml 를, 대조군의 경우 가딕스-에스지(Guardix-SG) 5ml 를 갑상선 절제부위와 띠근육 표면에 도포한다. 수술 후 1 주와 6 주째에 방문하여 유효성 및 안전성을 평가한다.

10.5 병용 금기 약물

시험기간 동안 아래와 같은 의료기기 및 의약품의 사용은 금지된다.

(1) 본 임상시험용 의료기기 외의 다른 유착방지재
(2) 항혈전제/항응고제
(3) 전신적 코르티코스테로이드 제제 : Prednisolone으로 2mg/kg(20mg/day) 또는 등가 용량의 코르티코스테로이드로 7일 이상 지속적으로 사용한 경우

11. 관찰항목 · 임상검사항목 및 관찰검사 방법

【임상시험 진행 일정표】

수행항목	스크리닝	적용 및 평가		
Visit	Visit 1	Visit 2	Visit 3	Visit 4
방문일(Day)	-30~0	Day 0	Day 7±3	Day 42±7
서면동의서 수령	●			
선정 제외 기준 확인	●			
인구학적조사/신체검사	●			
신체활력증후 검사	●			●
병력/투약력(1)	●			
피험자 등록		●		
수술 및 임상용 의료기기 적용		●		
식도조영술				●
유착점수 평가	●		●	●
소변 β-HCG 검사 (가임여성)	●			●
혈액학적검사	●		●	●
혈청생화학검사	●		●	●
소변 검사	●		●	●
병용약물수집(2)	●	●	●	●
이상반응수집(3)		●(수술후)	●	●

(1) 임상시험 참여 전 5년 이내 병력을 수집한다.

(2) 스크리닝 방문일로부터 4주 이내에 투여되었거나 현재 투약중인 약물을 수집한다.
(3) 이상반응은 증상이 소실될 때까지 추적해야 하며, 피험자 방문이 어려울 경우 유선으로 추적 조사해야 한다. (단, 갑상선절제 후 갑상선 호르몬 기능검사를 통해 갑상선저하증이나 부갑상선기능저하증 등이 확인되면 이에 대한 이상반응은 피험자의 임상시험 마지막 방문까지 추적 조사한다.)

□ 유효성 평가

(1) 1차 유효성 평가 : 식도 운동 능력 비교 평가
유착의 정도를 평가하기 위해 수술 6주 후 Marshmallow 식도조영술을 이용하여 시험군과 대조군의 식도운동능력을 비교 평가한다.

(2) 2차 유효성 평가 : 유착 점수 (Adhesion Scores)
스크리닝, 수술 1주 후, 수술 6주 후 설문조사를 통해 유착 점수를 평가하고 시험군과 대조군을 비교한다

□안전성 평가

수술 이후 매 방문 시 모든 이상반응을 평가한다.

11.1. 관찰항목 및 임상검사 항목

<u>1) Visit 1 : 스크리닝 방문 (Day -30~0)</u>

본 임상시험에 참가하기로 동의한 대상자는 임상시험 참여 자격을 확인하기 위해 screening 평가를 수행해야 한다.

- 대상자 서면동의서 수령
- 선정기준/제외기준 검토
- 인구학적 조사/신체검사 : 생년월일, 성별, 몸무게, 키 등
- 신체활력증후 검사 : 혈압, 맥박수
- 병력/투약력 : 당뇨병, 고혈압, 뇌졸중, 허혈성 심질환, 악성종양, 급성감염증, 알러지, 비스포츠포네이트 복용여부 등
- 유착점수 평가
- 소변 β-HCG 검사 (가임 여성일 경우에만 실시)
- 혈액학적 검사 : RBC, Hemoglobin, Hematocrit, Platelet, WBCs, Neutrophil, Lymphocyte, Monocyte, Eosinophil, Basophil, 혈액응고시간(PT/APTT)
- 혈청생화학 검사 : sodium, Potassium, Chloride, Creatinine, BUN, ALT, AST,ALP, Total Bilirubin, Albumin, Total Protein, Total Cholesterol, Glucose
- 소변 검사 : pH, Specific gravity, WBC, Protein, Bilirubin, Glucose, Urobilinogen, Ketone, Nitrite, Blood

▪ 병용약물 수집

2) Visit 2 : Day 0

▪수술 및 임상용 의료기기 적용
▪피험자 등록
▪병용약물 수집
▪이상반응 수집 : 수술 후

3) Visit 3 : Day 7±3

▪유착점수 평가
▪혈액학적 검사 : RBC, Hemoglobin, Hematocrit, Platelet, WBCs, Neutrophil, Lymphocyte, Monocyte, Eosiniphil, Basophil, 혈액응고시간(PT/APTT)
▪혈청생화학 검사 : sodium, Postassium, Chloride, Creatinine, BUN, ALT, AST,ALP, Total Bilirubin, Albumin, Total Protein, Total Cholesterol, Glucose
▪소변 검사 : pH, Specific gravity, WBC, Protein, Bilirubin, Glucose, Urobilinogen, Ketone, Nitrite, Blood
▪병용약물 수집
▪이상반응 수집

4) Visit 4 : Day 42±7

▪신체활력증후 검사
▪식도 조영술
▪유착점수 평가
▪소변 β-HCG 검사 (가임 여성일 경우에만 실시)
▪혈액학적 검사 : RBC, Hemoglobin, Hematocrit, Platelet, WBCs, Neutrophil, Lymphocyte, Monocyte, Eosiniphil, Basophil, 혈액응고시간(PT/APTT)
▪혈청생화학 검사 : sodium, Postassium, Chloride, Creatinine, BUN, ALT, AST,ALP, Total Bilirubin, Albumin, Total Protein, Total Cholesterol, Glucose
▪소변 검사 : pH, Specific gravity, WBC, Protein, Bilirubin, Glucose, Urobilinogen, Ketone, Nitrite, Blood
▪병용약물 수집
▪이상반응 수집

11.2 관찰검사 방법

11.2.1 피험자 동의서 서명

본 임상시험을 실시하기에 앞서 시험자는 피험자 선정 기준에 적합하다고 판단된 피험자에 한해, '피험자 설명서'에 관한 내용을 피험자 본인 또는 법정 대리인 등에게 설명하고 피험자 또는 법정 대리인 등이 내용을 잘 이해한 것을 확인한 다음, 자유의사에 따른 임상시험 참가의 동의를 문서로 받는다. 또한 동의를 서명한 연월일을 증례기록서에 기록한다.

11.2.2 인구학적 조사 및 병력 조사

임상시험에 들어가기 전에 피험자의 인구학적 조사 및 병력/투약력 등에 대하여 면담, 챠트 확인 및 질문 등을 통하여 다음 사항 등을 점검하고 증례 기록서에 기록한다.
-인구학적 조사 : 생년월일, 성별
-신체 검사와 활력 증후: 몸무게, 키, 혈압, 맥박수
-병력 조사 : 당뇨병, 고혈압, 뇌졸중, 허혈성 심질환, 골관절염, 통풍 또는 고요산 혈증, 수면무호흡증, 담낭질환, 요통, 말초혈액 질환 등 수술과 마취에 영향을 줄 수 있는 내과적 질환 등
-검사 : 혈액/혈청생화학/소변 검사 등
-기타 : 약물 복용유무, 흡연력, 음주력, 임신유무 및 계획

11.2.3 피험자 적합성 평가

인구학적 조사, 병력 조사, 과거 임상기록(차트)과 문진 등을 통하여 피험자 선정 및 제외기준에 적합한지 평가한다.

11.2.4 피험자 식별코드 부여

임상시험 참여에 동의하고 피험자 선정 및 제외기준에 적합한 피험자에 한하여 아래의 방법에 따라 피험자 식별코드를 순차적으로 부여하고, "Screening/Enrollment log"를 작성한다. 피험자식별코드는 무작위배정 코드, 환자 이니셜과 함께 식별코드로 사용된다.
- 임상시험실시기관: 세브란스병원 (S01), 강북삼성병원 (S02), 서울성모병원(S03)
- 피험자 식별코드: 실시기관 코드-등록된 순서
예) 세브란스병원에 등록된 피험자: S01-001,S01-002...
강북삼성병원에 등록된 피험자: S02-001, S02-002...
서울성모병원에 등록된 피험자: S03-001, S03-002

11.3 눈가림 배정 방법

11.3.1 무작위 배정

무작위 배정은 블록 무작위 배정방법(block randomization method)에 의해 시험군과 대조군이 1:1비율로 배정될 수 있도록 한다. 임상시험실시기관과 직접적인 관계가 없는 통계 전문가로부터

SAS을 이용하여 작성될 것이다. 눈가림을 유지하기 위해서 시험종료 이전에는 임상시험실시기관과 직접적인 관계가 없는 최소한의 담당자만이 무작위 배정표와 코드를 볼 수 있도록 한다. 이후 시험기관에서는 최종적으로 스크리닝 검사를 통해 선정/제외 기준을 만족한 피험자를 대상으로 순차적으로 무작위 배정 번호(3자리 수)를 부여한다. 이 배정번호는 11.2.4의 피험자 식별 코드와 함께 임상시험 기간 동안 피험자 식별코드로 사용된다.

예: 배정번호 **R 1 0 1**

2, 3 번째 숫자 – 순차적 부여

1 번째 숫자 – 기관번호 (세브란스 1, 강북삼성병원 2, 서울성모병원 3)

11.3.2 눈가림해제(Unblinding)

가. 임상시험 중 눈가림해제 방법

임상시험 중 눈가림해제 방법으로는 눈가림해제 밀봉봉투를 사용한다. 봉투는 빛에 비추었을 때에 그 내용물이 비추어지지 않도록 갈색으로 된 것을 사용하며 그 내용물 확인이 가능하도록 뚫려 있거나 해서는 안 된다. 또한 밀봉된 부분에 서명이 되어 눈가림해제 밀봉봉투 개봉 여부를 확인할 수 있도록 하여야 한다.

나. 임상시험 중 눈가림해제가 되는 상황

① 중대한 이상반응이 발생하였을 때에 처리에 대한 정보가 필요한 경우 눈가림 해제가 수행될 수 있다.

② Suspected Unexpected Serious Adverse Reaction(SUSAR)발생시 눈가림해제가 반드시 수행되어야 한다.

다. 임상시험 중 눈가림해제 절차

① 눈가림 해제 밀봉 봉투는 임상시험 시작 전에 PI에게 전달되어야 하며, 관계자 외 다른 사람의 접근이 불가하며 PI와 가까운 곳에 보관되어야 한다.

② 눈가림 해제될 경우, 해제된 내용은 그 정보가 필요한 의학적 처치를 하는 사람에게 직접적으로 그 내용이 전달되어야 하며, 그 외 다른 연구자나 관련자에게는 필요한 경우를 제외하고는 배정에 대한 내용이 전달되어서는 안 된다.

③ 눈가림 해제 상황 발생시 '눈가림 해제에 대한 문서' 및 근거문서, CRF내에 해당 피험자 및 연구자 정보와 해제일, 해제 사유 등을 기재한다. 또한 눈가림 해제를 실시한 사람의 서명과, 눈가림 해제 날짜, 시간 등이 봉투 겉면에 기록되어야 한다.

④ 의뢰자에게 작성된 '눈가림 해제에 대한 문서'를 이메일 또는 FAX로 전달한다.

⑤ 모든 눈가림 해제 밀봉 봉투는 임상시험 종료 시 PI에 의해서 수거되어야 한다.

라. 임상시험 종료 후 눈가림해제

① 데이터베이스(data base)가 통계분석을 위해 공식적으로 잠금(locked)되기 이전에 눈가림을 유지하여야 한다.

② Blind Review이후 분석군이 확정된 후, 임상시험용 의료기기 제작(또는 라벨링) 담당자는 의료기기 코드번호를 무작위 배정표 생성 담당자에게 전달하고, 무작위 배정표 생성 담당자는 의료기기 코드번호 대신 실제 처리군이 기재된 무작위 배정표(Randomization Code) 를 분석 담당자에게 전달한다.

11.4 독립적 평가자의 평가

시험기관의 식도조영술 (1차 유효성 평가) 및 유착점수 평가자는 눈가림을 유지하면서 식도조영술과 유착점수 설문조사를 시행하고 평가한다. 이 때 독립된 평가자가 객관적으로 판단할 수 있게 하기 위해 독립된 평가자는 피험자의 치료군 배정정보를 알 수 없도록 관련 기록의 눈가림을 유지한다.

11.5 1차 유효성 평가 변수 : Marshmallow 식도 조영술을 이용한 식도 운동능력 비교 평가

갑상선 절제술 및 임상시험용 의료기기 적용 6주 후 환자에게 복와위(prone position) 자세를 취하게 하고, 액체황산바륨(Solitop Sol.140)과 함께 직경 20mm의 marshmallow 덩어리를 삼키도록 지시한 다음 형광투시(fluoroscopy)를 통해 marshmallow의 식도통과를 관찰한다. [24,25,26]
먼저 복와위에서 marshmallow의 식도통과시간을 측정하여, 30초를 기준으로 정상과 비정상으로 구분한다. Marshmallow가 식도의 한 부위에 걸려 30초 이상 진행하지 않는 경우를 정지(impaction)라고 정의하는데, 복와위에서 정지된 경우 환자의 자세를 직립위(standing position)로 바꾸고 침을 삼키도록 한 후 marshmallow의 식도 내 진행을 관찰한다. 이상의 검사로 marshmallow의 식도통과 양상을 다음의 표와 같이 네 등급으로 구분하여 평가한다. [24]

점수	정의	Marshmallow 의 식도통과시간
3	정상	복와위에서 30 초 이내 식도를 통과한 경우
2	경도	복와위에서 통과시간이 30 초를 초과하고 직립위에서 30 초 이내에 통과할 경우
1	중등도	복와위에서 정지하고 직립위에서 30 초 이상에서 통과할 경우
0	중증도	복와위 및 직립위에서 모두 정지할 경우

11.6 2차 유효성 평가 : 유착 점수 (Adhesion Scores)

설문 조사를 통하여 스크리닝시점, 갑상선 절제술 및 임상시험용 의료기기 적용 1주 후, 6주 후 유착 점수(Adhesion Scores)를 평가한다. 모든 설문 문항의 각각의 임상 증상은 점수화 하여, 0점에서 10점의 점수로 측정한다. 점수가 높을수록 유착이 심각함을 의미한다. [24,27]

평 가 항 목	
환자의 주관적 불편감	1. 침 삼킴에 어려움이 있는가?<br>2. 물을 삼키는데 어려움이 있는가?<br>3. 단단한 음식물을 넘김에 어려움이 있는가?<br>4. 환자 본인 생각에 목 주름이 비정상적인가?
시험자의 객관적 평가	5. 환자가 안정을 취하고 있을 때 목의 주름이 대칭적이고 자연스러운가?<br>6. 환자가 젖혔을 때 (neck extension) 목의 주름이 대칭적이고 자연스러운가?<br>7. 외과적 소견으로 염증반응이나 흉터 형성의 정도는?

Neck extension 에 대한 정의 : 피험자가 목을 extension 했을 때 불편하지 않을 정도로 보통 수평에서 30-45 도의 상향으로 턱이 향해 있는 상태

11.7 안전성 평가

수술직후, 수술 1주 후, 수술 6주 후 신체증후, 일반혈액학 검사, 일반혈청생화학 검사, 요화학 검사를 통하여 이상반응 유무를 확인한다.

12. 예측되는 부작용 및 사용시 주의 사항

12.1 예측되는 부작용

1) 일반적인 수술과 관계된 부작용 : 농양, 봉와직염, 창상, 피부괴사, 종창, 혈종, 심혈관질환, 고혈압, 허혈, 혈전, 색전증, 출혈, 마취와 관련된 합병증, 폐합병증, 신경·근육 손상 등.
2) 유착방지재의 사용과 관계된 부작용 : 감염, 알레르기 반응, 통증, 염증반응
3) 갑상선 전절제술과 관계된 부작용 : 부갑상선 기능 저하증, 되돌이 후두 신경 손상, 출혈, 감염, 상부 후두 신경 마비, 호흡곤란 등

예측되는 부작용 발생 시 다음과 같은 경도, 중등도, 중증 등의 판단기준에 따라서 발생된 부작용명 및 그 정도를 이상반응으로 증례기록서에 기록한다.

- <u>경증(Mild)</u> : 피험자의 정상적인 일상생활(또는 기능)을 저해하지 않고, 최소한의 불편을 야기하며, 피험자가 쉽게 견딜 수 있는 경우
- <u>중등도(Moderate)</u> : 피험자의 정상적인 일상생활(또는 기능)을 유의하게 저해하는 불편을 야기하는 경우

▪ 중증(Severe) : 피험자의 정상적인 일상생활(또는 기능)을 불가능하게 하는 경우, 시험의 지속적인 참여가 불가능한 정도, 치료나 입원이 필요할 수 있는 정도

12.2 시험용 의료기기 사용시 주의사항

12.2.1 일반적 주의사항

1) 본 제품을 사용하기 전에 사용방법 및 사용 시 주의사항을 모두 읽어야 한다. 사용방법 및 사용상의 주의사항을 제대로 따르지 않으면 제품이 제 성능이 나오지 않거나 감염 및 부작용 발생을 초래할 수 있다.
2) MegaShield는 외과용품으로서 전문 의료인에 의하여 사용되어야 하며, 사용목적 이외의 사용을 금지한다.
3) 본 제품은 멸균제품이므로 시술하는 동안 멸균상태가 유지되도록 하고 사용기한이 경과하면 사용하지 말아야 한다.
4) 사용 전에 내용물을 점검한 후 포장 및 용기의 손상이나 습기의 흔적이 보이면 사용하지 말아야 한다.
5) MegaShield 용액을 수술 부위에 투입하기 이전에 수술 의사는 세척액 등과 같은 여분의 액체는 흡인기로 빨아들여 제거해야 한다.
6) 환자에게 부작용을 발견하였을 경우, 본 계획서의 “15. 부작용을 포함한 안전성의 평가기준·평가방법 및 보고방법” 을 따르며 병원과 의사는 환자에게 필요한 의학적 조치를 취한다.
7) 유효기간은 제조일로부터 1년이다.

12.2.2 의료기기의 사용 결과 발생할 수 있는 이상반응 사용상의 부주의에 따른 치명적인 부작용사고발생 등에 대한 주의사항

1) 제품 사용 전 포장의 파손 및 손상이 발견되었을 경우 사용되어서는 안되며, ㈜엘앤씨바이오로 연락하여 반환 또는 교환해야 한다.
2) 개봉한 제품을 환자에게 이식하기 전 오염 및 비멸균 상태에 방치되었을 경우 이식에 사용되어서는 안되며, ㈜엘앤씨바이오로 연락하여 반환 처리하거나 의료폐기물로 적절하게 폐기해야 한다.
3) 이식 사용 전 본 제품의 제조시에 사용된 시약 및 용액에 대해 이식받을 환자의 알레르기 반응이 있는지 반드시 확인해야 한다.
4) 본 제품은 제품 라벨에 표시되어 있는 사용기한 내에 사용되어야 하며, 이를 경과한 제품인 경우 ㈜엘앤씨바이오로 연락하여 반환 또는 교환해야 한다.
5) 본 제품의 보관은 직사광선을 피하여 서늘하고 건조한 곳에서 권장하는 보존조건에 따라 보관한다.

12.2.3. 임부, 수유부, 가임여성, 신생아, 유아, 소아, 고령자에 대한 사용

이식 사용 전 본 제품의 제조시에 사용된 시약 및 용액에 대해 이식받을 환자의 알레르기 반응이 있는 경우 사용해서는 안 된다. 환자가 부작용을 일으킬 수 있는 가능성이 있으면 사용해서는 안 된다. 특히 면역력 저하 환자의 경우 주의한다.

12.2.4. 적용상의 주의

1) 본 제품은 실온에서 보관하여 사용되어야 한다.
2) 본 제품은 감마선 멸균 처리된 제품으로 재멸균해서 사용되어서는 안 되며, 한 번 개봉된 제품에 대해서도 재멸균을 하여 재사용해서는 안 된다.
3) 각 개별 포장된 MegaShield는 오직 한 환자에게만 사용해야 한다.
4) 일회용이므로 사용 후 남은 용액을 폐기해야 하며, 재사용 하면 안 된다.

12.2.5. 안전사고의 예방에 필요한 사항

1) 본 제품은 식품의약품안전처의 인증을 받은 GMP 공정을 통해 생산된 제품이므로 본 제품을 사용 시 제조환경과 유사한 적절한 환경(무균실 혹은 수술실) 및 시설에서 사용할 것을 권장한다.
2) 본 제품의 사용 시 환부 또는 시술 부위가 오염되지 않도록 주의한다.

12.3 대조용 의료기기 사용시 주의사항

12.3.1 일반적인 주의사항

1) 본 제품은 멸균제품이므로 시술하는 동안 멸균상태가 유지되도록 하고 유효날짜가 경과하면 사용하지 말 것.
2) 사용 전에 내용물을 점검한 후 포장 및 용기의 손상이나 습기의 흔적이 보이면 사용하지 말 것.
3) GUARDIX-SG 용액을 수술 부위에 투입하기 이전에 수술 의사는 여분의 액체는 흡인기로 빨아들여 제거할 것.

12.3.2 취급 시 주의사항

1) GUARDIX-SG 용액은 실온에서 보관하여 사용되어야 한다.
2) GUARDIX-SG 용액을 체온까지 데워 젤이 될 경우에도 사용할 수 있다.
3) GUARDIX-SG는 멸균되어 제공되며, 재 멸균하여 사용할 수 없다.
4) 일회용이므로 사용 후 남은 용액을 폐기해야 하며, 재사용 하면 안된다.

12.3.3 금기사항

1) GUARDIX-SG 용액은 수술부위에 감염이나 오염이 된 환자에게 적용되지 않도록 한다.
2) GUARDIX-SG 용액은 제품 성분에 대하여 과민증이 있는 환자들에게 이용하면 안된다.
3) GUARDIX-SG 용액은 다른 유착방지 제품, 흡수성 지혈제, 다른 약물들과 조합하여 사용하는 경우, 그 안전성 및 유효성은 동물시험에서 확인되지 않았다.
4) GUARDIX-SG 용액은 임신기간 중에는 사용이 추천되지는 않는다.
5) GUARDIX-SG 용액 사용 후 첫 월경 주기가 끝날 때까지는 임신을 피하기를 권고한다.

12.3.4 예측 부작용

1) 감염 : 발열, 백혈구수 증가
2) 알레르기 반응 : 전신성 발진, 호흡곤란, 부종
3) 통증
4) 염증

13 중지 · 탈락 기준

13.1 중지 기준

1) 임상시험 진행중 관찰되는 상황이 임상시험을 계속 진행하는 것에 무리가 있다고 임상책임연구자가 판단하는 경우
2) 임상시험용 의료기기의 안전성 등의 사유로 임상시험을 중지하고자 할 경우
3) 발생된 이상반응 처치를 위하여 일시적으로 중지되는 경우
4) 발생된 이상반응 처치를 위하여 사용된 의료기기를 제거하여 중지한 경우
5) 중대한 이상반응/이상의료기기반응의 발생으로 중지되는 경우

13.2 중지의 처리

1) 임상시험이 중지된 경우 임상시험책임자는 중지된 시점까지 진행된 피험자에 대한 증례기록서, 임상시험 진행현황 및 결과를 정리하여 임상시험 의뢰자에게 전달하며, 모든 시험관련 자료(증례기록서 및 임상시험용 의료기기)를 임상시험 의뢰자에게 반납하여야 한다. 임상시험이 중지된 자는 안전성·유효성 평가를 위한 통계처리에 포함된다.
2) 임상시험이 조기 종료 또는 일시 중지된 경우 시험책임자는 피험자에게 이 사실을 즉시 알리고 적절한 조치와 추적 관찰이 이루어질 수 있도록 하고, 임상시험심사위원회에 서면으로 통보하여야 한다.

13.3 탈락 기준

1) 피험자 또는 법정 대리권자가 임상시험 참여중단을 요청한 경우
2) 안전성, 유효성 평가에 영향을 줄 수 있는 수술, 약물 또는 다른 의료기기를 병행하여 사용한 경우
3) 중대한 부작용이 발생한 경우
4) 치료방법을 제대로 수행하지 않는 경우
5) 피험자가 동의서에 제시된 사항을 준수하지 않아 평가에 영향을 미치는 경우
6) 피험자의 불참으로 지속적으로 관찰할 수 없는 경우
7) 기타 임상시험담당자가 임상시험 진행에 문제가 있다고 판단되는 경우

13.4 탈락의 처리

1) 피험자가 중도 탈락된 경우 탈락사유 및 탈락 전까지 진행된 임상시험에 관련 자료를 기록, 보관한다.
2) 시험도중에 피험자가 내원하지 못한 경우 피험자의 건강 상태를 확인하고 그 이유를 분명하게 하여야 한다.
3) 중도 탈락된 자는 안전성·유효성 평가시 통계처리에 포함한다.

14 유효성의 평가기준, 평가방법 및 해석방법 (통계분석법에 의함)

유효성의 평가기준, 평가방법 및 해석방법은 2009 년 대한내분비외과학회지에 게재된 임상시험 방법을 바탕으로 한다.[24]

14.1 1차 유효성 평가

본 임상시험의 일차 유효성 평가 변수는 수술 후 6 주째의 Marshmallow 식도조영술을 이용하여 평가된 식도운동능력 점수를 네 등급으로 (0, 1, 2, 3) 분류하여 점수화한다. 통계 분석 방법은 식도운동능력점수의 빈도, 비율 및 평균, 표준편차, 중위수, 최소값, 최대값을 제시하고, 새로운 치료법의 효과가 기존 치료법보다는 비열등한가를 확인하기 위해 두 군 간의 '식도운동능력' 평균 점수 차이(시험군-대조군)에 대한 단측 97.5% 신뢰구간을 사용하여 판단한다. 즉, 단측 97.5% 신뢰구간 하한치가 비열등성한계치(-δ) -0.1 보다 크면 '시험군은 대조군에 비교하여 비열등하다'고 판단한다.

※Marshmallow 식도조영술
먼저 복와위에서 marshmallow 의 식도 통과 시간을 측정하여, 30 초를 기준으로 정상과 비정상으로 구분한다. Marshmallow 가 식도의 한 부위에 걸려 30 초 이상 진행하지 않는 경우를 정지(impaction)라고 정의하는데, 복와위에서 정지된 경우 환자의 자세를 직립위(standing

position)로 바꾸고 침을 삼키도록 한 후 marshmallow 의 식도 내 진행을 관찰한다. 이상의 검사로 marshmallow 의 식도 통과 양상을 다음의 표와 같이 네 등급으로 구분하여 평가한다.

점수	정의	Marshmallow 의 식도통과시간
3	정상	복와위에서 30 초 이내 식도를 통과한 경우
2	경도	복와위에서 통과시간이 30 초를 초과하고 직립위에서 30 초 이내에 통과할 경우
1	중등도	복와위에서 정지하고 직립위에서 30 초 이상에서 통과할 경우
0	중증도	복와위 및 직립위에서 모두 정지할 경우

14.2 2차 유효성 평가

2차 유효성 평가변수는 유착 점수 (Adhesion Scores)이다.
스크리닝시, 갑상선 절제술 및 임상시험용 의료기기 적용 1주 후, 6주 후 아래의 설문조사를 통하여 유착 점수(Adhesion Scores)를 평가한다.

2차 유효성 평가 분석은 시점별 군별 기술통계량(평균, 표준편차, 중위수, 최소값, 최대값)을 제시하고 스크리닝 대비 수술 후 시점별 군별 평균변화량의 기술통계량을 제시한다. 각 시점별 두 군간 평균과 평균변화량은 정규성 가정 만족 여부(Shapiro-Wilk test)에 따라 unpaired t-test, Wilcoxon ranksum test를 적용하고 군내 평균변화량은 paired t-test, Wilcoxon signed rank test를 적용하여 비교한다. 각 통계분석의 유의 수준은 $p<0.05$로 한다.

설문 조사를 통하여 스크리닝시점, 갑상선 절제술 및 임상시험용 의료기기 적용 1주 후, 6주 후 유착 점수(Adhesion Scores)를 평가한다. 모든 설문 문항의 각각의 임상 증상은 점수화 하여, 0점에서 10점의 점수로 측정한다. 점수가 높을수록 유착이 심각함을 의미한다. [24,27]

평 가 항 목	
환자의 주관적 불편감	1. 침삼킴에 어려움이 있는가?<br>2. 물을 삼키는데 어려움이 있는가?<br>3. 단단한 음식물을 넘김에 어려움이 있는가?<br>4. 환자 본인 생각에 목 주름이 비정상적인가?
시험자의 객관적 평가	5. 환자가 안정을 취하고 있을 때 목의 주름이 대칭적이고 자연스러운가?<br>6. 환자가 젖혔을 때 (neck extension) 목의 주름이 대칭적이고 자연스러운가?<br>7.외과적 소견으로 염증반응이나 흉터 형성의 정도는?

Neck extension 에 대한 정의 : 피험자가 목을 extension 했을 때 불편하지 않을 정도로 보통 수평에서 30-45 도의 상향으로 턱이 향해 있는 상태

14.3 통계 분석 대상 집단

본 임상시험의 유효성 분석에서는 mITT set 을 주 분석군으로 하고, PP set 을 보조 분석군으로 한다. 안전성 분석은 ITT set 을 대상으로 한다.

- ITT Set: 본 임상시험에 등록된 모든 피험자 중 임상시험용 의료기기로 적어도 한 번 이상 치료를 받은 피험자를 대상으로 한다.
- Modified ITT Set: 본 임상시험에 등록된 모든 피험자 중 임상시험용 의료기기로 적어도 한 번 이상 치료를 받고 1차 유효성 평가에 참여한 피험자를 대상으로 한다.
- PP set: ITT set 대상자 중 연구계획서의 중대한 위반 없이 본 계획서에 따라 시험 일정이 완료된 피험자를 대상으로 한다.

연구계획서를 중대하게 위반하였다고 고려되어 PP set 에 제외되는 기준은 다음과 같다.

1) 선정/제외 기준 위반
2) 임상시험 기간 중 병용금기약물투여
3) 그 외 중대한 계획서 위반으로 간주할 수 있는 경우

14.4 결측치 처리

유효성 평가에 대한 Modified ITT set 분석 시 결측이 발생할 경우, 일차 유효성 평가 변수는 주 분석군의 정의에 따라 별도의 보정을 하지 않으며 이차 유효성 평가변수는 각 시점별 관찰된 자료 내 분석하며 결측치에 대한 대체처리는 계획하지 않는다. 단, 이차 유효성 평가분석 시 결측치가 분석 자료의 10%이상인 경우는 결측치를 고려한 Repeated Mixed Model 을 적용하여 추가분석 결과를 제시한다.

14.5 인구학적 및 기초자료 분석

인구통계학적 정보는 mITT set 을 대상으로 한다. 피험자의 인구학적 및 기초자료를 연속형 자료(연령, 몸무게, 키, 혈압, 맥박수)는 기술통계량(관측 대상수, 평균, 표준편차, 중앙값, 최소값, 최대값)을 제시하고 범주형 자료(성별, 주요 병력유무, 약물 복용유무, 흡연력, 음주력)는 빈도 및 백분율을 제시한다. 치료군 간의 인구학적 및 기초자료의 비교는 연속형 자료(연령, 몸무게, 키, 맥박수)는 정규성 가정 만족 여부(Shapiro-Wilk test)에 따라 unpaired t-test, Wilcoxon ranksum test 를 실시하여 비교하고, 범주형 자료(성별, 주요 병력유무, 약물 복용유무, 흡연력, 음주력)는 Chi-square test (기대빈도가 5 미만인 cell 이 전체의 20%를 넘는 경우 Fisher's exact test)를 실시하여 비교한다.

15 부작용을 포함한 안전성의 평가 기준 · 평가 방법 및 보고방법

15.1 이상반응의 정의

"이상반응(Adverse Event, AE)"이라 함은 임상시험 중 피험자에서 발생하는 모든 의도하지 않은 증후(sign, 실험실 실험 결과의 이상 등을 포함한다), 증상 또는 질병을 말하며, 해당 임상시험용 의료기기와 반드시 인과관계를 가져야 하는 것은 아니다.
"의료기기이상반응(Adverse Device Effect, ADE)"이라 함은 임상시험용 의료기기로 인하여 발생한 모든 유해하고 의도하지 않은 반응으로서 임상시험용 의료기기와의 인과관계를 부정할 수 없는 경우를 이상반응을 말한다.
"예상하지 못한 의료기기이상반응(Unexpected Adverse Device Effect)"이란 임상시험자자료집 또는 의료기기의 첨부문서 등 이용 가능한 의료기기 관련 정보에 비추어 의료기기이상반응의 양상이나 위해의 정도에서 차이가 나는 것을 말한다.

이상반응에서 다음 사항은 제외된다.
1) 의학적 또는 외과적 처치(예를 들면 수술, 내시경, 발치, 충수절제술) ; 이러한 처치를 초래한 상태가 이상반응임
2) 임상시험 시작시에 이미 존재하거나 발견된 기존의 질병이 임상시험용의약품의 처치 이후 악화되지 않은 경우
3) 악화되지 않은 기존의 질병 치료를 위해 임상시험에 참가하기 이전에 계획된 수술을 위한 입원
4) 건강검진을 위한 입원
5) 미용 목적의 성형수술을 위한 선택적 입원

15.2 중대한 이상반응/의료기기이상반응(Serious AE/ADE) 정의

중대한 이상반응/의료기기이상반응이라 함은 임상시험 중 발생한 이상반응 또는 의료기기 이상반응 중에서 다음 사항의 어느 하나에 해당하는 경우를 말한다.
1) 사망하거나 생명에 대한 위험이 발생한 경우
2) 입원할 필요가 있거나 입원 기간을 연장할 필요가 있는 경우
3) 영구적이거나 중대한 장애 및 기능 저하를 가져온 경우
4) 태아에게 기형 또는 이상이 발생한 경우

15.3 이상반응의 평가

이상반응은 임상시험책임자 또는 임상시험 담당자가 평가한다.

15.3.1 중증도 평가

이상반응이 발생하면 다음의 중증도(severity) 평가기준에 의해 보고하여야 한다.

· 경증 (mild) : 피험자의 정상적인 일상생활(또는 기능)을 저해하지 않고, 최소한의 불편을 야기하며 피험자가 쉽게 견딜 수 있는 경우
· 중등도 (moderate) : 피험자의 정상적인 일상생활(기능)을 유의하게 저해하는 불편을 야기하는 경우
· 중증 (severe) : 피험자의 정상적인 일상생활(기능)을 불가능하게 하는 경우, 시험의 지속적인 참여가 불가능한 정도, 치료나 입원이 필요할 수 있는 정도

15.3.2 임상시험용 의료기기와의 인과관계 평가

이상반응 발현 시 임상시험용 의료기기와의 관련성 여부는 시험자가 다음 기준에 의하여 평가하며, 시험자의 의견을 기술한다.

· 관련성이 명백함 (Definitely related)
-임상시험용 의료기기의 사용과 이상반응 발현의 시간적 순서가 타당한 경우
-이상반응이 다른 어떤 이유보다 임상시험용 의료기기의 사용에 의해 가장 개연성 있게 설명되는 경우
-사용중단으로 이상반응이 없어지는 경우
-재사용(재사용이 가능한 경우에만 실시) 결과가 양성인 경우
-이상반응이 임상시험용 의료기기 또는 동일 계열의 의료기기에 대해 이미 알려져 있는 정보와 일관된 양상을 보이는 경우

· 관련성이 많음(Probably related)
- 임상시험용 의료기기를 사용하였다는 증거가 있는 경우
- 임상시험용 의료기기의 사용과 이상반응 발현의 시간적 순서가 타당한 경우
- 이상반응이 다른 원인보다 임상시험용 의료기기의 사용에 의해 더욱 개연성있게 설명되는 경우
- 임상시험용 의료기기의 사용 중단으로 이상반응이 사라지는 경우

· 관련성이 의심됨(Possibly related)
-임상시험용 의료기기를 사용하였다는 증거가 있는 경우
-임상시험용 의료기기의 사용과 이상반응 발현의 시간적 순서가 타당한 경우
-이상반응이 다른 기능성이 있는 원인들과 같은 수준으로 임상시험용 의료기기의 사용에 기인한다고 판단되는 경우
- 임상시험용 의료기기의 사용의 중단으로 (실시된 경우) 이상반응이 사라지는 경우

· 관련성이 적음(Probably not related)

-임상시험용 의료기기를 사용하였다는 증거가 있는 경우
-이상반응에 대해 보다 가능성 있는 원인이 있는 경우
-임상시험용 의료기기의 사용중단 결과 (실시된 경우)가 음성이거나 모호한 경우
-임상시험용 의료기기의 재사용 (실시된 경우) 결과가 음성으로 나오거나 모호한 경우

·관련성이 없음(not related)
-임상시험용 의료기기를 사용하지 않은 경우
-임상시험용 의료기기의 사용과 이상반응 발현과의 시간적 순서가 타당하지 않음
-이상반응에 대해 다른 명백한 원인이 있는 경우

· 평가불가능(Unknown)
-정보가 불충분하거나 상충되어 판단할 수 없고 이를 보완하거나 확인할 수 없는 경우

15.4 안전성의 평가기준

본 임상시험에서 이상반응은 임상시험용 의료기기의 적용 전에 관찰되지 않은 증상이 발생하는 모든 바람직하지 않은 의학적 소견을 이상반응으로 분류한다. 예측되는 부작용도 이상반응으로 분류하며 이상반응의 정도를 경증, 중등도, 중증으로 분류한다.

15.4.1 예측되는 부작용

1) 일반적인 수술과 관계된 부작용 : 농양, 봉와직염, 창상, 피부괴사, 종창, 혈종, 심혈관질환, 고혈압, 허혈, 혈전, 색전증, 출혈, 마취와 관련된 합병증, 폐합병증, 신경·근육 손상 등.
2) 유착방지재의 사용과 관계된 부작용 : 감염, 알레르기 반응, 통증, 염증반응
3) 갑상선 전절제술과 관계된 부작용 : 부갑상선 기능 저하증, 되돌이 후두 신경 손상, 출혈, 감염, 상부 후두 신경 마비, 호흡곤란 등

15.5 안전성 평가방법 (통계분석방법)

안전성의 분석군은 ITT set을 대상으로 한다. 시험기간동안 보고된 모든 부작용은 군별 발생 명수, 비율 및 건수를 구한다. 시험군과 대조군 간 이상반응 발생율을 chi-square test (혹은 Fisher's exact test)로 분석한다.
이상반응 항목에 대하여 관찰 시점별 군별 기술통계량을 제시한다. 일반혈액학 검사, 일반혈청생화학검사, 요화학 검사 결과 중 연속형 자료는 기술통계량(관측 대상수, 평균, 표준편차, 중앙값, 최소값, 최대값)을 제시하고 범주형 자료는 빈도 및 백분율을 제시한다. 신체증후발생유무는 범주형 자료로 빈도 및 백분율을 제시한다. 필요시 통계분석(chi-square test, Fisher's exact test, unpaired t-test, paired t-test 등)을 적용하여 비교 평가할 수 있다. 각 통계분석의 유의 수준은 $p<0.05$로 한다.

15.6 이상반응 교육 및 보고 방법

15.6.1 이상반응 교육

시험책임자는 시험담당자 및 피험자 또는 대리인에게 임상시험용 의료기기 사용 후 나타날 수 있는 모든 이상반응에 대하여 교육을 실시하고, 사용 후 나타나는 모든 현상에 대해서도 보고하도록 교육을 실시한다.

15.6.2 이상반응의 문서화

임상시험 기간 동안(임상시험용 제품을 처치한 이후) 발생한 모든 이상반응은 임상시험용 제품의 사용과 관계없는 것이라고 하더라도, 대상자의 진료기록부와 증례기록서의 이상반응란에 기록되어야 한다. 임상시험 기간 동안(임상시험용 제품을 처치한 이후)에 발생한 중대한 이상반응은 대상자 진료기록부와 증례기록서의 이상반응이란 그리고 중대한 이상반응 양식에 기입되어야 한다.

각 중대한 이상반응에 대해서 개별적으로 중대한 이상반응 양식을 사용해야 한다. 그러나 초기 보고에서 일시적으로 그리고/혹은 임상적으로 관련 있는 다수의 중대한 이상반응이 있다면, 동일한 중대한 이상반응 양식에 보고될 수도 있다; 이러한 경우 개별적인 중대한 이상반응 보고서는 추적조사 정보가 입수되고 최종 평가/진단이 이루어진 후 제출한다.

시험자는 징후, 증상 그리고/또는 그 밖의 임상적 정보에 기초하여 이상반응에 대한 진단을 내리도록 노력해야 한다. 이 경우에 각 반응마다 징후나 증상이 아닌 진단명을 이상반응으로 기록해야 한다.

임상적으로 유의한 실험실적 이상소견이나 다른 비정상적 평가가 이상반응에 해당된다면, 증례기록서의 이상반응란에 기입한다. 또한 이상반응이 중대한 이상반응의 기준에 해당된다면 중대한 이상반응 양식에도 기입해야 한다. 진단명이 밝혀지면 임상적으로 유의한 실험실적 이상소견보다는 진단명이 증례기록서의 이상반응란과 중대한 이상반응 양식에 기록되어야 한다. 진단명이 밝혀지지 않았다면 실험실적 이상소견을 기록한다.

시험자는 다음과 같이 이상반응의 중증도를 3 단계로 평가한다.

등 급	기 준
경증 (Mild)	피험자의 정상적인 일상생활(또는 기능)을 저해하지 않고, 최소한의 불편을 야기하며 피험자가 쉽게 견딜 수 있는 경우
중등도 (Moderate)	피험자의 정상적인 일상생활(기능)을 유의하게 저해하는 불편을 야기하는 경우
중증 (Severe)	피험자의 정상적인 일상생활(기능)을 불가능하게 하는 경우, 시험의 지속적인 참여가 불가능한 정도, 치료나 입원이 필요할 수 있는 정도

15.6.3 중대한 이상반응/의료기기이상반응 교육

시험자는 임상시험 기간 중 발생한 모든 중대한 이상반응/의료기기이상반응을 임상시험용 의료기기 사용의 관련성 여부와 상관없이 24시간 이내에 의뢰자에게 FAX나 이메일로 보고하여야 한다.

다음 표는 중대한 이상반응의 보고 시한과 필요한 문서양식을 요약 정리한 것이다.

중대한 이상반응 보고양식

	초기보고	추적조사 보고
시 한	24 시간 [a]	추가 정보가 얻어지는 대로 [b]
문 서	■ 의뢰자의 중대한 이상반응 양식에 완벽하게 기입<br>■ 모든 진단검사 결과<br>■ 이상반응 증례기록지<br>■ 의학적 병력/인구통계학적/ 병용약물 증례기록지<br>■ 사망반응의 경우 부검소견서와 사망진단서 추가	■ "추적조사" 보고를 위한 의뢰자의 중대한 이상반응 양식<br>■ 이미 제출된 증례기록지가 갱신된 경우<br>■ 시험자의 이상반응에 대한 최종 & 상세요약<br>■ 필요 시 퇴원 요약지<br>■ 병리 보고, 퇴원요약지, 사후소견, 조직병리학적 결과 등을 포함한 모든 관련된 진단검사결과/보고

a 임상시험실시기관이 이상반응를 알고 난 후 **24 시간 이내**

b 임상시험실시기관이 추가정보를 얻고 난 후 **24 시간 이내**

이외에도 시험자가 중대하다고 간주하거나, 임상시험용 의료기기와의 사용과 연관 지을 수 있는 유의한 위험, 금기, 부작용, 주의사항을 시사하는 사건 등도 중대한 이상반응/의료기기이상반응으로 기록한다.

임상시험계획서에 기술한 기일 내에 상세한 내용이 포함된 추가 보고를 문서로 하여야 한다. 이 경우 피험자의 신원을 보고하기 위하여 피험자의 성명, 주민등록번호 및 주소를 기재하는 대신 피험자 식별코드를 사용하여야 하며, 중대한 이상반응/의료기기이상반응의 보고에 관한 관련 지침이 있는 경우 시험책임자는 이에 따라야 한다.

시험책임자는 안전성 평가에 매우 중요하고 임상시험계획서에 명시된 이상반응/의료기기이상반응이나 실험실 검사치의 이상 등에 대하여 임상시험계획서에 정한 기간 및 보고 방법에 따라 의뢰자에게 보고 한다.

사망 예를 보고하는 경우 시험책임자는 의뢰자와 심사위원회에 부검 보고서(부검을 실시한 경우에 한함)와 사망 진단서 등의 추가 정보를 제공한다.

최종 보고 시에는 가능하다면 다음 정보가 제공되어야 한다.

- 발생시간, 정도, 처치, 경과, 임상시험용 의료기기와의 인과관계 등에 대한 정보를 증례기록서에 기록하여야 한다.

15.6.4 이상반응 발생 시 조치사항

본 임상시험 기간 중 시험책임자, 시험담당자는 환자의 안전에 만전을 기해야 하며, 예측되지 않은 중대한 이상반응/의료기기이상반응 발생시에는 신속하고 적절한 조치를 취하여 이상반응을 최소화하여야 한다.

임상시험 중 '중대한 이상반응/의료기기이상반응' 발생시 각 담당자의 의무는 다음과 같다.

-시험책임자의 의무 :
시험책임자는 임상시험 중 중대한 이상반응/이상의료기기반응이 발생한 때에는 즉시 임상시험위원회 및 의뢰자에게 보고하고 별도의 지시가 있을 때까지 해당 임상시험용 의료기기에 대한 임상시험의 일부 또는 전부를 중지해야 한다.

-시험담당자의 의무 :
시험담당자는 임상시험 실시 중에 중대한 이상반응/의료기기이상반응 등이 발생한 경우에는 즉시 시험책임자 및 의뢰자에게 보고하여야 한다.

-임상시험심사위원회의 의무 :
임상시험심사위원회는 중대한 이상반응/의료기기이상반응을 보고받은 경우 임상시험의 일부 또는 전부에 대하여 시험책임자에게 중지 명령 등 필요한 조치를 하여야한다.

-의뢰자의 의무 :
의뢰자는 시험책임자 또는 시험담당자로부터 중대한 이상반응/의료기기이상반응을 보고받은 경우 시험책임자 또는 시험담당자로부터 제출 받은 보고서를 첨부하여 즉시 식품의약품안전처장에게 제출하여야 하며, 복수의 임상시험실시기관에서 임상시험을 실시하는 경우 해당 임상시험 실시기관에 즉시 보고하여야 한다. 또한 시험책임자가 임상시험심사위원회에 보고하지 않았거나 보고한 사항을 변경할 필요가 있는 경우에는 임상시험심사위원회에도 보고한다.

보고기한은 아래와 같다.
· 사망을 초래하거나 생명을 위협하는 경우: 의뢰자가 이 사실을 보고 받거나 알게 된 날로부터 7 일 이내, 이 경우 상세한 정보를 최초 보고일로부터 8 일 이내에 추가로 보고하여야 한다.
· 그 밖의 중대하고 예상하지 못한 의료기기이상반응의 경우 : 의뢰자가 이 사실을 보고 받거나 알게 된 날로부터 15 일 이내

15.6.5 이상반응의 추적관찰
시험자는 이상반응이 나타난 피험자에 대해 증상이 소실되고 상태가 안정이 될 때까지 피험자를 추적 관찰해야 하며, 이상반응의 이후 진행 과정에 대한 보고서를 제출하여야 한다.
의뢰자는 기보고한 의료기기이상반응의 추가적인 정보가 있는 경우에는 해당 의료기기이상반응이 종결될 때까지 보고해야 한다.

16 피험자 동의서 서식

본 임상 시험은 연구 목적으로 수행되는 임상 시험입니다.
이 설명문 및 동의서는 여러분에게 이번 연구에 대한 정보를 제공하기 위하여 작성되었습니다. 아래의 정보를 신중하게 읽어보시고 주위 분들과 상의를 하십시오. 궁금한 점이 있으면 언제라도 의사나 담당직원에게 문의를 하시면 답변을 얻으실 수 있습니다.

1) 임상시험의 목적과 배경

귀하의 담당의사는 갑상선 전절제술이 필요하다고 판단하였습니다. 본 병원에서는 귀하에게 수술부위의 유착방지를 위해 이미 유착방지 효능이 입증된 제품인 가딕스-에스지(Guardix-SG) 또는 새로운 유착방지재로 개발하는 메가쉴드 (MegaShield) 를 수술부위에 각각 적용하여 메가쉴드 (MegaShield) 의 유착방지 효능을 확인하기 위한 연구를 실시하고자 합니다.
유착방지재는 수술 후 부작용으로 발생하는 유착을 방지하기 위한 의료기기 제품입니다. 갑상선 제거술 후 발생하는 흉터형성과 유착은 갑상샘과 주변 신경의 기능저하 등의 문제점을 야기 시키고, 성대마비, 목 혹은 흉부통증, 삼키는 능력의 저하 등의 부작용을 발생시키며, 심한 경우 이러한 유착을 박리하기 위한 2 차 수술이 요구되기도 합니다. 따라서 이러한 부작용을 최소화 하기 위해 유착방지재를 사용하게 됩니다.

본 임상시험에서 사용될 유착방지재 메가쉴드 (MegaShield)는 온도감응성 고분자에 인체 유래 무세포진피 분말과 가교 히알루론산이 혼합된 유백색 점성 용액으로, 적용하기 전에는 흐름성이 있는 용액(solution) 상태로 유지하고 수술 후 상처 면에 적용하였을 때는 젤(gel) 상태로 되어, 유착이 예상되는 부위에서 장벽 역할을 제공합니다. 메가쉴드(MegaShield)의 원자재 중 하나인 무세포동종진피는 많은 외과적 치료에 유착방지의 효과를 가진다는 보고가 되고 있으며 갑상선 수술 후 무세포동종진피를 이식하였을 때 유착방지 효과가 있다는 연구결과가 있습니다. 또한 메가쉴드(MegaShield)의 유착방지 효과는 쥐를 대상으로 한 동물 실험에서도 유착방지재로서의 기능성을 갖추고 있다는 결과를 얻었습니다. 메가쉴드(Megshield)는 동물 시험에서 부작용없이 안전하게 사용되었으며, 수술 부위에 적용된 이후 체내에서 분해되고 배설되어 안전하다는 시험 결과들이 있습니다. 메가쉴드(Megashiedl)의 원료로 사용되는 물질들은 이미 허가받은 제품들에서 사용되는 성분들이 배합되었다는 점이 이러한 안전성을 더욱 뒷받침할 수 있습니다. 그러나 메가쉴드(MegaShield)는 아직까지 갑상선 전절제술 후 유착방지 효과에 대한 허가 사항은 없습니다. 본 임상 시험을 통해 사람을 대상으로 메가쉴드(MegaShield)의 유효성 및 안전성을 평가하고자 하며, 본 임상 시험은 검증되지 않은 임상 시험입니다.

또한 대조군으로 사용될 유착방지재 가딕스-에스지(Guardix-SG)는 플록사머와 알긴산 나트륨으로 구성된 유착방지재로 이미 임상시험을 통해 갑상선 절제술을 받은 환자에서의 유착방지 효능을 입증한 제품입니다.

2) 임상연구에서 귀하께서 받게 될 각종 검사 및 절차

귀하께서는 갑상선 절제술 후, 수술 부위에 유착방지재 메가쉴드(MegaShield) 또는 가딕스-에스지(Guardix-SG)를 적용하게 됩니다.

본 임상시험의 진행 내용은 다음과 같습니다.

-전체 연구 기간 : IRB승인일로부터 2020년 12월 31일까지
-피험자의 연구 예상 참여 기간 : 6주 (최대 2개월)
-예상 피험자 수 : 총 140 명 (국내 3개 기관 포함 총 인원)
-의료기기 시판 여부 :

- 메가쉴드(시험군) - 시판되지 않음
- 가딕스-에스지 (대조군) - 시판 중

-임상 시험 일정은 다음과 같습니다.

수행항목	스크리닝	적용 및 평가		
Visit	Visit 1	Visit 2	Visit 3	Visit 4
방문일(Day)	-30~0	Day 0	Day 7±3	Day 42±7
서면동의서 수령	●			
선정 제외 기준 확인	●			
인구학적조사/신체검사	●			
신체활력증후 검사	●			●
병력/투약력(1)	●			
피험자 등록		●		
수술 및 임상용 의료기기 적용		●		
식도조영술				●
유착점수 평가	●		●	●
소변 β-HCG 검사 (가임여성)	●			●
혈액학적검사	●		●	●
혈청생화학검사	●		●	●
소변 검사	●		●	●
병용약물수집(2)	●	●	●	●

이상반응수집(3)		●(수술후)	●	●

(1) 임상시험 참여 전 5년 이내 병력을 수집한다.
(2) 스크리닝 방문일로부터 4주 이내에 투여되었거나 현재 투약중인 약물을 수집한다.
(3) 이상반응은 증상이 소실될 때까지 추적해야 하며, 피험자 방문이 어려울 경우 유선으로 추적 조사해야 한다. (단, 갑상선절제 후 갑상선 호르몬 기능검사를 통해 갑상선저하증이나 부갑상선기능저하증 등이 확인되면 이에 대한 이상반응은 피험자의 임상시험 마지막 방문까지 추적 조사한다.)

본 임상시험에서 귀하가 받게 될 검사 및 절차는 다음과 같습니다.

- 시험군과 대조군이 공통으로 받게 될 절차 : 수술 전 기본적인 검사 후, 선정기준에 적합하다고 판단될 경우, 무작위 배정을 통해 가딕스-에스지(Guardix-SG)를 사용하는 대조군과 메가쉴드(MegaShield) 를 사용하는 시험군으로 나뉘게 됩니다. 무작위 배정이란, 동전을 던져 앞면 혹은 뒷면이 나올 확률로 시험군 또는 대조군에 배정 받으신다는 것을 의미하며, 귀하는 귀하가 어떤 제품을 적용하였는지 알 수 없습니다. 수술 당일에 유착방지재 메가쉴드(MegaShield) 또는 가딕스-에스지(Guardix-SG)를 적용하고 1주와 6주 후, 간단한 문진을 통해 수술 부위의 감각 이상이나 통증 등의 불편감과 비정상적 흉터 등에 대한 검사를 하게 됩니다. 또한 일반혈액 검사, 일반생화학 검사, 요화학 검사를 통하여 이상반응, 간기능 검사, 신장 기능 이상 유무를 확인하게 됩니다. 이 검사들을 위해 채취한 검체(혈액과 소변)은 상기 검사 목적으로 병원에서만 시험에 이용되며, 다른 용도로는 사용되지 않습니다. 검사 완료 후 2차적 사용없이 바로 폐기하게 됩니다.

적용 6주후에는 유착방지 효능을 확인하기 위해 식도의 운동능력을 평가하기 위한 마쉬멜로우(Marshmallow) 조영술을 시행하게 됩니다.
또한, 시험기간 동안 매 방문 시 시험 담당의가 귀하가 복용 중이던 약물을 바꾸었는지, 또는 새로운 약물을 복용하였는지 질문할 것입니다.

-시험군과 대조군 간의 상이한 절차 : 시험군과 대조군은 동일한 절차를 통해 임상시험에 참여하게 되며, 수술 시 적용되는 유착방지 제품만 다릅니다.

-마쉬멜로우(Marshmallow) 조영술 : 마쉬멜로우 (Marshmallow) 식도조영술은 조영제가 코팅된 마쉬멜로우 (Marshmallow) 를 복용하고 피험자의 자세에 따라서 식도를 통과하는 시간을 측정하는 방법입니다.

-임상연구를 통해 기대되는 이익 : 유착방지재 메가쉴드(MegaShield) 또는 가딕스-에스지(Guardix-SG)를 제공받게 됩니다. 또한 혈액검사를 비롯한 뇨검사, 일반화학검사 등을 지원받게 됩니다. 본 임상연구에 참여하더라도 피험자는 유착방지효과를 얻거나 얻지 못할 수도 있으나 본 연구를 통해 얻어진 정보는 유착방지재의 갑상선 절제술 후 유착방지효과 및 안전성을 비교 평가하는데 도움을 줄 수 있습니다.

3) 피험자 선정 및 제외

귀하는 다음 사항에 해당될 경우에만 본 임상시험에 참여할 수 있습니다.

(1) 연구참여에 대한 피험자 서면동의에 자발적으로 서명, 날인한 자
(2) 연령이 만 20세 이상, 70세 미만인 환자
(3) 임상시험기간 동안 참여 가능한 환자
(4) 갑상선 질환으로 갑상선 전절제술이 필요한 환자
(5) 갑상선 질환과 관련된 갑상선 절제에 대한 최초 수술 예정인 환자
(6) 수술 전 검사 상 간 기능 이상 및 빈혈, 신장 기능 저하 등이 없는 환자
(7) 임상시험 의료기기 적용 후 임상시험 참여 기간동안 피임에 동의한 자

만약 귀하가 다음의 어느 한가지라도 해당된다면 본 임상시험에서 제외됩니다.

(1) 임신 중이거나 수유중인 여성 혹은 임상 시험용 제품 적용 후 1개월 이내에 임신할 여성
(2) 중대한 간장 혹은 신장 질환이 있는 자
(3) 림프액 혹은 혈액 응고질환이 있는 환자 혹은 항혈액 응고제를 투여하는 자
(4) 당뇨용 경구적 혹은 비경구적 혈당강하제를 투여하는 자
(5) 면역 억제된 환자 혹은 자가면역질환이 있는 자
(6) 중대한 전신성 질환을 가진 자
(7) 동반된 후속 수술이 예정된 환자
(8) 갑상선 암 이외의 암으로 항암치료중인 환자
(9) 다른 유착방지재를 처치하는 환자
(10) 연구자가 부적절하다고 판단하는 자(비협조성 등 포함)

4) 피험자 동의서에 서명할 경우 귀하께서 준수할 사항

만약 귀하께서 본 임상연구에 참여하기로 결정하였을 경우, 본 임상연구에 적합한지를 선별하기 위하여 병명 등 임상적, 방사선학적 소견을 제공하게 됩니다. 임상시험에 등록되면 담당연구자의 지시에 따라 임상시험계획서를 준수하여야 합니다.

5) 임상연구에 사용되는 의료기기의 위험이나 불편함

아래와 같이 의료기기 및 수술에 따른 위험이나 이상반응이 있을 수 있습니다.

▪ 일반적인 수술에 따른 부작용: 농양, 봉와직염, 창상, 피부괴사, 종창, 혈종, 심혈관질환, 고혈압, 허혈, 혈전, 색전증, 출혈, 마취와 관련된 합병증, 폐합병증, 신경·근육 손상 등
▪ 유착방지재의 사용과 관계된 부작용: 감염, 알레르기 반응, 통증, 염증반응
▪ 갑상선전절제술에 따른 부작용: 부갑상선 기능 저하증, 되돌이 후두 신경 손상, 출혈, 감염, 상부

후두 신경 마비, 호흡곤란 등

또한, 마쉬멜로우(Marshmallow) 식도조영술을 시행할 경우, 목 넘김에 불편감이 있을 수 있습니다. 식도조영술은 식도의 기능을 진단하기에 사용되는 일반적인 방법으로서, 본 임상 시험에서도 일반적인 진단을 위한 방사선(X-ray)이 조사됩니다. 따라서 임신부이거나 임신 가능성이 있는 경우 의료진과 상의 후 검사 여부를 결정해야 합니다. 또한, 검사시 사용하는 바륨으로 인해 오심, 구토, 두드러기, 호흡곤란 등이 생길 수 있고 변비가 생길 수 있으므로 검사 후 2~3일간 충분한 수분을 섭취하는 것이 좋습니다.

현재까지 알려지지 않은 부작용이 있을 수 있으며, 연구 재료가 태아나 수유부에 영향이 있을지는 아직 알려지지 않았습니다. 그러므로, 임신 및 수유중인 여성은 이번 연구에 참여하실 수 없습니다. 또한 연구기간 중 임신이 의심되면 담당의사에게 즉시 알려주셔야 됩니다.

6) 임상시험 도중 피험자의 임상시험 참여가 중지되는 경우 및 그 사유

다음의 경우, 연구자 또는 의뢰사는 임상 연구로부터 귀하를 제외하기로 결정할 수 있습니다.

(1) 임상시험 진행중 관찰되는 상황이 임상시험을 계속 진행하는 것에 무리가 있다고 임상책임연 구자가 판단하는 경우
(2) 임상시험용 의료기기의 안전성 등의 사유로 임상시험을 중지하고자 할 경우
(3) 발생된 이상반응 처치를 위하여 일시적으로 중지되는 경우
(4) 발생된 이상반응 처치를 위하여 사용된 의료기기를 제거하여 중지한 경우
(5) 중대한 이상반응/이상의료기기반응의 발생으로 중지되는 경우

7) 귀하께서 임상연구 참여시 받게 될 사례금 관련 사항

귀하가 본 임상시험에 참여하실 경우, 임상시험용 유착방지재를 제공받습니다. 임상 시험과 관련된 검사(혈액 및 일반화학검사, 뇨검사, 마쉬멜로우(Marshmallow) 식도조영술 등)의 검사비는 연구비로 지원되며, 매 방문(스크리닝, 수술 1주후, 수술 6주후)시 5만원, 총 15만원의 교통비를 지급합니다. 지급되는 교통비는 귀하의 연구 참여 정도나 기간에 따라 조정될 수 있습니다. 갑상선 전절제술의 수술비는 지원되지 않습니다.

8) 임상연구에 참여함으로써 귀하(피험자)께 발생할 수 있는 예상 비용

본 임상시험에 참여함으로써 귀하께서 지불해야 하는 비용은 없습니다.

9) 귀하가 선택할 수 있는 다른 치료법이나 종류 및 그 치료법의 잠재적 위험과 이익

귀하께서 받으실 표준치료는 갑상선전절제술 후 시판중인 유착방지재를 사용하는 방법이 있으며, 사용되는 시판중인 유착방지재는 가딕스-에스지(Guardix-SG)와 히알로베리어(Hyalobarrier) 등이 있습니다.

10) 임상연구와 관련된 손상 발생시 귀하께 주어질 보상이나 치료방법

귀하께서 본 임상연구에 참여하여 임상연구에 사용되는 의료기기로 인해 손상을 입을 경우, 배상책임보험증권 및 '피해자 보상에 대한 규약'에 의해 보상을 받게 됩니다. 임상연구와 관련된 상해나 이상반응이 발생하면 귀하는 즉시 임상시험 담당자에게 문의하셔야 합니다.

11) 자발적 참여

귀하께서 본 임상연구에 참여해야 하는 어떠한 법적, 윤리적 의무는 없습니다. 귀하의 본 연구 참여 여부는 귀하의 진료에 어떠한 영향도 끼치지 않을 것입니다. 귀하가 참여를 선택한 경우라도, 언제든지 중도에 참여를 포기하겠다는 의사를 표명하실 수 있습니다. 임상연구를 중도에 포기하시더라도 본 병원에서 계속 치료받는데 있어서 어떠한 불이익도 없을 것이며, 타 환자와 차별없이 동일하게 이루어 질 것입니다.

12) 귀하의 인적사항에 대한 직접 열람

본 임상연구 참여에 동의하신 분에 한하여 임상연구가 타당하게 이루어졌는지 확인하기 위하여 모니터요원, 점검을 실시하는 자, 연구심사위원회 및 식품의약품안전처장은 피험자의 비밀보장을 침해하지 않고 관련규정이 정하는 범위 안에서 임상연구의 실시 절차와 자료의 신뢰성을 검증하기 위하여 귀하의 의무기록을 직접 열람할 수 있습니다. 귀하 또는 법정 대리인이 동의서 서식에 서명하시는 것은 이러한 자료의 직접 열람을 허용함을 의미합니다.

13) 귀하의 인적사항에 대한 비밀보장

본 임상연구의 참여로 인해 귀하의 이름, 주소, 연락처, 주민등록번호, 의료기록, 검사결과, 건강정보가 수집되지만 임상연구로 인해 획득된 임상정보에 연결하기 위한 목적으로만 사용되며, 개인정보보호법에 따라 적절히 관리됩니다. 임상 시험 동의를 철회하게 되는 경우에는, 이미 분석 중이거나 분석이 완료된 데이터에 대한 폐기도 요청할 경우 데이터에 대한 내용도 함께 폐기합니다. 데이터는 잠금장치가 있는 보관 장소로 접근이 제한된 지정된 컴퓨터에 일괄적으로 축적될 예정이며 본 자료로의 접근은 권한이 있는 연구자로 제한하여 타인이 알 수 없게 반드시 보호하도록 최선을 다할 것입니다. 연구 관련 자료는 연구 종료 후 3년간 보관 후 폐기할 것입니다.

귀하의 신원을 파악할 수 있는 모든 기록은 비밀로 보장될 것이며, 임상연구의 결과와 관련된 출판물 등에서 귀하의 신원은 비밀 상태로 유지될 것입니다.

14) 귀하의 안전성에 대한 배려

귀하의 임상연구 지속 참여 의지에 영향을 줄 수 있는 새로운 정보가 수집되면 귀하 또는 대리인에게 즉시 알려드리겠습니다.

15) 임상연구에 대한 질의

본 임상연구 및 귀하의 권익에 관해 추가적인 정보를 얻고자 하거나 임상연구와 관련이 있는 기타 문의사항이 발생한 경우에 아래의 연락처로 연락해 주시기 바랍니다.

임상시험 담당의사 :
소속 :
전화번호 :

임상시험코디네이터 :
소속 :
전화번호

피험자 설명서 원본 및 동의서 사본 1부는 연구에 참여한 본인에게 제공됩니다.
아래에 서명한 본인은 본 연구의 개요와 목적을 환자 또는 대리인에게 충분히 설명하였음을 확인합니다.

담당의사 : (서명)

동 의 서

임상시험 제목: **유착방지재 메가쉴드(MegaShield)와 가딕스-에스지(Guardix-SG)의 갑상선 전절제술 후 유착방지효과 및 안전성을 비교 평가하기 위한 다기관, 이중맹검, 비열등, 무작위배정, 전향적 확증 임상시험**

아래 내용을 읽으시고 내용을 완전히 이해하시면 **네모 칸에 표시**하여 주십시오.

□ 본인은 이 동의서를 읽었고, 내용을 충분히 이해합니다.

□ 본인________________(***피험자가 직접 성명 기재***)은, 담당의사로부터 자세하게 설명을 듣고 궁금한 사항이 있으면 질문을 하였고 적절한 답변을 들었습니다.

□ 본인은 자발적으로 이 연구에 참여합니다.

□ **본인은 이 동의서에 기술된 바에 따라 본인의 건강정보를 사용하고 공유하는 것을 허락합니다.**

□ 본인은 임상시험 외의 다른 치료를 받아야 하거나 이 연구계획서에 따르지 않을 경우, 기타 임상시험 대상자 선정기준에서 제외되는 경우에 이 임상시험에 참여할 수 없다는 것을 알고 있습니다.

□ 본인은 임상시험 기간 중 언제라도 중도에 임상시험 참여를 거부하거나 중단할 수 있습니다. 또 본인은 이 연구 참여를 중단하더라도 본인에게 어떠한 불이익도 없다는 것을 알고 있습니다.

□ 본인은 자유로운 의사에 따라 임상시험참여를 요청하여 동의서 사본 1부를 수령합니다.

□ 본인은 본 시험에 부적절한 사유가 발생하는 등으로 인해 임상시험에서 탈락할 수 있다는 것을 알고 있습니다.

**임상시험 피험자**	성명	________	서명	________	날짜	________
**피험자의 법정대리인** (관계:________)	성명	________	서명	________	날짜	________
**참관인**	성명	________	서명	________	날짜	________
**임상시험 연구자**	성명	________	서명	________	날짜	________

17. 피험자 보상에 대한 규약

본 피험자 보상에 대한 규약은 ㈜엘앤씨바이오가 의뢰하는 모든 임상시험에 참여하여 임상시험에 사용되는 피험자에 대하여 적용되는 보상규약입니다.

17.1 피험자 보상 사유

㈜엘앤씨바이오는 의료기기 임상시험실시기준에 따라 이루어진 임상시험에 있어서 임상 의료기기로 인하여 피험자에게 발생한 유해하고 의도되지 않은 반응에 의한 피험자의 신체상의 손상에 대하여, 다음의 경우에 본 보상규약 및 관련 법률의 규정에 따라 이를 보상합니다.

1) 일시적 통증 또는 쉽게 치료될 수 있는 정도의 손상으로서 시험기관이 이에 대한 치료가 필요하다고 판단하는 경우 (보상범위는 필요한 치료비에 한정함)
2) 입원 또는 입원 기간의 연장이 필요한 경우
3) 지속적 또는 의미 있는 불구나 기능 저하를 초래하는 경우
4) 선천적 기형 또는 이상을 초래하는 경우
5) 사망을 초래하거나 생명을 위협하는 경우

17.2 보상요건

본 보상규약에 따른 피험자 보상은 다음의 요건 하에 이루어집니다.
1) 본 임상시험용 의료기기로 인하여 발생한 신체상의 손상일 것.
2) 시험자가 식품의약품안전처장의 승인을 받은 임상시험계획서의 제반 내용을 준수하였을 것.
3) 시험자의 명백한 과실이나 의무태만에 기인하지 아니하였을 것.
4) 피험자가 시험책임자 또는 시험담당자의 제반 지시사항을 모두 준수하였을 것.
5) 피험자가 당해 신체상의 손상으로 인한 손해의 발생을 최소화하기 위하여 조치를 취하였을 것.

17.3 보상 제외사유

전 17.2조의 규정에 불구하고, 다음의 각 경우에는 본 보상규약에 따른 보상 범위에서 제외됩니다.
1)임상시험용 의료기기로부터 기대된 효과, 효능의 불충분으로 인한 손상
(피험자의 기왕력의 진행 및 악화로 인한 경우를 포함합니다.)
2)피험자의 부주의로 인하여 발생한 손상

17.4 보상기준

1) 예상된 의료기기 이상반응에 대하여 당사자들간에 미리 합의한 보상액 또는 조치가 있는 경우, 당해 기준에 따라 이를 보상합니다.
2) 그 외의 경우에는 신체손상의 정도, 성격, 지속기간, 유사반응 등을 종합적으로 고려하여

당사자들간에 합의한 보상방법에 따라 이를 보상합니다.
3) 당사자들간에 전항의 합의가 이루어지지 아니한 경우에는, 법원의 판결 및 이에 준하는 결정의 확정내용에 따라 보상합니다.

17.5 보상절차

1) 본 보상규약에 따른 신체상의 손상을 입은 피험자는 임상시험의 시험 책임자나 시험기관에 먼저 필요한 의료조치를 요청하여야 합니다.
2) 시험책임자나 시험기관의 조치에도 불구하고 신체상의 손상이 완치되지 아니한 피험자는 의뢰기관에 대하여 이에 대한 보상을 요청할 수 있습니다.
3) 의뢰기관은 위 보상요청을 받은 후 지체 없이 보상대상 해당여부 및 보상기준에 대한 조사를 마치고 이에 관한 내용을 피험자에게 통보합니다.
4) 피험자는 위 통보내용에 대하여 이의가 있는 경우, 위 통보를 받은 날로부터 영업일 [5]일 이내에 이에 대한 이의내용을 의뢰기관에 통보하여야 합니다.
5) 피험자가 제(3)항의 통보를 받고도 이에 대한 이의를 통보하지 아니한 경우, 양 당사자는 위 통보내용에 따른 보상에 합의한 것으로 양해합니다.
6) 피험자가 제(4)항의 규정에 따라 이의를 통보한 경우, 의뢰기관은 피험자에게 위 보상대상 해당여부 및 보상기준에 관하여 판단할 객관적인 전문가를 복수로 추천하고, 피험자를 위 추천일로부터 영업일 [3]일내에 추천인 중 1명을 지명합니다. (피험자가 지명하지 않을 경우 의뢰자가 임의 택일합니다.)

17.6 적용범위

1) 본 보상규약은 의뢰기관이 의뢰하는 모든 임상시험에 참여한 피험자에 대하여 의뢰기관과 피험자간에 다른 약정이 없는 한 그 범위 내에서 일반적으로 적용됩니다.
피험자가 임상시험에 관한 보상에 대하여 의뢰기관의 승인을 받지 아니하고 임상시험과 관련된 다른 제 3자와 체결한 일체의 합의내용은 의뢰기관에 대하여 효력이 없습니다.
폐사는 위의 여러 제반 내용을 참고하여 피험자가 본 임상시험에 의해 어떠한 불이익이라도 받지 않도록 주의하며, 만약 본 임상시험에 의해 문제점이 발생한 경우 피해자 보상규약에 의거하여 책임질 것을 서약합니다.

18 임상시험 후 피험자의 진료에 관한 사항

본 임상시험이 종료된 후, 피험자는 이후의 진료에 관하여 해당 병원의 치료절차에 따르며, 이후의 치료비는 피험자가 지불하여야 한다. 다만 부작용 발생시 임상시험에 사용된 의료기기와의 인과관계 유무를 확인한 후, 본 임상시험에 사용된 의료기기로 인하여 부작용이 발생한 경우 ㈜엘앤씨바이오에서 치료에 관한 부작용이 소실될 때까지 치료비를 지불한다.

19 피험자의 안전보호에 관한 대책

19.1 임상시험실시기관

임상시험실시기관의 장은 해당 임상시험의 실시에 필요한 임상시험실, 설비와 전문인력을 갖추어야 하고, 긴급 시 필요한 조치를 취할 수 있도록 하는 등 해당 임상시험을 적절하게 실시할 수 있도록 하여야 한다.

19.2 임상시험심사위원회

-임상시험심사위원회(Institutional Review Board, 이하 IRB)는 국내 법규/관례에 따라 구성되어 있어야 한다. IRB는 피험자의 권리 · 안전 · 복지를 보호해야 하며, 취약한 환경에 있는 피험자가 임상시험에 참여하는 경우에는 그 이유의 타당성을 면밀히 검토하여야 한다.
- 임상시험심사위원회는 임무를 수행함에 있어 피험자의 시험참가 동의가 적절하게 이루어지지 않았거나 임상시험이 임상시험계획서에 따라 진행되지 않은 경우 또는 중대한 이상반응/의료기기이상반응이 나타난 경우에는 임상시험의 일부 또는 전부에 대하여 중지 명령 등 필요한 조치를 시험 책임자에게 하여야 한다.

19.3 시험자

-시험자(Investigator)라 함은 시험책임자, 시험담당자, 임상시험조정자를 말한다. 시험자는 의뢰자와 합의되고 임상시험심사위원회 및 식품의약품안전처장의 승인을 득한 임상시험계획서는 준수하여 임상시험을 실시하여야 한다.
-임상시험 중 또는 임상시험 이후에도, 시험자는 임상적으로 의미 있는 실험실적 검사치의 이상을 포함하여 임상시험에서 발생한 모든 이상반응에 대해 피험자가 적절한 의학적처리를 받을 수 있도록 조치하여야하고, 시험자가 알게 된 피험자의 병발질환에 대해 의학적 처치가 필요한 경우 이를 피험자에게 알려주어야 한다.
-시험자는 임상시험계획을 정확히 분석 및 숙지하고, 대상 피험자의 문제점을 적극적으로 대응한다.

19.4 의뢰자

-임상연구의 계획 · 관리 · 재정 등에 관련된 책임을 갖고 있는 자로 통상 의료기기 임상시험의 경우 의료기기 제조업자(수입자를 포함한다)를 말한다.
- 임상시험대상, 시험방법, 증례보고서의 서식과 내용 등이 임상시험 계획서의 절차에 따라 이루어지도록 하여야 한다.
-의뢰자의 점검 계획과 절차는 임상시험의 중요도, 피험자 수, 임상시험의 종류와 복잡성, 피험자에게 미칠 수 있는 잠재적인 위험의 정도 및 이미 확인된 임상시험 실시상의 문제점 등에 따라 결정되어야 한다.

19.5 모니터링

-모니터링(Monitoring)이라 함은 임상시험 진행 과정을 감독하고, 해당 임상시험이 임상시험계획서, 표준작업지침서, 임상시험실시기준 및 관련 규정에 따라 실시 · 기록되는지 여부를 검토 · 확인하는 활동을 말한다.
-임상시험에 대한 모니터링은 임상시험모니터요원의 정기적인 임상시험 실시기관 방문과 전화 등을 통해서 이루어 질 것이다. 방문 시 모니터는 환자기록 원본 임상시험용 의료기기 관리 기록, 자료보관(연구파일) 등을 확인한다.
- 또한, 임상시험모니터요원은 임상시험 진행과정을 잘 살피고, 문제가 있을 경우 시험자와 상의한다.

19.6 임상시험계획서의 변경

-임상시험계획서를 임상시험심사위원회 및 식품의약품안전처장으로부터 승인 받은 후,
시험절차가 광범위 해지거나 위험도가 높아지거나 피험자 선정기준에 변화가 있거나 추가적인 안전성 정보로 인해 임상시험계획서를 변경하는 경우에는 임상시험심사위원회 및 식품의약품안전처장의 승인을 받아야 한다.
-임상시험계획서를 수정할 때에는 개정 일자, 개정 이유, 개정 내용 등을 기록하여 보관하여야 한다.
-시험자는 피험자에게 발생한 즉각적 위험 요소의 제거가 필요한 경우를 제외하고는, IRB 및 식품의약품안전처장의 변경승인 이전에는 계획서와 다르게 임상시험을 실시하여서는 안된다. 만일 피험자에게 발생한 즉각적 위험 요소를 제거하기 위해 IRB의 승인을 얻기 전에 이러한 임상시험 계획서의 변경을 적용하게 되는 경우, 가능한 한 빨리 변경에 대하여 IRB(사후 검토 승인을 위하여), 의뢰자, 식품의약품안전처장에게 제출하여야 한다
그리고 IRB 위원장이나 간사가 승인한 문서를 의뢰자에게 보내야 한다.
-임상시험에 영향을 주지 않는 사소한 수정이나 명시는 승인이 반드시 필요한 것은 아니며 행정상 변경이 필요하다.

19.7 피험자 동의

-피험자 동의(Informed Consent)라 함은 피험자가 임상시험 참여 유무를 결정하기 전에 피험자를 위한 설명서를 통해 해당 임상시험과 관련된 모든 정보를 제공받고, 서명과 서명 날짜가 포함된 문서를 통해 본인이 자발적으로 임상시험에 참여함을 확인하는 절차를 말한다.

-피험자 본인 또는 대리인이 동의서 서식, 피험자설명서 및 기타 문서화된 정보를 읽을 수 없는 경우에는 공정한 입회자가 동의를 얻는 전 과정에 참석하여야 한다.

-동의를 얻기 전에 시험자는 피험자 또는 대리인이 임상시험의 세부 사항에 대해 질문하고 해당 임상시험의 참여 여부를 결정할 수 있도록 충분한 시간과 기회를 주어야 하며, 모든 임상시험 관련 질문에 대해 피험자 또는 대리인이 만족할 수 있도록 대답해 주어야 한다.

19.8 피험자 기록의 비밀보장

-피험자의 신원을 파악할 수 있는 기록은 비밀로 보장될 것이며, 임상 시험의 결과가 출판된 경우에도 피험자의 신원을 비밀로 유지한다.
-본 임상시험에 관련된 의뢰자, 모니터요원 및 점검자는 본 임상시험의 모니터링과 점검 및 진행사항 관리를 위한 목적으로 피험자의 기록을 열람할 수 있다. 시험자는 본 임상시험계획서에 서명함으로써, 국내의 법규와 윤리적 측면에서 임상시험 의뢰자 또는 모니터 및 점검자가 피험자의 차트와 증례기록서 기록을 검증하기 위하여 해당 문서를 검토하거나 복사할 수도 있음을 인정한다. 이러한 정보들은 기밀로 보관되어야 한다.
-증례기록서 등 임상 시험에 관련된 모든 서류에는 피험자 이름이 아닌 피험자 식별코드로 기록하고 구분한다.

19.9 기록의 보존

임상시험 실시와 관련된 각종 자료 및 기록을 잘 보존하도록 하여야 하며 보안을 유지하도록 한다. 임상시험결과보고서 작성 완료 이후에는 임상 시험 관련 문서를 임상시험 종료일로부터 3년간 보존하도록 한다.

20 그 밖의 임상시험을 안전하고 과학적으로 실시하기 위하여 필요한 사항

20.1 임상시험용 의료기기의 사용 및 관리

- 임상시험용 의료기기는 본 계획서 4항 "임상시험용 의료기기를 관리하는 관리자의 성명 및 직명" 에서 지정된 의료기기 관리자가 관리한다.
-임상시험용 의료기기는 기재사항에 기술되어 있는 대로 취급, 저장하며 "임상시험용"이라는

문구가 있어야 한다. 임상시험용 의료기기 관리자는 임상시험에 사용되는 의료기기에 대해 인수, 재고관리, 반납 등의 업무를 수행하고 관련 기록을 유지하여야 한다.

20.2 임상시험용 의료기기의 공급과 취급

-의뢰자는 임상시험계획서에 대한 임상시험심사위원회와 식품의약품안전저장의 승인을 얻기 이전에는 임상시험용 의료기기를 관리자 등에게 공급해서는 아니 된다.
-의뢰자는 관리자 등이 임상시험용 의료기기를 취급하고 보관하는 방법에 대해 문서화된 절차를 가지고 있어야 하며, 이 절차에는 적절하고 안전한 인수, 취급, 보관, 미사용 임상시험용 의료기기의 피험자로부터의 반납 및 의뢰자에 대한 반납 등에 대한 방법이 포함된다.
-임상시험용 의료기기를 적시에 공급하여야 하며, 임상시험기관으로의 공급, 임상시험기관의 인수, 임상시험기관으로부터의 반납 및 폐기에 관한 기록을 유지하여야 한다.
-의뢰자는 임상시험용 의료기기에 고장 등 문제가 발생하거나 임상시험의 종료 또는 사용기간의 만료 등에 의한 임상시험용 의료기기의 회수체계를 확립하고 이를 문서화하여야 한다.

21 참고문헌

1. Ellis, H., et al., *Adhesion-related hospital readmissions after abdominal and pelvic surgery: a retrospective cohort study.* The Lancet, 1999. **353**(9163): p. 1476-1480.
2. Lower, A.M., et al., *The impact of adhesions on hospital readmissions over ten years after 8849 open gynaecological operations: an assessment from the Surgical and Clinical Adhesions Research Study.* BJOG: An International Journal of Obstetrics & Gynaecology, 2000. **107**(7): p. 855-862.
3. Weibel, M.-A. and G. Majno, *Peritoneal adhesions and their relation to abdominal surgery: a postmortem study.* The American Journal of Surgery, 1973. **126**(3): p. 345-353.
4. Rosato, L., et al., *Recurrent laryngeal nerve damage and phonetic modifications after total thyroidectomy: surgical malpractice only or predictable sequence?* World journal of surgery, 2005. **29**(6): p. 780-784.
5. Menzies, D. and H. Ellis, *Intestinal obstruction from adhesions--how big is the problem?* Annals of the Royal College of Surgeons of England, 1990. **72**(1): p. 60.
6. Ray, N.F., et al., *Abdominal adhesiolysis: inpatient care and expenditures in the United States in 1994.* Journal of the American College of Surgeons, 1998. **186**(1): p. 1-9.
7. Van Der Krabben, A., et al., *Morbidity and mortality of inadvertent enterotomy during adhesiotomy.* British Journal of surgery, 2000. **87**(4): p. 467-471.
8. Oh, A., *Trends of Anti-adhesion Adjuvant-Review.* Biomater. Res, 2013. **17**: p. 138-145.
9. Burns, J.W., et al., *Preclinical evaluation of Seprafilm bioresorbable membrane.* The European journal of surgery. Supplement.:= Acta chirurgica. Supplement, 1997(577): p. 40-48.
10. Falk, K., et al., *Reduction of experimental adhesion formation by inhibition of plasminogen*

activator inhibitor type 1. British journal of surgery, 2001. **88**(2): p. 286-289.
11. Müller, S.A., et al., *A hydrogel for adhesion prevention: characterization and efficacy study in a rabbit uterus model.* European Journal of Obstetrics & Gynecology and Reproductive Biology, 2011. **158**(1): p. 67-71.
12. Shim, H.S., et al., *Evaluation of resorbable materials for preventing surgical adhesion on rat experiment.* Journal of the Korean Surgical Society, 2002. **63**(3): p. 179-186.
13. Oh SH, Kim JK, Song KS, Noh SM, Ghil SH, Yuk SH, et al. *Prevention of postsurgical tissue adhesion by anti-inflammatory drug-loaded pluronic mixture with sol-gel transition behavior.* J Biomed Master Res 2005;72:306-316.
14. Oh, S.H., et al., *Prevention of postsurgical tissue adhesion by anti-inflammatory drug-loaded pluronic mixtures with sol–gel transition behavior.* Journal of Biomedical Materials Research Part A, 2005. **72**(3): p. 306-316.
15. Kang, S.-W., et al., *Preventive Effect of Human Acellular Dermal Matrix on Post-thyroidectomy Scars and Adhesions: A Randomized, Double-Blinded, Controlled Trial.* Dermatologic Surgery, 2015. **41**(7): p. 812-820.
16. Yi KH, Lee EK, Kang HC, et al., *2016 Revised Korean Thyroid Association Management Guidelines for Patients with Thyroid Nodules and Thyroid Cancer.* Int J Thyroidol 2016 November 9(2): 59-126
17. Chow, S.C.; Shao, J.; *On non-inferiority margin and statistical tests in active control trials,* Statist. Med. 2006; 25:1101–1113
18. Grindel JM, Jaworski T, Emanuele RM, Culbreth P. *Pharmacokinetics of a novel surface-active agent, purified poloxamer 188, in rat, rabbit, dog and man.* Biopharm Drug Dispos 2002;23:87-103.
19. Li C, Palmer WK, Johnston TP. *Disposition of poloxamer 407 in rats following a single intraperitoneal injection assessed using a simplified colorimetric assay.* J Pharm Biomed Anal 1996;14:659-665.
20. Dumortier G, Grossiord, Jean., Agnely, Florence., and Chaumeil, Jean. *A Review of Poloxamer 407 Pharmaceuticaland Pharmacological Characteristics.* Pharm Res. 2006;23(12):2709-2728. 23
21. Karthe Ponnuraj and Mark J. Jedrzejas. *Mechanism of Hyaluronan Binding and Degradation: Structure of Streptococcus pneumoniae Hyaluronate Lyase in Complex with Hyaluronic Acid Disaccharide at 1.7 AÊ Resolution.* J. Mol. Biol 2000, 299, 885-895
22. J. Necas, L. Bartosikova, P. Brauner, J. Kolar. *Hyaluronic acid (hyaluronan): a review.* Veterinarni Medicina, 2008;53(8): 397–411
23. Kataoka K, Suzuki Y, Kitada M, Hashimoto T, Chou H, Bai H, et al. *Alginate, a bioresorbable material derived from brown seaweed, enhances elongation of amputated axons of spinal cord in infant rats.* J Biomed Master Res 2001;54:373-384.
24. Park J.H., et al., *The Efficacy and Safety of Guardix-SG® in Patients Who Are Undergoing Thyroid Surgery: A Randomized, Prospective, Double-blinded Study.* Korean J Endocrine

Surg 2009;9: 127-132

25. 서정건, et al., *원저: Marshmallow 를 이용한 식도조영술의 의의.* 대한소화기학회지, 1996. **28**(3): p. 303-310.

26. 송종원, et al., *식도 운동이상 진단에 있어서 Marshmallow 식도조영술의 임상적 가치.* 대한소화기학회지, 2000. **35**(4): p. 405-412.

27. Park W.S., et al., *Anti-adhesive effect and safety of sodium hyaluronate and sodium carboxymethyl cellulose solution in thyroid surgery.* Asian Journal of Surgery, 2010. **33**(1): p. 25-30

PROTOCOL

SIGNATURE PAGE

유착방지재 메가쉴드(MegaShield)와 가딕스-에스지(Guardix-SG)의 갑상선 절제술 후 유착방지효과 및 안전성을 비교 평가하기 위한 다기관, 이중맹검, 비열등, 무작위배정, 전향적 확증 임상시험

Protocol No. : **LNC-MS-001**

Protocol Ver. : **2.11**

Version Date : **2020-07-06**

(YYYY-MM-DD)

Effective Date :

(YYYY-MM-DD)

Prepared by: ______________________ **Date:** ______________

(YYYY-MM-DD)

Reviewed by: ______________________ **Date:** ______________

(YYYY-MM-DD)

Approved by: ______________________ **Date:** ______________

(YYYY-MM-DD)
