# Supplementary material for: Efficacy and safety of temperature-sensitive acellular dermal matrix in prevention of postoperative adhesion after thyroidectomy: A randomized, multicenter, double-blind, non-inferiority study
Source: PLoS One. 2022 Sep 19;17(9):e0273215. doi: 10.1371/journal.pone.0273215 (PMC9484646; doi:10.1371/journal.pone.0273215)
Supplement: S2 File — (PDF) [file pone.0273215.s004.pdf]

Double-blind, Multi-center randomized, prospective confirmatory clinical study to evaluate the efficacy and safety of MegaShield using for anti-adhesion after total thyroidectomy

Protocol No. : LNC-MS-001

Contents

1. Title 1
2. Name and location of institution 1
3. Name and position of principal investigators, sub-investigators and coordinating investigators 1
4. Name and position of managers who manage investigational products 2
5. Name and address of sponsor and contract research organization 2
5.1 Sponsor 2
5.2 Contract research organization (monitoring) 2
6. Purpose and background 3
6.1 Purpose of clinical trial 3
6.2 Background of clinical trial 3
7. Overview of investigational product (purpose of use, target disease, or indication) 5
8. Inclusion criteria and exclusion criteria for those who are subject to investigational product or are included in the control group and participate in the clinical trial(hereinafter, "subjects"), the number of them and these basis 5
8.1 Inclusion criteria 5
8.2 Exclusion criteria 5
8.3 Number of subjects 6
8.4 Basis for calculation 6
8.5 Subject recruitment plan 8
9. Study period 8
10. Procedure (usage, method of use, period of use, combination therapy, etc.) 8
10.1 Study design 8
10.2 Experimental product (test group) 8
10.3 Comparator (control group) 10
10.4 Study procedure 11
10.5 Contraindication of concurrent use 12
11. Observation and clinical testing items, and their methods 12
11.1 Observation and clinical testing items 14
11.2 Observation and method 15
11.2.1 Signing informed consent 15
11.2.2 Demographic and medical history survey 15
11.2.3 Evaluation on subject eligibility 16

11.2.4 Assignment of subject identification code 16

11.3 Randomization method 16
11.3.1 Randomization 16
11.3.2 Unblinding 16
11.4 Independent evaluator's evaluation 18
11.5 Primary endpoint: Comparison of esophageal movement using Marshmallow esophagography 18
11.6 Secondary endpoint: Adhesion Scores 18
11.7 Safety evaluation 19
12. Predicted side effects and precautions for use 19
12.1 Predicted side effects 19
12.2 Precautions for use of experimental product 19
12.2.1 General precautions 19
12.2.2 Precautions for adverse events which may occur as a result of using the medical device, fatal side effects due to negligence in use, and accidents 20
12.2.3 Use for pregnant women, lactating women, women of childbearing age, newborns, infants, children, and the elderly 20
12.2.4 Precautions on application 21
12.2.5 Matters necessary to prevent safety accidents 21
12.3 Precautions for use of comparator 21
12.3.1 General precautions 21
12.3.2 Precautions for handling 21
12.3.3 Contraindications 21
12.3.4 Predicted side effects 22
13. Discontinuation and drop out 22
13.1 Criteria for discontinuation 22
13.2 Management of discontinuation 22
13.3 Criteria for drop out 23
13.4 Management of drop out 23
14. Evaluation Standard of Efficacy, Its Evaluation Method and Analysis Method (by statistical analysis method) 23
14.1 Primary efficacy endpoint 23
14.2 Secondary efficacy endpoint 24
14.3 Analysis set 25
14.4 Treatment of missing data 25
14.5 Analysis of demographic and basic data 26

15. Discontinuation and drop out 26
15.1 Definition of adverse event 26
15.2 Definition of serious adverse event/adverse device effect (Serious AE/ADE) 27
15.3 Evaluation of adverse event 27
15.3.1 Severity evaluation 27
15.3.2 Assessment of causal relationship with investigational product 27
15.4 Evaluation standard of safety 28
15.4.1 Predicted side effect 29
15.5 Safety evaluation method (statistical analysis method) 29
15.6 Monitoring and reporting system of adverse event 29
15.6.1 Guidance on adverse events 29
15.6.2 Documentation of adverse events 29
15.6.3 Guidance on serious adverse events/adverse device reactions 30
15.6.4 Measures to be taken when an adverse event occurs 32
16. Informed consent form 33
17. Policy on compensation for Subjects 41
17.1 Reason for compensation for subject 41
17.2 Compensation requirements 41
17.3 Reasons for exclusion from compensation 42
17.4 Standard of compensation 42
17.5 Compensation procedure 42
17.6 Application Scope 43
18. Matters concerning the treatment of subjects after clinical trial
19. Measures for the safety protection of subjects 43
19.1 Institution 43
19.2 Institutional review board 43
19.3 Investigator 44
19.4 Sponsor 44
19.5 Monitoring 44
19.6 Revision of protocol 45
19.7 Informed Consent 45
19.8 Confidentiality of subject records 45
19.9 Record keeping 46
20. Other matters necessary for the safe and scientific conduct of clinical trial 46
20.1 Use and management of investigational products 46
20.2 Supply and handling of investigational products 46

21. References......47

【Summary of Clinical Study Protocol】

Title	Double-blind, Multi-center randomized, prospective confirmatory clinical study to evaluate the efficacy and safety of MegaShield using for anti-adhesion after total thyroidectomy
Purpose	The purpose of this study is to evaluate the anti-adhesion effect of MegaShield after thyroidectomy by applying MegaShield or Guardix-SG to patients who have undergone total thyroidectomy to compare anti-adhesion effect and safety.
Sponsor	L&C BIO Inc.
Institution	1. Severance Hospital, Yonsei University College of Medicine<br>2. Kangbuk Samsung Hospital, Sungkyunkwan University School of Medicine<br>3. Seoul St. Mary's Hospital, The Catholic University of Korea
Study Period	32 months from the date of approval (April 2018 ~ December 2020)
Number of Subjects	140 subjects (70 in each of the test group and the control group)
Investigational Device	· Experimental : MegaShield: A transparent or milky gel-type liquid manufactured by mixing acellular dermal matrix powder, cross-linked hyaluronic acid and thermo-sensitive polymer(poloxamer)<br>· Comparator : Guardix-SG: A product consisting of poloxamer and sodium alginate
Inclusion and Exclusion Criteria	<Inclusion Criteria><br>Subjects who meet all of the following criteria shall participate in the clinical trial:<br>1) a patient who gives written informed consent spontaneously;<br>2) a patient who is between 20 and 70 years of age;<br>3) a patient who can participate the clinical trial during the entire study period;<br>4) a patient who is required total thyroidectomy due to thyroid disease;<br>5) a patient who are scheduled to undergo thyroidectomy for the first time related to thyroid disease;<br>6) a patient with no liver dysfunction, anemia or renal inadequacy in preoperative testing; and<br>7) a patient who agreed to contraception while participating in the clinical trial after application of investigational device.<br><Exclusion Criteria>

	Subjects who fall under any of the following criteria shall be excluded from the clinical trial:<br>1) a pregnant or breast-feeding female patient; or a female who plans to become pregnant within 1 month after applying the investigational device;<br>2) a patient with serious liver or kidney disease;<br>3) a patient with lymphatic or hemostatic disorders; or a patient taking an anticoagulant;<br>4) a patient who receives an oral or a parenteral hypoglycemic agent for diabetes;<br>5) a patient immunosuppressed; or a patient with autoimmune diseases;<br>6) a patient with serious systemic disease;<br>7) a patient scheduled for accompanied follow-up surgery;<br>8) a patient undergoing chemotherapy for a cancer other than thyroid cancer;<br>9) a patient who is treated by another anti-adhesion; or<br>10) a patient deemed inappropriate for this study by the investigator (including non-cooperative, etc.).
Study Procedure	Assign patients who meet the inclusion/exclusion criteria according to the randomization table. Perform total thyroidectomy on subjects on the day of surgery, and apply the assigned medical device. When applying the investigational products, apply 5 ml of MegaShield for the test group and 5 ml of Guardix-SG for the control group on the surface of the thyroidectomy area and the strap muscle. Evaluate the efficacy and safety at the visits 1 week and 6 weeks after surgery.
Evaluation Method and Criteria	**Efficacy Evaluation**<br>· Primary efficacy endpoint : Compare the esophageal movement between the two groups using marshmallow esophagography at 6 weeks after surgery to evaluate the severity of adhesion.<br>· Secondary efficacy endpoint : Compare the Adhesion Scores between the two groups at screening, 1 week and 6 weeks after surgery.<br>**Safety Evaluation**<br>Collect all adverse events : At every visit after surgery
Statistics	The main analysis is the mITT set, and the PP set is additionally analyzed. The analysis for the safety is ITT set.<br>· ITT Set: An analysis set targeting for subjects who have received treatment at least once with an investigational product, among all

	subjects enrolled in this clinical trial<br>· Modified ITT Set: An analysis targeting for subjects who have received treatment at least once with an investigational product and participated in the primary efficacy endpoint evaluation, among all subjects enrolled in this clinical trial<br>· PP set: An analysis targeting for subjects who completed the study in accordance with this protocol without any significant violation of the protocol among ITT set<br>The primary endpoint is evaluated by providing the frequency, proportion, mean, standard deviation, median, minimum and maximum of the esophageal movement scores and by using the one-sided 97.5% confidence interval for the difference in the mean score of the 'esophageal movement' between the two groups to determine whether the effect of the new treatment is non-inferior to the existing treatment.<br>The secondary endpoint is evaluated by providing descriptive statistics (mean, standard deviation, median, minimum and maximum) by time point and group, and by providing descriptive statistics of the mean changes, compared to those at the screening, for each time point and each group after surgery.<br>The safety is analyzed on the ITT set. For all adverse events reported in the study period, the number of subjects with adverse events, the proportion, and the number of cases shall be calculated by group. The incidence of adverse events in the test group and control group shall be analyzed by chi-square test (Fisher's exact test).

【Schedule of Clinical Study】

Item	Screening	Application and Evaluation		
Visit	Visit 1	Visit 2	Visit 3	Visit 4
Day	-30~0	Day 0	Day 7±3	Day 42±7
Written consent	●			
Inclusion/exclusion criteria	●			
Demographic survey/physical examination	●			
Vital signs test	●			●
Medical/administering history (1)	●			
Subjects enrollment		●		
Surgery and applying investigational product		●		
Esophagography				●
Evaluating adhesion scores	●		●	●
Urine β-HCG testing (for fertile women)	●			●
Hematological test	●		●	●
Serum biochemical test	●		●	●
Urinalysis	●		●	●
Collecting concomitant drug (2)	●	●	●	●
Collecting adverse event (3)		● (After surgery)	●	●

(1) Collect medical history within 5 years before participating in the clinical trial.

(2) Collect information on drugs that have been administered within 4 weeks of the screening visit or are currently being administered.

(3) Adverse events should be followed up until the symptoms disappear, and if it is difficult for a subject to visit, the follow-up should be performed by phone. (However, if a subject is diagnosed with hypothyroidism or hypoparathyroidism through thyroid function test after thyroidectomy, the resulting adverse events should be followed up until the last visit of the subject for the clinical trial.)

1. Title

Double-blind, Multi-center randomized, prospective confirmatory clinical study to evaluate the efficacy and safety of MegaShield using for anti-adhesion after total thyroidectomy

2. Name and location of institution

	Institution	Location	Telephone
1	Severance Hospital, Yonsei University College of Medicine	50, Yonsei-ro, Seodaemum-gu, Seoul, Republic of Korea	+82-2-2228-5650
2	Kangbuk Samsung Hospital, Sungkyunkwan University School of Medicine	29, Saemunan-ro, Jongno-gu, Seoul, Republic of Korea	+82-2-2001-1730
3	Seoul St. Mary's Hospital, The Catholic University of Korea	222, Banpo-daero, Seocho-gu, Seoul, Republic of Korea	+82-1588-1511

3. Name and position of principal investigators, sub-investigators and coordinating investigators

1) Chief principal investigator : Nam, Kee-hyun, professor, Severance Hospital

2) Investigators of each institution

① Severance Hospital, Yonsei University College of Medicine

Principal investigator : Kee-Hyun Nam

Sub-investigator : Jong Ju Jeong, (Asso. prof. of Thyroid & Endocrine Surgery department)

Coordinating-investigator : Ye-won Kang (Clinical research nurse of Severance Hospital, Clinical Trials Center)

Independent evaluator (Evaluation of esophagography) : Sung-Rae Cho (Prof. of Rehabilitation Medicine department)

Independent evaluator (Evaluation of adhesion scores) : Sang-Wook Kang (Asso. prof. of Thyroid & Endocrine Surgery department)

② Kangbuk Samsung Hospital, Sungkyunkwan University School of Medicine

Principal investigator : Ji-Sup Yun (Prof. of Thyroid & Endocrine Surgery department)

Sub-investigator : Eun Young Kim (Clinical asso. prof. of Thyroid & Endocrine Surgery department)

Coordinating-investigator : Kim, Hye-jeong (Kangbuk Samsung Medical Center)

Independent evaluator (Evaluation of esophagography) : Mi Sung Kim (Prof. of Radiology

department)
Independent evaluator (Evaluation of adhesion scores) : Sang-Hoon Park (Clinical fellow of Surgery department)

③ The Catholic University of Korea, Seoul St. Mary's Hospital
Principal investigator : Ja Seong Bae (Director of Thyroid Cancer Center)
Sub-investigator : Kwangsoon Kim (Clinical fellow of Thyroid & Endocrine Surgery department)
Coordinating investigator : Ji-eun Yoo, (Clinical research nurse of Thyroid & Endocrine Surgery department)
Independent evaluator (Evaluation of esophagography) : So Yeon Jun (Clinical fellow of Rehabilitation Medicine department)
Independent evaluator (Evaluation of adhesion scores) : Jeong Soo Kim (Prof. of Thyroid & Endocrine Surgery department)

4. Name and position of managers who manage investigational products

Institution	Name	Department
Severance Hospital, Yonsei University College of Medicine	Jin kyong Kim	Thyroid & Endocrine Surgery department
Kangbuk Samsung Medical Center	Eun Young Kim	Thyroid & Endocrine Surgery department
The Catholic University of Korea, Seoul St. Mary's Hospital	Ja Seong Bae	Thyroid & Endocrine Surgery department

5. Name and address of sponsor and contract research organization

5.1 Sponsor

Name	CEO	Address	Telephone
L&C Bio Inc.	Lee, Hwan-cheol	Suntechcity suite #605, #606, #607, 474 Dunchon-daero, Jungwon-gu, Seongnam-si, Gyeonggi-do, Republic of Korea	+82-31-731-7050

5.2 Contract research organization (monitoring)

Name	CEO	Address	Telephone
Seoul CRO	John Kim	4-6 fl., 10, Bongeunsa-ro 6-gil, Gangnam-gu, Seoul, Republic of Korea, 06123	+82-2-3447-0181

6. Purpose and background

6.1 Purpose of clinical trial

It is widely known that postoperative administration of anti-adhesion has an effect of preventing adhesion on surgical sites and surrounding tissues. In this clinical trial, the anti-adhesion effect and safety are compared and evaluated by applying either MegaShield or Guardix-SG after thyroidectomy to patients who have undergone total thyroidectomy in order to evaluate the postoperative anti-adhesion effect of MegaShield.

6.2 Background of clinical trial

Adhesion refers to a phenomenon in which surrounding organs or tissues that must be separated from each other adhere to each other due to excessive generation of fibrous tissue or leakage and coagulation of blood during the healing process of wounds due to inflammation, cut, friction, surgery, etc. Adhesion can generally occur after any type of surgery. Since excessive adhesion or unintended adhesion with other organs or tissues causes organ dysfunction, in some cases, Adhesiolysis is required. Adhesion can also be a life-threatening factor. There are many different types of sequelae that can be caused by such adhesion. [1-4] According to statistical data in the United States, it is known that the main symptoms of postoperative adhesion are 49 to 74% in ileus, 15 to 20% in infertility, 20 to 50 in chronic pelvic inflammatory disease [5-6], and 19% in enterobrosia during follow-up surgery. [7]

General prevention of adhesion formation includes: 1) minimizing adhesion during surgery; 2) suppressing adhesion by treating with medication, such as anti-inflammatory drugs, anticoagulants, fibrinolytic agents and antibiotics, in the pathophysiological process necessary for the adhesion formation and inflammatory reactions based on the adhesion mechanism; and 3) preventing adhesion by using anti-adhesions after surgery to cover wound area or block contact with surrounding tissues [8-11]. However, these methods have limitations that can reduce the formation of adhesion but cannot eliminate it, requiring a lot of attention.

Since the prevention of adhesion after surgery is a very important factor in reducing complications, meticulous surgical methods and anti-adhesion are used to prevent adhesion, but there is currently no unique product that has excellent anti-adhesion effect. [6,8]

In the case of the existing liquid type anti-adhesion, it is often difficult to apply accurately to the wound due to severe flowing down when applied in the body, or it breaks down too early to prevent adhesion. In the case of film or membrane type, when applied to internal organs, it has poor adhesiveness, and foreign body reactions appear. In addition, the folding and adhering properties of the film make it difficult to apply to minimally invasive surgery or laparoscope, require sutures, and cause adhesion to the suture area. Since gel types melt and discharge before the wound is healed and there is insufficient time to stay in the wound tissues, it does not properly show anti-adhesion effects and non-biomaterial exhibits foreign body reaction in vivo [8].

Anti-adhesion must be safe and effective first. It must also remain while the damaged tissue is healed, preventing adhesion between tissues adjacent to the wound and the fibrous tissues, and then be naturally decomposed, absorbed and removed [12]. And to ensure anti-adhesion function, it must have excellent adhesion so that it can be continuously adhered to the wound caused by surgery, and it must be an injectable type suitable for minimally invasive surgery or laparoscope.

Recently, anti-adhesion products with thermo-sensitive polymers have been released to meet these four conditions. The main ingredients of the composition are Poloxamer 407 (Pluronic F-127) and Poloxamer 188 (Pluronic F-68), and typical examples include a mixture of thermo-sensitive polymer and sodium alginate, and a mixture of thermo-sensitive polymer, gelatin and chitosan (Korea Patent Publication No. 10-1452041 and No. 10-1330652).

Poloxamer has a unique property of having a sol-to-gel transition temperature due to its self-adhesive above a certain concentration, and it is biocompatible and less toxic [13]. Sodium alginate is a natural polysaccharide extracted from brown algae, consisting of β-D-mannuronate and α-L-glucuronate. It forms a gel by cross-linking with calcium ions, has no immunological activity, and is not digested by animal cells.

These thermo-sensitive compositions are convenient to use because they exist in a liquid state at room temperature and in a viscous gel state near body temperature, however they still require longer-term monitoring for anti-adhesion, in vivo persistence, adhesiveness, etc. [12,14]

Based on this background, the sponsor of this clinical trial strove to develop an anti-adhesion composition with these properties: thermo-sensitivity, which enables sol-to-gel phase transition; user-friendly convenience; excellent biocompatibility; in vivo persistence; adhesiveness to tissue; biodegradability; and ultimately excellent anti-adhesion effect. As a result, it developed MegaShield, a human tissue-derived thermo-sensitive anti-adhesion, which is a mixture of collagen-based granular acellular dermal matrix, hyaluronic acid and thermo-sensitive polymer. MegaShield has the following advantages. It is convenient to use by its phase transition from a sol state to a gel state by body temperature so that it can be stably applied in tissues. It can also effectively inhibit the formation of adhesion because it has excellent biocompatibility, in vivo persistence, adhesiveness to tissues, and biodegradability. Therefore, MegaShield is a next-generation anti-adhesion product that has both convenience of use and anti-adhesion functionality. It has been reported that the

acellular allogenic dermal matrix, one of the raw materials of MegaShield, has the effect of anti-adhesion in many surgical treatments, and studies have shown that transplanting acellular allogenic dermal matrix after thyroidectomy has an anti-adhesion effect [15]. In addition, an animal test on rats demonstrated that MegaShield has sufficient functionality as an anti-adhesion. This clinical trial is intended to verify the efficacy and safety of MegaShield.

7. Overview of investigational product (purpose of use, target disease, or indication)

Purpose of use : MegaShield is a product developed to be used for preventing adhesion after total thyroidectomy, which removes all thyroid tissues visible to the naked eye [16]. MegaShield is a mixed solution with a composition of water-soluble polymers capable of sol-to-gel transition and with high biocompatibility. It exists in a sol (liquid) state at room temperature and becomes viscous gel at body temperature after being applied in the human body. This acts as a physical barrier, which has effect on preventing adhesion from forming by providing a lubricating surface between adjacent tissues or organs while tissue wounds heal.

Target: Patients with total thyroidectomy

8. Inclusion criteria and exclusion criteria for those who are subject to investigational product or are included in the control group and participate in the clinical trial (hereinafter, "subjects"), the number of them and these basis

8.1 Inclusion criteria

Subjects who meet all of the following criteria shall participate in the clinical trial:

(1) a patient who gives written informed consent spontaneously;
(2) a patient who is between 20 and 70 years of age;
(3) a patient who can participate the clinical trial during the entire study period;
(4) a patient who is required total thyroidectomy due to thyroid disease;
(5) a patient who are scheduled to undergo thyroidectomy for the first time related to thyroid disease;
(6) a patient with no liver dysfunction, anemia or renal inadequacy in preoperative testing; and
(7) a patient who agreed to contraception while participating in the clinical trial after application of investigational product.

8.2. Exclusion criteria

Subjects who fall under any of the following criteria shall be excluded from the clinical trial:

(1) a pregnant or breast-feeding female patient; or a female who plans to become pregnant within 1 month after applying the investigational product;
(2) a patient with serious liver or kidney disease;
(3) a patient with lymphatic or hemostatic disorders; or a patient taking an anticoagulant;
(4) a patient who receives an oral or a parenteral hypoglycemic agent for diabetes;
(5) a patient immunosuppressed; or a patient with autoimmune diseases;
(6) a patient with serious systemic disease;
(7) a patient scheduled for accompanied follow-up surgery;
(8) a patient undergoing chemotherapy for a cancer other than thyroid cancer;
(9) a patient who is treated by another anti-adhesion; or
(10) a patient deemed inappropriate for this study by the investigator (including non-cooperative, etc.).

8.3. Number of subjects

- Test group (MegaShield group) : 70 patients
- Control group (Guardix-SG group) : 70 patients
- Total number of subjects : 140 patients

8.4. Basis for calculation

Suppose that the esophageal movement score (Marshmallow esophagography score) of the test group (MegaShield) is not lower than that of the control group (Guardix-SG) after thyroidectomy (non-inferiority test). In other words, this study is to test whether the test group is non-inferior to the control group. The null hypothesis is that the test group is not non-inferior in the mean of parameters to the control group, and the alternative hypothesis is that the test group is non-inferior in the mean of parameters to the control group [17].

Null hypothesis H_0 : $\varepsilon \leq -\delta$, and

alternative hypothesis H_a : $\varepsilon > -\delta$.

$$n_1 = \frac{2(z_{\alpha/2} + z_\beta)^2 \sigma^2}{(\varepsilon - \delta)^2}$$

where, α : significant level $1-\beta$: power, σ^2 : variation,

ε = difference between the means of the esophageal movement scores of the two anti-adhesion products (test group - control group), assuming there is no difference between the two groups (ε=0),

δ = margin (limits of non-inferiority), 0.1 (the degree to which is regarded to be clinically significant)

To take into account both the existing comparator and placebo effect when setting the limits of non-inferiority, assume that:

'The effectiveness of the new treatment is non-inferior to that of the existing one' and 'the effectiveness of the new treatment is superior to the placebo effect.'

The margin that satisfies the above assumptions is obtained by the following expression:

$$\Delta = \frac{r}{1+r}(\theta_A - \theta_P)$$

.

For r, the difference between the active control and placebo is not used as the margin, and [0, 1] median is given to consider that r is smaller than the difference. The margin is multiplied by the ratio of [0, 1/2] to the difference between the historical active control and placebo.

In the previous study [16], the difference in scores between comparator (Guardix) and Placebo is 2.93 - 2,73 = 0.2, so assuming that r is 1, the margin is 0.2/2 = 0.1.

Assuming that:

α (Significance level) is 5%,

β (power 80%) is 0.2,

δ (margin) is 0.1, and

σ (variation) is 0.2,

the sample size is:

$$n_1 = \frac{2(z_{\alpha/2}+z_\beta)^2\sigma^2}{(\varepsilon-\delta)^2} = \frac{2(1.96+0.84)^2 0.2^2}{(0-0.1)^2} = 62.8.$$

Therefore, 63 subjects are calculated per group, and considering 10% drop-out, 70 subjects per group, that is, a total of 140 subjects, are required.

8.5 Subject recruitment plan

If necessary, subjects can be recruited by posting a public notice of recruitment on the bulletin board in the hospital.

9. Study period

32 months from the date of approval of IND by the Ministry of Food and Drug Safety

- Review period by the IRB : 3 months
- Subject recruitment period : 21 months
- Observation period for efficacy : 3 months
- Statistics processing period : 3 months
- Period for creating study report : 2 months
-

10. Procedure (usage, method of use, period of use, combination therapy, etc.)

10.1 Study design

☐ A prospective confirmatory clinical trial of double-blinded (subjects-evaluator), multi-center, randomized and non-inferiority

☐ Experimental product (test group) : MegaShield

☐ Comparator (control group) : Guardix-SG

This is a prospective confirmatory clinical trial of double-blinded (subjects-evaluator), multi-center, randomized and non-inferiority which is performed by setting the test group and the control group. Subject identification codes are assigned only to subjects who are judged to meet the inclusion/exclusion criteria among those who are in need of total thyroidectomy. In addition, subjects are given a randomization code (3 digits) in order according to the randomization table and assigned to the corresponding application group. The randomization code and subject identification code will be used as the subject number during the clinical trial.

(For details, see 11.2.4 Assignment of subject number and 11.3.1 Randomization.)

This clinical trial is a double-blind trial, and both subjects and evaluator should be blinded. The evaluator is an independent evaluator and does not participate in surgery, so it is not possible for him/her to know which investigational product was applied to the subject. And since he/she contacts the subject only during evaluation, blinding will be maintained. Since the subject is applied with the assigned investigational product while under general anesthesia during surgery, he/she cannot know which investigational product has been applied, and does not know even after the surgery. Thus, subjects and the independent evaluator are blinded during the clinical trial.

10.2 Experimental product (test group)

☐ Classification : Dressing, adhension barrier(B07070.14, Class 3)
☐ Model name : 3 items including AAB010 (MegaShield)
☐ Manufacturer : L&C Bio Inc.
☐ Raw material : poloxamer 407 + Acellular Dermal Matrix + HA (Sodium Hyaluronate) +1,4- BDDE (Butanediol diglycidyl ether)
☐ Shape, structure and dimensions : A transparent or milky gel with a mixture of human-derived acellular dermal matrix powder and cross-linked hyaluronic acid in a syringe filling with thermo-sensitive polymer. 1 each 2ml/syringe and 3ml/syringe
☐ Purpose of use: Prevent (or reduce) adhesion at thyroidectomy
☐ How to use:

A. Preparation before use

1) Check the packaging condition for any abnormality, and check whether the sterilization label is attached and sterilization status.

2) Check the expiration date.

3) The operator should fully understand how to handle and operate it before using it.

4) Use it after familiarizing with the instructions.

5) End-use physicians and institutions should store the product in an appropriate storage environment before use.

B. How to operate and use

The aseptic technique should be maintained to minimize the risk of postoperative complications.

1) Remove the cleaning solution used during the surgery from the body and check if the bleeding on the wound surface has stopped.

2) Remove the double packaging and hand the inner package over to the surgical participant wearing sterile clothing, taking care not to contaminate it.

3) Check if the inner package is not damaged.

4) Remove the inner package, take out the syringe, remove the cap of the syringe, and then mount the catheter.

5) Slowly push the plunger to inject the contents so that it can be fully applied to the surgical area.

6) After using once, do not reuse the remaining contents.

C. How to store and control after use

This product is disposable, so dispose of it according to the appropriate procedures after use.

D. Remove or disassemble after application :

It is known that poloxamer, the raw material of MegaShield, is metabolized in the liver and excreted through the kidneys [18,19,20]. In addition, the particulate acellular dermal matrix can be biodegraded by collagenase (Matrix Metalloproteinase, MMP-1), a protease, and hyaluronic acid is degraded by hyaluronidase in the body [21]. Mose of decomposed products are known to

be released as respiratory gas through the lungs or to be emitted to the outside of the body through the liver or kidneys [22].

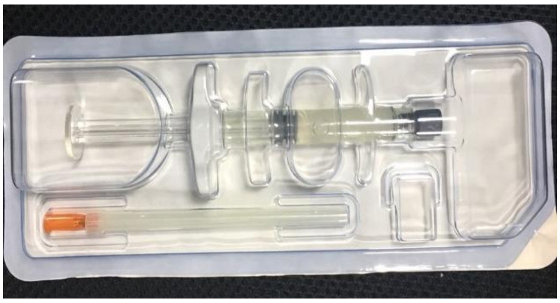

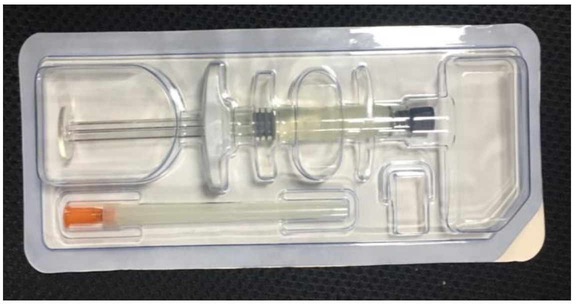

[MegaShield 2ml/syringe] [MegaShield 3ml/syringe]

10.3 Comparator (control group)

☐ Item name : Dressing, adhension barrier(B07070.14, Class 3)
☐ Model name : Guardix-SGn-060 (Guardix-SG)
☐ Manufacturer : Genewel Co., Ltd.
☐ Raw material : poloxamer + sodium alginate
☐ Shape, structure and dimensions : Translucent white liquid filled in a syringe, 1 each 6ml /syringe
☐ Purpose of use: Prevent (or reduce) adhesion at thyroidectomy
☐ How to use:

A. Preparation before use

1) Do not open the package until immediately before use.
2) The package must be opened in a sterilized place when using the product.

B. How to operate and use

1) Remove the cleaning solution used during surgery by sucking it with an aspirator.
2) Check if the bleeding on the wound surface has sufficiently stopped during surgery.
3) Unpack the product in a sterilized place, remove the cap of the syringe, and then screw the catheter tube.
4) Sufficiently inject the Guardix-SG solution to ensure that the solution is completely applied to the spinal or thyroid surgery area.

※ To apply the same amount as the experimental product applied to the test group, use 5 ml based on the scale of the syringe and leave 1 ml as the remaining amount. The researcher should document that only 5 ml of the comparator was used, and the sponsor should check and file it. (To ensure accurate use of 5 ml, it is also possible to remove 1 ml of Guardix-SG before surgery and apply all the remaining 5 ml to the subject.)

C. How to store and control after use

1) As the product is disposable, the remaining solution should be discarded after administration

and should not be reused.

D. Remove or disassemble after application : Guardix-SG contains, poloxamer, a raw material with thermo-sensitivity. This substance is metabolized in the liver and excreted through the kidneys, like the experimental product, MegaShield [18,19,20]. And, it is known that calcium ions in alginate gradually spread out of the gel, and alginate is slowly decomposed and discharged through urine [23].

10.4 Study procedure

【Schedule of Clinical Study】

Item	Screening	Application and Evaluation		
Visit	Visit 1	Visit 2	Visit 3	Visit 4
Day	-30~0	Day 0	Day 7±3	Day 42±7
Written consent	●			
Inclusion/exclusion criteria	●			
Demographic survey/physical examination	●			
Vital signs test	●			●
Medical/administering history (1)	●			
Subjects enrollment		●		
Surgery and applying investigational product		●		
Esophagography				●
Evaluating adhesion scores	●		●	●
Urine β-HCG testing (for fertile women)	●			●
Hematological test	●		●	●
Serum biochemical test	●		●	●
Urinalysis	●		●	●
Collecting concomitant drug (2)	●	●	●	●
Collecting adverse event (3)		● (After surgery)	●	●

(1) Collect medical history within 5 years before participating in the clinical trial.

(2) Collect information on drugs that have been administered within 4 weeks of the screening visit or are currently being administered.
(3) Adverse events should be followed up until the symptoms disappear, and if it is difficult for a subject to visit, the follow-up should be performed by phone. (However, if a subject is diagnosed with hypothyroidism or hypoparathyroidism through thyroid function test after thyroidectomy, the resulting adverse events should be followed up until the last visit of the subject for the clinical trial.)

Receive informed consent to participate in the clinical trial from patients who are judged to be in need of total thyroidectomy. After checking the results of hematological test, serum biochemical test and urinalysis, and the inclusion/exclusion criteria, assign a subject identification code only to subjects judged to meet the criteria. In addition, give subjects the randomization number (3 digits) in order according to the randomization table and assign the subjects to the appropriate application group. The randomization code and the subject identification code will be used as the subject number during the clinical trial. The target patient shall have total thyroidectomy and then be subject to the assigned investigational product. When applying the investigational product, apply 5 ml of MegaShield and 5 ml of Guardix-SG for the test group and the control group, respectively, on the thyroidectomy site and the surface of strap muscles. Visit the 1st and 6th weeks after surgery to evaluate the efficacy and safety.

10.5 Contraindication of concurrent use

The use of the following medical devices and medicines is prohibited during the study period.

(1) Anti-adhesion products other than the investigational products for this clinical trial
(2) Antithrombotic/anticoagulant drugs
(3) Systemic corticosteroids : Continuous use of 2 mg/kg (20 mg/day) as prednisolone for 7 days or more, or equivalent dose of a corticosteroid

11. Observation and clinical testing items, and their methods

【Schedule of Clinical Study】

Item	Screening	Application and Evaluation		
Visit	Visit 1	Visit 2	Visit 3	Visit 4
Day	-30~0	Day 0	Day 7±3	Day 42±7
Written consent	●			

Inclusion/exclusion criteria	●			
Demographic survey/physical examination	●			
Vital signs test	●			●
Medical/administering history (1)	●			
Subjects enrollment		●		
Surgery and applying investigational product		●		
Esophagography				●
Evaluating adhesion scores	●		●	●
Urine β-HCG testing (for fertile women)	●			●
Hematological test	●		●	●
Serum biochemical test	●		●	●
Urinalysis	●		●	●
Collecting concomitant drug (2)	●	●	●	●
Collecting adverse event (3)		● (After surgery)	●	●

(1) Collect medical history within 5 years before participating in the clinical trial.

(2) Collect information on drugs that have been administered within 4 weeks of the screening visit or are currently being administered.

(3) Adverse events should be followed up until the symptoms disappear, and if it is difficult for a subject to visit, the follow-up should be performed by phone. (However, if a subject is diagnosed with hypothyroidism or hypoparathyroidism through thyroid function test after thyroidectomy, the resulting adverse events should be followed up until the last visit of the subject for the clinical trial.)

☐ **Efficacy evaluation**

(1) Primary efficacy endpoint : Comparison of esophageal movement

To assess the degree of adhesion, compare the esophageal movement of the test group and the control group through Marshmallow esophagography 6 weeks after surgery.

(2) Secondary efficacy endpoint : Adhesion Scores

Evaluate adhesion scores through surveys at screening, 1 week after surgery, and 6 weeks after surgery, and compare the test group and the control group.

☐ **Safety evaluation**

Investigate all adverse events at every visit after surgery.

11.1. Observation and clinical testing items

1) Visit 1 : Screening (Day -30~0)

Subjects who have agreed to participate in this clinical trial should be screened to ensure that they are eligible to participate in the study.

- Receive informed consent
- Review inclusion/exclusion criteria
- Demographic survey/physical examination : Date of birth, gender, weight, height, etc.
- Vital signs test : Blood pressure, pulse rate
- Medical/administering history : Diabetes mellitus, hypertension, stroke, ischemic heart disease, malignant tumor, acute infection, allergy, and whether taken bisphosphonate, etc.
- Evaluate adhesion scores
- Urine β-HCG testing (only for women with childbearing age)
- Hematological test : RBC, Hemoglobin, Hematocrit, Platelet, WBCs, Neutrophil, Lymphocyte, Monocyte, Eosinophil, Basophil, Blood coagulating time (PT/APTT)
- Serum biochemical test : sodium, Potassium, Chloride, Creatinine, BUN, ALT, AST,ALP, Total Bilirubin, Albumin, Total Protein, Total Cholesterol, Glucose
- Urinalysis : pH, Specific gravity, WBC, Protein, Bilirubin, Glucose, Urobilinogen, Ketone, Nitrite, Blood
- Collect concomitant drugs

2) Visit 2 : Day 0

- Perform a surgery and apply an investigational product
- Enroll subjects
- Collect concomitant drugs
- Collect adverse events : After surgery

3) Visit 3 : Day 7±3

- Evaluate adhesion scores
- Hematological test : RBC, Hemoglobin, Hematocrit, Platelet, WBCs, Neutrophil, Lymphocyte, Monocyte, Eosinophil, Basophil, Blood coagulating time (PT/APTT)
- Serum biochemical test : sodium, Potassium, Chloride, Creatinine, BUN, ALT, AST,ALP, Total Bilirubin, Albumin, Total Protein, Total Cholesterol, Glucose
- Urinalysis : pH, Specific gravity, WBC, Protein, Bilirubin, Glucose, Urobilinogen, Ketone, Nitrite,

Blood

- Collect concomitant drugs
- Collect adverse events

4) Visit 4 : Day 42±7

- Check vital signs
- Esophagography
- Evaluate adhesion scores
- Urine β-HCG testing (only for women with childbearing age)
- Hematological test : RBC, Hemoglobin, Hematocrit, Platelet, WBCs, Neutrophil, Lymphocyte, Monocyte, Eosinophil, Basophil, Blood coagulating time (PT/APTT)
- Serum biochemical test : sodium, Potassium, Chloride, Creatinine, BUN, ALT, AST,ALP, Total Bilirubin, Albumin, Total Protein, Total Cholesterol, Glucose
- Urinalysis : pH, Specific gravity, WBC, Protein, Bilirubin, Glucose, Urobilinogen, Ketone, Nitrite, Blood
- Collect concomitant drugs
- Collect adverse events

11.2 Observation method

11.2.1 Signing informed consent

Prior to conducting this clinical trial, the investigator shall explain the contents of the 'Informed Consent Form' to the subjects or their legal representative, etc., only for subjects judged to meet the inclusion criteria, and ensure that the subjects or their legal representative, etc. understand the contents well. And then, the investigator shall obtain the written consent to participate in the clinical trial at their will. In addition, the date of signing the consent shall be recorded in the Case Report Form.

11.2.2 Demographic and medical history survey

Prior to conducting the clinical trial, check the subject's demographic information and medical/administering history, including the followings, through interviews, medical charts, and questions, and record them in the Case Report Form.

- Demographic survey : Date of birth, gender
- Physical examination and vital signs: Weight, height, blood pressure, pulse rate
- Medical history : Medical conditions that can affect surgery or anesthesia, such as diabetes mellitus, hypertension, stroke, ischemic heart disease, osteoarthritis, gout, hyperuricemia, sleep apnea syndrome, gallbladder disease, back pain or peripheral blood disorder

- Testing : Hematological test/serum biochemical test/urinalysis etc.
- Others : Whether taking drugs, smoking, drinking, pregnancy status and plan

11.2.3 Evaluation on subject eligibility

Evaluate whether the subject meets the inclusion and exclusion criteria through demographic survey, medical history, past clinical records (medical charts), and questionnaires.

11.2.4 Assignment of subject identification code

Assign a subject identification code sequentially only to subjects who agree to participate in the clinical trial and meet the inclusion/exclusion criteria according to the following method, and write the "Screening/Enrollment log." The subject identification code is used as an identification code along with the randomization code and subject initials.

- Institution: Severance Hospital (S01), Kangbuk Samsung Medical Center (S02), Seoul St. Mary's Hospital (S03)
- Subject Identification Code: Institution code-Enrolled order

Ex) Subjects enrolled in Severance Hospital: S01-001, S01-002...

Subjects enrolled in Kangbuk Samsung Medical Center: S02-001, S02-002...

Subjects enrolled in Seoul St. Mary's Hospital: S03-001, S03-002

11.3 Randomization method

11.3.1 Randomization

For randomization, make sure that subjects are assigned to the test group and the control group at a ratio of 1:1 by the block randomization method. It will be generated using SAS by a statistical expert who is not directly related to the institution. For blinding, before the study is terminated, only a minimum number of personnel who are not directly related to the institution should be able to access to the randomization table and code. Subsequently, the institution shall finally assign a randomization number (3-digit number) to the subjects who meet the inclusion/exclusion criteria through screening. This randomization code, together with the subject identification code specified in 11.2.4, will be used as the subject identification code during the clinical study.

Ex.: Randomization code **R 1 0 1**

2nd, 3rd digits – sequentially

1st digit – institution code (Severance 1, Kangbuk Samsung 2, Seoul St. Mary's 3)

11.3.2 Unblinding

A. Method of unblinding during the clinical trial

Use an unblinding sealed bag for unblinding during the clinical trial. The bag should be brown so that the contents are not shown when shining it in, and there should not be a hole to show the contents. And the signature should be on the sealed part so that it can be shown whether the unblinding sealed bag has been opened or not.

B. Situations requiring unblinding during the clinical trial

① When a serious adverse event occurs, if information how to deal with it is required, unblinding can be performed.

② In the event of a Suspected Unexpected Serious Adverse Reaction (SUSAR), unblinding must be performed.

C. Procedure of unblinding during the clinical trial

① The unblinding sealed bag should be delivered to the PI prior to initiating the clinical trial and should not be accessed by anyone other than the person concerned. In addition, it should be stored close to the PI.

② When unblinding, the information should be delivered directly to the person who performs medical treatment which requires the information, and no other researchers or related persons should be informed of randomization except where necessary.

③ In the event of a situation requiring unblinding, write the information on the subject and researcher, date of unblinding, reason for unblinding, etc. in 'Document on Unblinding', the source document, and CRF. Also, the signature of the person who performed the unblinding, the date and time of unblinding, etc. should be recorded on the outside of the bag.

④ Deliver the written 'Document on Unblinding' to the sponsor by e-mail or fax.

⑤ All unblinding sealed bags should be collected by the PI at the end of the clinical trial.

D. Unblinding after the completion of the clinical trial

① Blinding should be maintained before the database is officially locked for statistical analysis.

② Once the analysis set is determined after the blind review, the person in charge of producing (or labeling) the investigational product shall deliver the product code to the person in charge of creating the randomization code, and the person in charge of creating the randomization code shall deliver the randomization code, containing the actual group instead of the product code, to the person in charge of analysis.

11.4 Independent evaluator’s evaluation

The evaluator of the institution who evaluates the esophagography (primary endpoint) and adhesion scores shall conduct and evaluate surveys of esophagography and adhesion scores while being blinded. And the independent evaluator should be blinded to the treatment group assignment of the subjects so that the independent evaluator can make an objective judgment.

11.5 Primary endpoint: Comparison of esophageal movement using Marshmallow esophagography

After 6 weeks of thyroidectomy and application of the investigational product, ask the patient to take a prone position. Instruct the patient to swallow a mass of 20 mm diameter marshmallow with liquid barium sulfate (Solotop Sol. 140), then observe the marshmallow's passage through the esophagus by fluoroscopy [24,25,26].

First, measure the esophageal passage time of the marshmallow in the prone position, and classify it into normal and abnormal based on 30 seconds. When the marshmallow gets stuck in a part of the esophagus and does not move for more than 30 seconds, it is defined as an impaction. If an impaction has occurred in the prone position, ask the patient to change the posture to standing position and swallow his/her saliva, then observe the movement of the marshmallow in the esophagus. With this test, evaluate the marshmallow's passage pattern in the esophagus by dividing them into 4 grades as shown in the following table [24].

Score	Definition	Esophageal passage time of marshmallow
3	Normal	Passed through the esophagus within 30 seconds in the prone position
2	Minor	Passage time exceeds 30 seconds in the prone position; and passed within 30 seconds in the standing position
1	Moderate	Impacted in the prone position; and passage time exceeds 30 seconds in the standing position
0	Severe	Impacted in both the prone and standing positions

11.6 Secondary endpoint: Adhesion Scores

Evaluate adhesion scores through surveys at screening, 1 week and 6 weeks after thyroidectomy and application of the investigational product. Score each clinical symptom for all questionnaires on a scale of 0 to 10. The higher the score, the more severe the adhesion [24,27].

Evaluation item	
Subjective discomfort of patient	1. Do you have trouble swallowing your saliva?<br>2. Do you have difficulty swallowing water?<br>3. Do you have any difficulty in swallowing hard foods?<br>4. Are your neck wrinkles abnormal in your own opinion?
Objective evaluation of investigator	5. Are the wrinkles on the neck symmetrical and natural when the patient is at rest?<br>6. Are the wrinkles on the neck symmetrical and natural when the patient is at neck extension?

	7. What is the degree of inflammatory response or scar formation based on the surgical findings?

What is the neck extension? : A condition in which the subject's jaw is usually directed upward at 30 to 45 degrees from the horizontal so as not to be uncomfortable when the subject extends his/her neck

11.7 Safety evaluation

Check whether there is any adverse event through vital signs, hematological test, serum biochemical test and urinalysis immediately after surgery, at 1 week and 6 weeks after surgery.

12. Predicted side effects and precautions for use

12.1 Predicted side effects

1) Side effects associated with general surgery : Abscess, cellulitis, wound, dermal necrosis, swelling, hematoma, cardiovascular disease, hypertension, ischemia, thrombus, embolism, hemorrhage, complications related to anesthesia, pulmonary complications, nerve/muscle damages, etc.
2) Side effects associated with the use of anti-adhesion product : Infection, allergic reaction, pain, and inflammatory reaction
3) Side effects associated with total thyroidectomy : Hypoparathyroidism, recurrent laryngeal nerve injury, hemorrhage, infection, superior laryngeal nerve paralysis, dyspnea, etc.

   In the event of a predicted side effect, record the name and extent of the side effect as an adverse event in the Case Report Form according to the following criteria for mild, moderate and severe.

▪ Mild : A degree to which a subject can easily endure, without interfered with his/her normal daily life (or function) and with causing minimal discomfort

▪ Moderate : A degree to which it causes inconvenience to the extent that it significantly hinders the normal daily life (or function) of a subject

▪ Severe : A degree to which the normal daily life (or function) of a subject is impossible; a degree to which a subject is unable to continuously participate in the study; or a degree to which a subject needs to be treated or hospitalized

12.2 Precautions for use of experimental product

12.2.1 General precautions

1) Read all instructions and precautions for use before using this product. Failure to properly follow the instructions and precautions for use may result in the product not functioning properly or infection or side effects.
2) MegaShield is a surgical product, shall be used by specialized medical personnel and shall not be used for any purpose other than its intended use.
3) Since this product is sterile, it should be maintained in a sterilized state during the procedure, and expired products should not be used.
4) Before use, the contents of the product should be checked. If any signs of damage or moisture appear in the packaging or containers, do not use the product.
5) Before the MegaShield solution is injected into the surgical site, the surgeon should remove any remaining liquid, such as cleaning solution, by sucking it with an aspirator.
6) If a patient is found to have side effects, follow the "15. Evaluation Standard of Safety Including Side Effects, Its Evaluation Method and Report Method" in this protocol. In addition, hospitals and physicians should take the necessary medical measures for the patient.
7) The validity period is one year from the date of manufacture.

12.2.2 Precautions for adverse events which may occur as a result of using the medical device, fatal side effects due to negligence in use, and accidents

1) If any damage to the package is found prior to use, the product shall not be used, and the damaged product shall be returned or exchanged by contacting L&C Bio Inc.
2) If a product opened prior to transplantation to a patient has been left in a contaminated and non-sterile condition, it should not be used for transplantation and should be returned by contacting L&C Bio Inc. or disposed of appropriately as medical waste.
3) Before using this product for transplantation, it is essential that the patient to be transplanted has an allergic reaction to any reagent or solution used in the manufacture of this product.
4) This product shall be used within the expiration date indicated on the label, and products that have been expired shall be returned or exchanged by contacting L&C Bio Inc.
5) This product shall be stored in a cool, dry place, avoiding direct sunlight, in accordance with the recommended storage conditions.

12.2.3. Use for pregnant women, lactating women, women of childbearing age, newborns, infants, children, and the elderly

This product should not be used if a patient to be transplanted has an allergic reaction to any reagent or solution used in the manufacture of this product, prior to use for transplantation. This product should not be used if a patient, in particular, with reduced immunity has the potential to cause side effects.

12.2.4. Precautions on application

1) This product shall be stored and used at room temperature.

2) This product is a gamma-sterilized product, it should not be used by re-sterilization, and once opened, it should not be re-sterilized for reuse.

3) Each individually packaged MegaShield should be used only for one patient.

4) Since this product is disposable, the remaining solution should be discarded after use and should not be reused.

12.2.5. Matters necessary to prevent safety accidents

1) This product is produced through the GMP process certified by the Ministry of Food and Drug Safety. Therefore, it is recommended that this product be used in appropriate environments (clean room or operating room) and facilities similar to the manufacturing environment when using this product.

2) Be careful not to contaminate the affected area or the treatment area when using this product.

12.3 Precautions for use of comparator

12.3.1 General precautions

1) Since this product is sterile, it should be maintained in a sterilized state during the procedure, and expired products should not be used.

2) Before use, the contents of the product should be checked. If any signs of damage or moisture appear in the packaging or containers, do not use the product.

3) Before the GUARDIX-SG solution is applied to the surgical site, the surgeon should remove any remaining liquid by sucking it with an aspirator.

12.3.2 Precautions for handling

1) GUARDIX-SG solution should be stored and used at room temperature.
2) GUARDIX-SG solution can be used even when warmed to body temperature and becomes a gel.
3) Sterilized GUARDIX-SG is provided and shall not be used after re-sterilization.
4) Since this product is disposable, the remaining solution should be discarded after use and should not be reused.

12.3.3 Contraindications

1) GUARDIX-SG solution should not be applied to a patient with infected or contaminated surgical site.
2) GUARDIX-SG solution should not be applied to a patient who is hypersensitive to its ingredients.

3) When GUARDIX-SG solution is used in combination with other anti-adhesion products, absorbable hemostatic agents, or other drugs, its safety and efficacy have not been verified in animal testing.
4) The use of GUARDIX-SG solution is not recommended during pregnancy.
5) It is recommended to avoid pregnancy until the end of the first menstrual cycle after use of GUARDIX-SG solution.

12.3.4 Predicted side effects
1) Infection : Pyrexia, leukocytosis
2) Allergic reaction : Generalized rash, dyspnea, edema
3) Pain
4) Inflammation

13 Discontinuation and drop out

13.1 Criteria for discontinuation

1) Where the principal investigator determines that the situation observed during the clinical trial is unreasonable for continuing the clinical trial
2) Where the clinical trial is to be discontinued due to the safety of the investigational product, etc.
3) Where the study is temporarily discontinued to deal with an adverse event that has occurred
4) Where the used medical device is removed and the study is discontinued to deal with an adverse event that has occurred
5) Where the study is discontinued due to the occurrence of a serious adverse event/adverse device reaction

13.2 Management of discontinuation

1) If the clinical trial is discontinued, the principal investigator shall collect and deliver the Case Report Form, the clinical trial progress status and results of the subject of which have progressed to the point where the study was discontinued to Sponsor. All study-related data (Case Report Form and the investigational product) shall be returned to the sponsor. Subjects whose the clinical trial has been discontinued shall be included in statistical processing for safety and efficacy evaluation.
2) If the clinical trial is terminated early or temporarily discontinued, the principal investigator shall immediately inform the subjects and ensure that appropriate measures and follow-up are carried out. And the principal investigator shall notify the Institutional Review Board of that in writing.

13.3 Criteria for drop out

1) Where a subject or his/her legal representative requests to stop participating in the clinical trial
2) Where surgery, drugs, or other medical devices that may affect the safety or efficacy have been used in combination
3) Where serious side effects occur
4) Where the treatment method has not been performed properly
5) Where a subject fails to comply with the requirements presented in the consent form, thus affecting the evaluation
6) Where continuous observation is not possible due to the absence of a subject
7) Other cases where the investigator determines that there is a problem in conducting the clinical trial

13.4 Management of drop out

1) If a subject is dropped out, record and keep the reason for the drop out and the related data to the clinical trial progressed before the drop out.
2) If a subject failed to visit during the study, check the subject's health condition and clarify the reason.
3) The subject dropped out shall be included in statistical processing when evaluating the safety and efficacy.

14 Evaluation Standard of Efficacy, Its Evaluation Method and Analysis Method (by statistical analysis method)

The evaluation standard, evaluation method and analysis method of efficacy shall be based on the clinical study procedures published in the Korean Journal of Endocrine Surgery in 2009 [24].

14.1 Primary efficacy endpoint

For the primary endpoint of this clinical trial, score the esophageal movement scores evaluated by marshmallow esophagography at 6 weeks after surgery into four grades (0, 1, 2, and 3). The primary endpoint is evaluated by providing the frequency, proportion, mean, standard deviation, median, minimum and maximum of the esophageal movement scores and by using the one-sided 97.5% confidence interval for the difference in the mean score of the 'esophageal movement' between the two groups (test group - control group) to determine whether the effect of the new treatment is non-inferior to the existing treatment. That is, if the lower limit of the one-sided 97.5% confidence

interval is greater than -0.1, which is the limits of non-inferiority (-δ), it is judged that 'the test group is non-inferior to the control group.'

※Marshmallow esophagography

First, measure the esophageal passage time of the marshmallow in the prone position, and classify it into normal and abnormal based on 30 seconds. When the marshmallow gets stuck in a part of the esophagus and does not move for more than 30 seconds, it is defined as an impaction. If an impaction has occurred in the prone position, ask the patient to change the posture to standing position and swallow his/her saliva, then observe the movement of the marshmallow in the esophagus. With this test, evaluate the marshmallow's passage pattern in the esophagus by dividing them into 4 grades as shown in the following table.

Score	Definition	Esophageal passage time of marshmallow
3	Normal	Passed through the esophagus within 30 seconds in the prone position
2	Minor	Passage time exceeds 30 seconds in the prone position; and passed within 30 seconds in the standing position
1	Moderate	Impacted in the prone position; and passage time exceeds 30 seconds in the standing position
0	Severe	Impacted in both the prone and standing positions

14.2 Secondary efficacy endpoint

The secondary endpoint is adhesion scores.

Evaluate adhesion scores through the following surveys at screening, 1 week and 6 weeks after thyroidectomy and application of the investigational product.

The secondary endpoint is evaluated by providing descriptive statistics (mean, standard deviation, median, minimum and maximum) by time point and group, and by providing descriptive statistics of the mean changes, compared to those at the screening, for each time point and each group after surgery. For the mean and mean variance of the intergroup at each time point, the unpaired t-test or Wilcoxon ranksum test is applied depending on whether the normality assumption is satisfied (Shapiro-Wilk test), and for the mean variance of the intragroup, the paired t-test or Wilcoxon signed rank test is applied. The significance level for each statistical analysis is $p<0.05$.

Evaluate adhesion scores through surveys at screening, 1 week and 6 weeks after thyroidectomy and application of the investigational product. Score each clinical symptom for all questionnaires on a scale of 0 to 10. The higher the score, the more severe the adhesion [24,27].

Subjective discomfort of patient	1. Do you have trouble swallowing your saliva?<br>2. Do you have difficulty swallowing water?<br>3. Do you have any difficulty in swallowing hard foods?<br>4. Are your neck wrinkles abnormal in your own opinion?
Objective evaluation of investigator	5. Are the wrinkles on the neck symmetrical and natural when the patient is at rest?<br>6. Are the wrinkles on the neck symmetrical and natural when the patient is at neck extension?<br>7. What is the degree of inflammatory response or scar formation based on the surgical findings?

What is the neck extension? : A condition in which the subject's jaw is usually directed upward at 30 to 45 degrees from the horizontal so as not to be uncomfortable when the subject extends his/her neck

14.3 Analysis set

In the efficacy analysis of this clinical trial, the main analysis is the mITT set, and the PP set is additionally analyzed. The analysis for the safety is ITT set.

- ITT Set: An analysis set targeting for subjects who have received treatment at least once with an investigational product, among all subjects enrolled in this clinical trial
- Modified ITT Set: An analysis targeting for subjects who have received treatment at least once with an investigational product and participated in the primary efficacy endpoint evaluation, among all subjects enrolled in this clinical trial
- PP set: An analysis targeting for subjects who completed the study in accordance with this protocol without any significant violation of the protocol among ITT set

The exclusion criteria from the PP set because it is considered a serious violation of the protocol is as follows.

1) Violation of inclusion/exclusion criteria
2) Administration of drugs prohibited from concurrent use during the clinical trial
3) Other cases where it can be considered a serious violation of the protocol

14.4 Treatment of missing data

If there is a missing data in the analysis of the Modified ITT set for efficacy evaluation, the primary endpoint does not require a separate calibration according to the definition of the main analysis set, and the secondary endpoint is analyzed within the data observed at each time point and alternative processing for missing value is not planned. However, if the missing data is more than

10% of the analysis data during the secondary efficacy evaluation, apply the Repeated Mixed Model considering the missing data and present the results of additional analysis.

14.5 Analysis of demographic and basic data

Examine demographic information on the mITT set. In the demographic and basic data of the subjects, continuous data (age, weight, height, blood pressure and pulse rate) shall be presented as descriptive statistics (the number of subjects observed, mean, standard deviation, median, minimum and maximum), and categorical data (gender, major medical history, drug history, smoking and drinking) as frequency and percentage. When comparing the demographic and basic data between the two groups, continuous data (age, weight, height, pulse rate) shall be compared by unpaired t-test or Wilcoxon ranksum test depending on whether the normality assumption is satisfied (Shapiro-Wilk test), and categorical data (gender, major medical history, drug history, smoking, drinking) shall be compared by Chi-square test (Fisher's exact test if the cell of which the expected frequency is not more than 5 exceeds 20% of the total).

15 Evaluation Standard of Safety Including Side Effects, Its Evaluation Method and Report Method

15.1 Definition of adverse event

"Adverse Event (AE)" means any unintended sign (including abnormalities from laboratory test results, etc.), symptom or disease that occurs to a subject during the clinical trial, and is not necessarily causally related to the investigational product.
"Adverse Device Effect (ADE)" refers to any harmful and unintended reaction caused by the investigational product, and means an adverse event that cannot be denied a causal relationship with the investigational product.
"Unexpected Adverse Device Effect" refers to a difference in the aspect or degree of harm from that of the Adverse Device Effect based on available medical device-related information such as Investigator's Brochure or the attached documents of the medical device.
The followings are excluded from adverse events.

1) Medical or surgical treatment (ex: surgery, endoscopy, tooth extraction, appendectomy) ; The condition that caused this treatment is an adverse event.
2) Where any existing disease that was already present or found at the start of the clinical trial did not worsen after the treatment of the investigational product

3) Hospitalization for surgery to treat an existing disease that has not worsened scheduled before participating in the clinical trial
4) Hospitalization for medical examination
5) Optional hospitalization for cosmetic surgery

15.2 Definition of serious adverse event/adverse device effect (Serious AE/ADE)

Serious adverse event/adverse device effect means a case that falls under any of the followings among adverse events or adverse device effects that occurred during the clinical trial.
1) Where death or life-threatening danger occurs
2) Where it is required to be hospitalized or extend the period of hospitalization
3) Where permanent, serious disability or serious hypofunction was resulted in
4) Where malformation or abnormality occurred in the fetus

15.3 Evaluation of adverse event

Adverse events shall be evaluated by the principal investigator or the sub-investigator.

15.3.1 Severity evaluation
If an adverse event occurs, it should be reported according to the following criteria for severity.
· Mild : A degree to which a subject can easily endure, without interfered with his/her normal daily life (or function) and with causing minimal discomfort
· Moderate : A degree to which it causes inconvenience to the extent that it significantly hinders the normal daily life (or function) of a subject
· Severe : A degree to which the normal daily life (or function) of a subject is impossible; a degree to which a subject is unable to continuously participate in the study; or a degree to which a subject needs to be treated or hospitalized

15.3.2 Assessment of causal relationship with investigational product
In the event of an adverse event, the investigator shall evaluate whether it has the relevance with the investigational product based on the following criteria. And, the investigator's opinion should be described.
· Definitely related
- The temporal sequence between the use of the investigational product and the onset of the adverse event is reasonable.
- It is most likely that the adverse event has occurred due to the use of the investigational product than any other reason.

- When stopping using the investigational product, the adverse event disappears.
- Reusing the investigational product (only if re-use is allowed), the results are positive.
- The adverse event is consistent with previously known information about the investigational product or medical devices of the same family.

· Probably related
- There is evidence of using the investigational product.
- The temporal sequence between the use of the investigational product and the onset of the adverse event is reasonable.
- It is more likely that the adverse event has occurred due to the use of the investigational product than any other reason.
- When stopping using the investigational product, the adverse event disappears.

· Possibly related
- There is evidence of using the investigational product.
- The temporal sequence between the use of the investigational product and the onset of the adverse event is reasonable.
- It is judged that the adverse event is due to the use of the investigational product at the same level as other possible causes.
- When stopping using the investigational product (if performed), the adverse event disappears.

· Probably not related
- There is evidence of using the investigational product.
- There is a more probable cause of the adverse event.
- When discontinuing the use of the investigational product, the results are negative or ambiguous (if performed).
- Reusing the investigational product (only if re-use is allowed), the results are negative or ambiguous.

· Not related
- The investigational product was not used.
- The temporal sequence between the use of the investigational product and the onset of the adverse event is not reasonable.
- There are other obvious causes of the adverse event.

· Unknown
- Information is insufficient or conflicting and cannot be judged. And the information cannot be supplemented or verified.

15.4 Evaluation standard of safety

In this clinical trial, all undesirable medical findings that develop symptoms that were not observed

before the application of the investigational product are classified as adverse events. The predicted side effects are also classified as adverse events, and the severity of adverse event is classified as mild, moderate, and severe.

15.4.1 Predicted side effect

1) Side effects associated with general surgery : Abscess, cellulitis, wound, dermal necrosis, swelling, hematoma, cardiovascular disease, hypertension, ischemia, thrombus, embolism, hemorrhage, complications related to anesthesia, pulmonary complications, nerve/muscle damages, etc.
2) Side effects associated with the use of anti-adhesion product : Infection, allergic reaction, pain, and inflammatory reaction
3) Side effects associated with total thyroidectomy : Hypoparathyroidism, recurrent laryngeal nerve injury, hemorrhage, infection, superior laryngeal nerve paralysis, dyspnea, etc.

15.5 Safety evaluation method (statistical analysis method)

The analysis for the safety is ITT set. For all side effects reported during the study, determine the number of patients with the side effects, the rate and the number of occurrence by group. Analyze the incidence of adverse events between the test and control groups using the chi-square test (or Fisher's exact test).

Present descriptive statistics for adverse events by observation point and group. Among the results of hematological test, serum biochemical test and urinalysis, continuous data shall be presented as descriptive statistics (the number of subjects observed, mean, standard deviation, median, minimum and maximum), and categorical data as frequency and percentage. Since the occurrence of somatic signs is categorical data, it shall be presented as frequency and percentage. If necessary, these data can be compared and evaluated by applying statistical analysis (chi-square test, Fisher's exact test, unpaired t-test, paired t-test, etc.). The significance level of each statistical analysis is $p<0.05$.

15.6 Monitoring and reporting system of adverse event

15.6.1 Guidance on adverse events

The principal investigator shall provide information to the sub-investigator and subjects or legal representatives about any adverse events that may arise after use of the investigational product, and instruct them to report any adverse event that occurs after use.

15.6.2 Documentation of adverse events

All adverse events that occurred during the clinical trial period (after treatment with the investigational product) should be recorded in the subject's medical record and Adverse Event

column on the Case Report Form, even if they are unrelated to the use of the investigational product. All serious adverse events that occurred during the clinical trial period (after treatment with the investigational product) should be recorded in the subject's medical record and Adverse Event column on the Case Report Form and Serious Adverse Event Form.

Each serious adverse event shall be recorded in each Serious Adverse Event Form. However, if there are a number of serious adverse events that are temporarily and/or clinically relevant in the initial report, they may be written and reported in the same Serious Adverse Event Form. In such cases, individual Serious Adverse Event Report shall be submitted after follow-up information has been obtained and final assessment/diagnosis has been made.

The investigator should try to make diagnoses of adverse events based on signs, symptoms, and/or other clinical information. In this case, the diagnosis should be recorded, not the signs or symptoms of each adverse event.

If a clinically significant laboratory abnormal findings or other abnormal evaluation is an adverse event, enter it in the Adverse Event column of the Case Report Form. In addition, if an adverse event meets the criteria for the serious adverse event, it should be entered on the Serious Adverse Event Form. If the diagnosis is identified, the diagnosis should be recorded in both the Adverse Event column of the Case Report Form and the Serious Adverse Event Form rather than clinically significant laboratory abnormal findings. If the diagnosis is not identified, record the laboratory abnormal findings.

The investigator shall evaluate the severity of adverse events in three stages, as follows.

Severity	Description
Mild	A degree to which a subject can easily endure, without interfered with his/her normal daily life (or function) and with causing minimal discomfort
Moderate	A degree to which it causes inconvenience to the extent that it significantly hinders the normal daily life (or function) of a subject
Severe	A degree to which the normal daily life (or function) of a subject is impossible; a degree to which a subject is unable to continuously participate in the study; or a degree to which a subject needs to be treated or hospitalized

15.6.3 Guidance on serious adverse events/adverse device reactions

The investigator shall report all serious adverse events/adverse device reactions that occurred during the clinical trial to the sponsor by fax or e-mail within 24 hours, regardless of whether they are

related to the use of the investigational product.
The following table summarizes the deadlines for reporting serious adverse events and the required documents.

Report of serious adverse event

	Initial report	Follow-up report
Deadline	24 hours [a]	As soon as additional information is obtained [b]
Document	■ Completely fill out the Serious Adverse Event Form of the sponsor<br>■ All diagnostic test results<br>■ Case Report Form for adverse events<br>■ Case Report Form for medical history/demographic data/concomitant drugs<br>■ In the event of death, an autopsy report and a death certificate shall be added.	■ Serious Adverse Event Form of the sponsor for "Follow-up" report<br>■ A case that the submitted Care Report Form has been updated<br>■ Final and detailed summary of adverse events prepared by the investigator<br>■ Summary of discharge, if necessary<br>■ All related diagnostic test results/reports including pathological report, summary of discharge, post-mortem, histopathological results, etc.

a **Within 24 hours** after the institution finds of an adverse event
b **Within 24 hours** after the institution obtains additional information

In addition, record events deemed serious by the investigator or that suggest significant risks, contraindications, side effects, or precautions that may be associated with the use of the investigational product as serious adverse events/adverse device reactions.
An additional report containing details in the deadline specified in the protocol shall be documented.
In such cases, the subject identification code shall be used instead of the subject's name, resident registration number and address, and if there are relevant guidelines for reporting serious adverse events/adverse device reactions, the principal investigator shall follow them.
The principal investigator shall report adverse events/adverse device reactions specified in the protocol or abnormalities in laboratory test values which are critical for safety evaluation to the sponsor according to the deadline and reporting method specified in the protocol.
When reporting a death case, the principal investigator shall provide additional information to the sponsor and the IRB, such as an autopsy report (only if an autopsy has been performed) and a death certificate.
In the final report, the following information should be provided, if possible.

- Information on the occurrence time, degree, treatment, progress, causality with the investigational product should be recorded in the Case Report Form.

15.6.4 Measures to be taken when an adverse event occurs
During this clinical trial, the principal investigator and sub-investigator shall make every effort to ensure the safety of the subjects, and in the event of an unexpected serious adverse event/adverse device reaction, they shall take prompt and appropriate measures to minimize the adverse events.

The responsibilities of each person in charge in the event of a 'serious adverse event/adverse device reaction' during the clinical trial are as follows.
- Responsibility of principal investigator :
The principal investigator shall immediately report to the IRB and the sponsor and stop all or part of the clinical trial with the investigational product until further instructions are given when a serious adverse event/adverse device reaction occurs during the clinical trial.

- Responsibility of sub-investigator :
The sub-investigator shall immediately report to the principal investigator and the sponsor when a serious adverse event/adverse device reaction occurs during the clinical trial.

- Responsibility of institutional review board :
The institutional review board shall take necessary measures such as ordering the discontinuing all or part of the clinical trial to the principal investigator when a serious adverse event/adverse device reaction is reported.

- Responsibility of sponsor :
When the sponsor receives a report of a serious adverse event/adverse device reaction from the principal investigator or sub-investigator, the sponsor shall immediately submit the report to the Minister of Food and Drug Safety with the report submitted by the principal investigator or sub-investigator. If the clinical trial is conducted in multiple institutions, the sponsor shall immediately report to the corresponding institution. In addition, if the principal investigator did not report to the IRB, or if there is a need to change the reported data, the sponsor shall report this to the IRB.

The reporting deadline is as follows.
· If it causes death or is life-threatening : Within 7 days from the date the sponsor was reported or informed. In such cases, detailed information shall be additionally reported within 8 days of the date of initial reporting.

· In case of other serious and unexpected adverse device reaction : Within 15 days from the date the sponsor was reported or informed

15.6.5 Follow-up observation of adverse events

The investigator should follow up the subject with an adverse event until the symptom disappear from the subject and his/her condition becomes stable, and should submit a report on the subsequent progress of the adverse event.

If there is additional information on an adverse device reaction that has already been reported, the sponsor should report it until the adverse device reaction is terminated.

16 Informed consent form

This clinical trial is a study conducted for research purposes.

This manual and consent form is to provide you with information about this study. Please read the information below carefully and discuss it with your acquaintances. If you have any questions, feel free to contact your doctor or staff to get an answer.

1) Purpose and background of the clinical trial

Your doctor has determined that you need to receive total thyroidectomy. To prevent adhesion in the surgical area, the hospital would like to conduct a study to verify the anti-adhesion effectiveness of MegaShield by applying either Guardix-SG, a product already proven to be anti-adhesion, or MegaShield, which is being developed as a new anti-adhesion product, to the surgical site.

Anti-adhesion product is a medical device to prevent adhesion caused by postoperative side effects. Scarring and adhesion after thyroidectomy cause problems such as hypofunction of the thyroid gland and surrounding nerves, and cause side effects such as paralysis of glottis, neck or chest pain, and dysphagia. In severe cases, secondary surgery may be required to remove adhesion. Therefore, anti-adhesion products are used to minimize these side effects.

MegaShield, an anti-adhesion product to be used in this clinical trial, is a milky white viscous solution in which a thermo-sensitive polymer is mixed with human-derived acellular dermal matrix powder and cross-linked hyaluronic acid. It remains in a fluid solution state before application, but when applied to the wound surface after surgery, it turns into a gel state, acting as a barrier in areas where adhesion is expected to occur. It has been reported that the acellular allogenic dermal matrix, one of the raw materials of MegaShield, has the effect of anti-adhesion in many surgical treatments, and studies have shown that transplanting acellular allogenic dermal matrix after thyroidectomy has an anti-adhesion effect. In addition, an animal test on rats demonstrated that MegaShield has sufficient functionality as an anti-adhesion. MegaShield was safely used without side effects in animal testing. In addition, there are test results that it is safe as it is decomposed in and excreted

from the body after being applied to the surgical site. This safety can be further supported by the fact that MegaShield is formulated with ingredients used in already approved products. However, MegaShield has not yet been approved for anti-adhesion effects after total thyroidectomy. This clinical trial aims to evaluate the efficacy and safety of MegaShield in humans, which is an untested clinical trial.

And, Guardix-SG, an anti-adhesion product to be used as the comparator, consists of poloxamer and sodium alginate, and is a product with proven anti-adhesion effect in patients who have undergone thyroidectomy through a clinical trial.

2) Various tests and procedures you will undergo in this clinical trial

You will be treated with one anti-adhesion product of either MegaShield or Guardix-SG to the surgical site after thyroidectomy.

This clinical trial will be conducted as follows.

- Study period : From the date of approval by the IRB to December 31, 2020
- Expected study duration of participation of subjects : 6 weeks (Up to 2 months)
- Expected number of subjects : 140 people in total (total number of subjects including 3 domestic institutions)
- Whether or not the medical devices are marketed :

  - MegaShield (Test Group) - Not marketed
  - Guardix-SG (Control Group) - On the market

- The schedule of the clinical trial is as follows.

Item	Screening	Application and Evaluation		
Visit	Visit 1	Visit 2	Visit 3	Visit 4
Day	-30~0	Day 0	Day 7±3	Day 42±7
Written consent	●			

Inclusion/exclusion criteria	●			
Demographic survey/physical examination	●			
Vital signs test	●			●
Medical/administering history (1)	●			
Subjects enrollment		●		
Surgery and applying investigational product		●		
Esophagography				●
Evaluating adhesion scores	●		●	●
Urine β-HCG testing (for fertile women)	●			●
Hematological test	●		●	●
Serum biochemical test	●		●	●
Urinalysis	●		●	●
Collecting concomitant drug (2)	●	●	●	●
Collecting adverse event (3)		● (After surgery)	●	●

(1) Collect medical history within 5 years before participating in the clinical trial.

(2) Collect information on drugs that have been administered within 4 weeks of the screening visit or are currently being administered.

(3) Adverse events should be followed up until the symptoms disappear, and if it is difficult for a subject to visit, the follow-up should be performed by phone. (However, if a subject is diagnosed with hypothyroidism or hypoparathyroidism through thyroid function test after thyroidectomy, the resulting adverse events should be followed up until the last visit of the subject for the clinical trial.)

The tests and procedures you will undergo in this clinical trial are as follows.

- Procedures commonly applied to both the test group and the control group : Subjects will be assigned to either the control group treated with Guardix-SG or the test group treated with MegaShield through randomization if it is determined that they meet the inclusion criteria after undergoing a basic examination before surgery. Randomization means that you are assigned to the test group or the control group with the probability of flipping a coin, and you do not know which product applies to you. On the day of surgery, the anti-adhesion product, MegaShield or Guardix-

SG, will be applied, and after 1 and 6 weeks, you will be examined for discomfort such as paresthesia or pain at the surgical site, abnormal scars, etc. through a simple interview. Also, you will be checked for adverse events, liver function tests, and kidney function abnormalities through hematological test, biochemical test and urinalysis. Samples (blood and urine) taken for these tests will be used in the hospital only for the above purposes and will not be used for any other purpose. They will be discarded immediately after completion of the tests without secondary use.

After 6 weeks after application, marshmallow esophagography will be performed to evaluate esophageal movement to verify the anti-adhesion efficacy.

And at each visit during the study, the investigator will ask you if you have changed the medication you were taking or if you have taken a new medication.

- Different procedures between the test group and the control group : The test group and the control group will participate in the clinical trial through the same procedure, and only the anti-adhesion product applied during surgery will be different.
- Marshmallow esophagography : Marshmallow esophagography is a method in which a subject takes a contrast medium coated marshmallow and measures the time the marshmallow passes through the esophagus depending on his/her posture.
- Expected benefits from the clinical trial : Subjects will be provided with MegaShield or Guardix-SG, an anti-adhesion product. They will also undergo a blood test, a urinalysis, a general chemical test, etc. Even when participating in this clinical trial, subjects may or may not obtain anti-adhesion effects. However, the information obtained from the study can help to compare and evaluate the anti-adhesion efficacy and safety of the anti-adhesion product after thyroidectomy.

3) Inclusion and exclusion

You can participate in this study only if you meet the following criteria:

(1) a patient who gives written informed consent spontaneously;
(2) a patient who is between 20 and 70 years of age;
(3) a patient who can participate the clinical trial during the entire study period;
(4) a patient who is required total thyroidectomy due to thyroid disease;
(5) a patient who are scheduled to undergo thyroidectomy for the first time related to thyroid disease;
(6) a patient with no liver dysfunction, anemia or renal inadequacy in preoperative testing; and
(7) a patient who agreed to contraception while participating in the clinical trial after application of investigational product.

You will be excluded from the study if you fall under any of the following criteria:

(1) a pregnant or breast-feeding female patient; or a female who plans to become pregnant within 1 month after applying the investigational product;
(2) a patient with serious liver or kidney disease;
(3) a patient with lymphatic or hemostatic disorders; or a patient taking an anticoagulant;
(4) a patient who receives an oral or a parenteral hypoglycemic agent for diabetes;
(5) a patient immunosuppressed; or a patient with autoimmune diseases;
(6) a patient with serious systemic disease;
(7) a patient scheduled for accompanied follow-up surgery;
(8) a patient undergoing chemotherapy for a cancer other than thyroid cancer;
(9) a patient who is treated by another anti-adhesion; or
(10) a patient deemed inappropriate for this study by the investigator (including non-cooperative, etc.).

4) Matters what you should follow if you sign the informed consent form

If you decide to participate in this clinical study, we will be provided with clinical and radiological findings, including the disease name, to screen your eligibility for this clinical study. Once you are enrolled in the clinical trial, you must comply with the protocol as directed by the investigator.

5) Risk or inconvenience of medical devices used in the clinical trial

There may be risks or adverse events due to the medical devices and surgery as shown below.

- Side effects associated with general surgery: Abscess, cellulitis, wound, dermal necrosis, swelling, hematoma, cardiovascular disease, hypertension, ischemia, thrombus, embolism, hemorrhage, complications related to anesthesia, pulmonary complications, nerve/muscle damages, etc.
- Side effects associated with the use of anti-adhesion product: Infection, allergic reaction, pain, and inflammatory reaction
- Side effects associated with total thyroidectomy: Hypoparathyroidism, recurrent laryngeal nerve injury, hemorrhage, infection, superior laryngeal nerve paralysis, dyspnea, etc.

Also, if you have marshmallow esophagography, you may have discomfort in swallowing. Esophagography is a common method used to check esophageal function, and in this clinical trial, X-rays will also be irradiated for a general diagnosis. So, if you are pregnant or may be pregnant, consult with the medical staff and decide whether to undergo the test or not. Also, the barium used during the test may cause you to suffer from nausea, vomiting, hives, dyspnea and constipation. Therefore, it is recommended that you drink enough water for 2 to 3 days after the test.

There may be side effects that are not known to date, and it is not yet known whether raw materials

used in the study will affect the fetus or lactating women. Therefore, pregnant and lactating women cannot participate in this study. And if you suspect you are pregnant during the study, you must notify your doctor immediately.

6) Cases and reasons for discontinuation of participation in clinical trial during the study

The investigator or sponsor may decide to exclude you from the clinical trial:

(1) Where the principal investigator determines that the situation observed during the clinical trial is unreasonable for continuing the clinical trial
(2) Where the clinical trial is to be discontinued due to the safety of the investigational product, etc.
(3) Where the study is temporarily discontinued to deal with an adverse event that has occurred
(4) Where the used medical device is removed and the study is discontinued to deal with an adverse event that has occurred
(5) Where the study is discontinued due to the occurrence of a serious adverse event/adverse device reaction

7) Matters concerning the compensation you will receive during your participation in clinical study

If you participate in this clinical trial, you will be provided with an anti-adhesion product for the clinical trial. The cost of tests related to the clinical trial (blood test, general chemical test, urinalysis, marshmallow esophagography, etc.) will be supported by research funds, and transportation costs of 50,000 won each visit (screening, 1 week after surgery, 6 weeks after surgery) will be paid to you (a total of 150,000 won). The transportation costs paid may be adjusted depending on the visit No. or duration of your participation in the study. The surgical fees for total thyroidectomy will not be supported.

8) Expected cost you (subject) will pay for participating in the clinical study

There is no cost to you to participate in this clinical trial.

9) Potential risks and benefits of other treatments you may choose from

The standard treatment you will receive is to use an anti-adhesion product on the market after total thyroidectomy. The commercial anti-adhesion products used include Guardix-SG and Hyalobarrier.

10) Compensation or treatment you will receive in the event of damages related to the clinical trial

If you participate in this clinical study and suffer damage from a medical device used in the clinical

study, you will be compensated according to the liability insurance policy and the 'Policy on Compensation for Victims.' In the event of an injury or adverse event related to the study, you should contact the investigator immediately.

11) Voluntary participation

There is no legal or ethical obligation for you to participate in this clinical study. Your participation in this study will have no effect on your care. Even if you decide to participate in the study, you can at any time express your intention to give up your participation halfway. Even if you give up your participation in the clinical study midway, there will be no disadvantage in continuing treatment at this hospital, and you will receive the same treatment with other patients without discrimination.

12) Direct access to your personal information

If you agree to participate in this clinical trial, monitors, reviewers, institutional review board and the Ministry of Food and Drug Safety can directly access your medical records to ensure that the clinical study has been properly conducted and to verify the reliability of the clinical study procedure and data within the scope of the relevant regulations without infringing on your confidentiality. If you or your legal representative sign the informed consent form, it means that you allow direct access to these materials.

13) Confidentiality of your personal information

Your name, address, telephone number, resident registration number, medical records, test results, and health information will be collected as a result of your participation in this study, but these information are only used for the purpose of connecting to clinical information obtained from the study and are properly controlled under the Privacy Act. If you withdraw your consent to participate in this study and request the disposal of data that is already being analyzed or has been analyzed, the data will also be discarded. Data will be collected on designated computers with restricted access in a locked storage area, and access to this data will be restricted to authorized investigators and protected from access by others. Study-related data will be stored for 3 years after the completion of the study and then discarded.

All records that can identify you will be kept confidential, and your identity will be kept confidential in publications related to the results of the clinical study.

14) Consideration for your safety

We will notify you or your representative immediately when we have any new information that may affect your willingness to continue participating in the study.

15) Inquiries about clinical study

If you would like to obtain additional information about this clinical study and your rights, or if you

have any other questions related to the clinical study, please contact us below.
Doctor in charge of clinical trial :
Department :
Telephone :
Coordinator :
Department :
Telephone
An original copy of the Subject Manual and a copy of the Informed Consent will be provided to the subject who participated in the study.
I, signed below, confirm that I have sufficiently explained the outline and purpose of this study to the patient or his/her legal representative.

Doctor in charge : (Signature)

Informed Consent

Title: Double-blind, Multi-center randomized, prospective confirmatory clinical study to evaluate the efficacy and safety of MegaShield using for anti-adhesion after total thyroidectomy

Please read the contents below and **tick the box** when you fully understand the contents.

☐ I have read this form, and I fully understand its contents.

☐ I, ______________ (***subject must write his/her full name***) received detailed explanations from the doctor in charge, asked questions, and received appropriate answers.

☐ I voluntarily participate in this study.

☐ I agree my health information to be used and shared as described in this informed consent form.

☐ I understand that I cannot participate in this clinical trial if I need treatment other than the clinical trial, if I do not follow this protocol, or if I am excluded from the inclusion criteria for subjects.

☐ I may refuse or discontinue participating in the clinical study at any time during the study. I also know that there is no disadvantage if I stop participating in this study.

☐ I request participation in the clinical trial with my free will and receive a copy of the consent form.

☐ I understand that I may be disqualified from the clinical trial due to inappropriate reasons for this study.

**Subject**	Name	______	Signature	______	Date	______
**Legal representative** (Relation:______)	Name	______	Signature	______	Date	______
**Observer**	Name	______	Signature	______	Date	______
**Investigator**	Name	______	Signature	______	Date	______

17. Policy on compensation for Subjects

This Policy on Compensation for Subjects is a compensation policy applicable to subjects participating in all clinical trials requested by L&C Bio Inc.

17.1 Reason for compensation for subject

L&C Bio Inc. will compensate the subject for physical damage caused by harmful and unintended reactions to the subject by the medical device used in the clinical trial conducted in accordance with Good Clinical Practice for Medical Devices. The damages will be compensated in accordance with the provisions of this Compensation Policy and the relevant laws in the following cases.

1) Temporary pain or damage to the extent that can be easily treated; where the institution determines that treatment is required (The range of compensation is limited to the necessary medical expenses.)
2) Where it is necessary to hospitalize or extend the hospitalization period
3) Where it causes persistent or significant disability or hypofunction
4) Where it causes birth defects or abnormalities
5) Where it causes death or is life-threatening

17.2 Compensation requirements

Subject compensation under this policy is made in accordance with the following requirements.
It should be physical damage caused by the investigational product for this clinical trial.
The investigator must have complied with the contents of the protocol approved by the Minister of Food and Drug Safety.
It must not be due to the investigator's apparent negligence or mistake.
Subject must have followed all instructions of the investigators.

The subject should take measures to minimize the occurrence of damage caused by the relevant physical damage.

17.3 Reasons for exclusion from compensation

Notwithstanding the provisions of the above 17.2, the following cases are excluded from the scope of compensation under this compensation policy.
1) Damage due to insufficient efficacy and effect expected from the investigational product (including the cases caused by progression or deterioration of past history of the subject)
2) Damage caused by the subject's carelessness

17.4 Standard of compensation

1) If there is a compensation amount or action agreed in advance between the parties for expected adverse device reactions, it will be compensated according to the standard.
2) Other cases will be compensated in accordance with the compensation method agreed between the parties in comprehensive consideration of the degree, nature, duration and similar reactions of the physical damage.
3) If there is no agreement between the parties, it will be compensated according to the determination of the court and the equivalent decision.

17.5 Compensation procedure

1) Subjects who have suffered physical damage should request necessary medical treatment from the investigator or the institution of this clinical trial in accordance with this compensation policy.
2) Subjects whose physical damage has not been completely cured despite the principal investigator's or institution's action may request compensation from the sponsor.
3) After receiving the above request for compensation, the sponsor will immediately investigate whether the subject is eligible for compensation and the criteria for compensation and notify the subject of this.
4) If the subject has any objection to the contents of the notification above, he/she must notify the sponsor of the objection within [5] working days from the date of receiving the notification.
5) If the subject does not notify the objection to this after receiving the notification under the above 3), it is understood that both parties have agreed to compensation in accordance with the above notification.
6) If the subject notifies the objection pursuant to the above 4), the sponsor shall recommend to the subject several objective experts to determine whether the subject is eligible for compensation and the standard, and the subject shall designate one of them within [3] working days from the

recommendation date. (If the subject does not designate, the sponsor will choose randomly.)

17.6 Application Scope

1) This compensation policy is generally applied within the scope of all clinical trials requested by the sponsor unless there is another agreement between the sponsor and the subjects.
Any agreement made by the subject with other third parties related to the clinical trial without the sponsor's approval for compensation has no effect on the sponsor.
We hereby pledge to ensure that the subject is not subject to any disadvantage by this clinical trial based on the above contents and to take responsibility in accordance with the Compensation Policy for Victims if any problems arise from this clinical trial.

18 Matters concerning the treatment of subjects after clinical trial

After the completion of this clinical trial, the subject shall follow the treatment procedures of the hospital with respect to subsequent care, and the subsequent treatment fees shall be paid by the subject. However, if a side effect occurs, it is necessary to check whether there is a causal relationship with the medical devices used for the clinical trial. If any side effect occurs due to the medical devices used in this clinical trial, L&C Bio Inc. will pay for the treatment until the side effect disappears.

19 Measures for the safety protection of subjects

19.1 Institution

The head of the institution should have the clinical laboratory, facilities and experts necessary to conduct the clinical trial, and should ensure that the clinical trial can be properly carried out, such as taking necessary measures in case of an emergency.

19.2 Institutional review board

- The Institutional Review Board (IRB) should be organized in accordance with domestic laws and regulations. The IRB should protect the rights, safety and well-being of subjects, and if vulnerable subjects participate in the clinical trial, the validity of the reasons should be closely reviewed.
- The IRB should take necessary measures, such as ordering the discontinuation of part or all of the clinical trial to the principal investigator, if a subject do not properly agree to participate in the study, if the clinical trial was not conducted according to the protocol, or if a serious adverse

event/adverse device reaction occurs.

19.3 Investigator

- Investigator refers to the principal investigator, the sub-investigator, and the coordinator. The investigator should conduct the clinical trial in compliance with the protocol agreed with the sponsor and approved by the IRB and the Minister of Food and Drug Safety.
- During or after the clinical trial, the investigator should take measures to ensure that subjects receive appropriate medical treatment for all adverse events occurred in the clinical trial, including clinically significant laboratory test abnormalities. In addition, the investigator should inform subjects if medical treatment is required for their intercurrent disease.
- The investigator should accurately analyze and understand the protocol and actively respond to the subjects' problems.

19.4 Sponsor

- Sponsor is a person who is responsible for the planning, management, finance, etc. of a clinical trial, and usually refers to a medical device manufacturer (including importer) for a clinical trial for a medical device.
- The sponsor should ensure that the subject, procedure, Case Report Form and the contents are carried out in accordance with the procedures specified in the protocol.
- The audit plans and procedures performed by the sponsor should be determined depending on the importance of the clinical trial, the number of subjects, the type and complexity of the clinical trial, the degree of potential risks to subjects, and problems with the clinical trial that have already been identified.

19.5 Monitoring

- Monitoring refers to activities that supervise the progress of the clinical trial and examine and review that the clinical trial is conducted and recorded in accordance with the protocol, the standard operating procedure, the Good Clinical Practice, and related regulations.
- Monitoring of the clinical trial will be performed through regular visits to the institution and phone calls by the clinical trial monitor personnel. When visiting, the monitor shall check the original patient record, the management record of the investigational products, and data storage (research file), etc.
- And the monitor examines the progress of the clinical trial and consults with the investigator if there is a problem.

19.6 Revision of protocol

- After approval of the protocol from the Institutional Review Board and the Minister of Food and Drug Safety, in the case of the protocol is altered due to becoming widespread testing procedures, rising risk, changing in the criteria for selection of subjects, or additional safety information, it should be approved by the Institutional Review Board and the Minister of Food and Drug Safety.
- When revising the protocol, the date, reason and details of revision should be recorded and stored.
- The investigator should not perform a clinical trial differently from the protocol before approval of the change by the IRB and the Minister of Food and Drug Safety, unless it is necessary to remove the immediate risk factors that have occurred in the subject. If such a change in the protocol is applied prior to obtaining IRB approval to eliminate immediate risk factors incurred in the subject, the change should be submitted to the IRB (for approval of post-review), the sponsor, and the Minister of Food and Drug Safety as soon as possible.

In addition, documents approved by the chairperson or secretary of the IRB should be sent to the sponsor.
- Minor modifications or specifications that do not affect clinical trials do not necessarily require approval, but administrative changes are necessary.

19.7 Informed Consent

- Informed Consent refers to a procedure that the subject is provided with all information related to the clinical trial through a description for the subject before deciding whether or not to participate in the clinical trial, and confirm that the subject voluntarily participates in the clinical trial through a document containing the signature and the date of signature.
- If the subject or his/her substitute cannot read the form of Informed Consent, Information Sheet for the Subject, and other documented information, an impartial observer should attend the entire process of obtaining consent.
- Before obtaining consent, the investigator should give sufficient time and opportunity for the subject or his/her representative to inquire about the details of the clinical trial and decide whether to participate in the clinical trial, and also should answer all questions related to clinical trial to the satisfaction of the subject or his/her representative.

19.8 Confidentiality of subject records

- Records that can identify the subject's identity should be kept confidential, and it should be kept confidential even when the results of clinical trials are published.

- The sponsor, monitors and reviewers related to this clinical trial may view the subject's records for the purpose of monitoring, reviewing and managing the progress of this clinical trial. Through signing this protocol, the investigator acknowledges that the sponsor or monitors and reviewer of the clinical trial may review or copy the relevant documents to verify the records of the subject's charts and case records in terms of domestic laws and ethics. Such information should be kept confidential.
- All documents related to clinical trials, such as Case Report Form, should be recorded and classified with the subject identification code, not the subject's name.

19.9 Record keeping

Various data and records related to the conduct of clinical trials should be kept well and security should be maintained. After the completion of the clinical trial result report, documents related to the clinical trial should be preserved for 3 years from the date of the clinical trial termination.

20 Other matters necessary for the safe and scientific conduct of clinical trial

20.1 Use and management of investigational products

- The investigational product should be managed by the medical device manager designated in "Name and position of managers who manage investigational products" in Section 4 of this protocol.
- Investigational products should be handled and stored as described in the stated matter, and the phrase "for clinical trial" should be marked. The manager of the investigational product should perform tasks such as acquisition, inventory management, and return of medical devices used in the clinical trial, and also should maintain related records.

20.2 Supply and handling of investigational products

- The sponsor should not supply the investigational products to managers, etc. before obtaining approval from the Institutional Review Board and the Minister of Food and Drug Safety for the protocol.
- The sponsor should have a documented procedure on how the manager, etc. handles and stores the investigational products, and this procedure should include methods for appropriate and safe acquisition, handling, storage, and return of unused investigational products from the subject and return to the sponsor.
- The investigational products should be supplied in a timely manner, and records of supply to the

institutions, acquisition of the institutions, and return from the institutions and disposal should be maintained.

- The sponsor should establish and document a collection system for the investigational products when a problem such as a failure occurs in the investigational product, or due to the closure of the clinical trial or expiration of the period of use, etc.

21 References

1. Ellis, H., et al., *Adhesion-related hospital readmissions after abdominal and pelvic surgery: a retrospective cohort study.* The Lancet, 1999. **353**(9163): p. 1476-1480.
2. Lower, A.M., et al., *The impact of adhesions on hospital readmissions over ten years after 8849 open gynaecological operations: an assessment from the Surgical and Clinical Adhesions Research Study.* BJOG: An International Journal of Obstetrics & Gynaecology, 2000. **107**(7): p. 855-862.
3. Weibel, M.-A. and G. Majno, *Peritoneal adhesions and their relation to abdominal surgery: a postmortem study.* The American Journal of Surgery, 1973. **126**(3): p. 345-353.
4. Rosato, L., et al., *Recurrent laryngeal nerve damage and phonetic modifications after total thyroidectomy: surgical malpractice only or predictable sequence?* World journal of surgery, 2005. **29**(6): p. 780-784.
5. Menzies, D. and H. Ellis, *Intestinal obstruction from adhesions--how big is the problem?* Annals of the Royal College of Surgeons of England, 1990. **72**(1): p. 60.
6. Ray, N.F., et al., *Abdominal adhesiolysis: inpatient care and expenditures in the United States in 1994.* Journal of the American College of Surgeons, 1998. **186**(1): p. 1-9.
7. Van Der Krabben, A., et al., *Morbidity and mortality of inadvertent enterotomy during adhesiotomy.* British Journal of surgery, 2000. **87**(4): p. 467-471.
8. Oh, A., *Trends of Anti-adhesion Adjuvant-Review.* Biomater. Res, 2013. **17**: p. 138-145.
9. Burns, J.W., et al., *Preclinical evaluation of Seprafilm bioresorbable membrane.* The European journal of surgery. Supplement.:= Acta chirurgica. Supplement, 1997(577): p. 40-48.
10. Falk, K., et al., *Reduction of experimental adhesion formation by inhibition of plasminogen activator inhibitor type 1.* British journal of surgery, 2001. **88**(2): p. 286-289.
11. Müller, S.A., et al., *A hydrogel for adhesion prevention: characterization and efficacy study in a rabbit uterus model.* European Journal of Obstetrics & Gynecology and Reproductive Biology, 2011. **158**(1): p. 67-71.
12. Shim, H.S., et al., *Evaluation of resorbable materials for preventing surgical adhesion on rat experiment.* Journal of the Korean Surgical Society, 2002. **63**(3): p. 179-186.
13. Oh SH, Kim JK, Song KS, Noh SM, Ghil SH, Yuk SH, et al. *Prevention of postsurgical tissue adhesion by anti-inflammatory drug-loaded pluronic mixture with sol-gel transition*

behavior. J Biomed Master Res 2005;72:306-316.

14. Oh, S.H., et al., *Prevention of postsurgical tissue adhesion by anti-inflammatory drug-loaded pluronic mixtures with sol–gel transition behavior.* Journal of Biomedical Materials Research Part A, 2005. **72**(3): p. 306-316.
15. Kang, S.-W., et al., *Preventive Effect of Human Acellular Dermal Matrix on Post-thyroidectomy Scars and Adhesions: A Randomized, Double-Blinded, Controlled Trial.* Dermatologic Surgery, 2015. **41**(7): p. 812-820.
16. Yi KH, Lee EK, Kang HC, et al., *2016 Revised Korean Thyroid Association Management Guidelines for Patients with Thyroid Nodules and Thyroid Cancer.* Int J Thyroidol 2016 November 9(2): 59-126
17. Chow, S.C.; Shao, J.; *On non-inferiority margin and statistical tests in active control trials,* Statist. Med. 2006; 25:1101–1113
18. Grindel JM, Jaworski T, Emanuele RM, Culbreth P. *Pharmacokinetics of a novel surface-active agent, purified poloxamer 188, in rat, rabbit, dog and man.* Biopharm Drug Dispos 2002;23:87-103.
19. Li C, Palmer WK, Johnston TP. *Disposition of poloxamer 407 in rats following a single intraperitoneal injection assessed using a simplified colorimetric assay.* J Pharm Biomed Anal 1996;14:659-665.
20. Dumortier G, Grossiord, Jean., Agnely, Florence., and Chaumeil, Jean. *A Review of Poloxamer 407 Pharmaceuticaland Pharmacological Characteristics.* Pharm Res. 2006;23(12):2709-2728. 23
21. Karthe Ponnuraj and Mark J. Jedrzejas. *Mechanism of Hyaluronan Binding and Degradation: Structure of Streptococcus pneumoniae Hyaluronate Lyase in Complex with Hyaluronic Acid Disaccharide at 1.7 AÊ Resolution.* J. Mol. Biol 2000, 299, 885-895
22. J. Necas, L. Bartosikova, P. Brauner, J. Kolar. *Hyaluronic acid (hyaluronan): a review.* Veterinarni Medicina, 2008;53(8): 397–411
23. Kataoka K, Suzuki Y, Kitada M, Hashimoto T, Chou H, Bai H, et al. *Alginate, a bioresorbable material derived from brown seaweed, enhances elongation of amputated axons of spinal cord in infant rats.* J Biomed Master Res 2001;54:373-384.
24. Park J.H., et al., *The Efficacy and Safety of Guardix-SG® in Patients Who Are Undergoing Thyroid Surgery: A Randomized, Prospective, Double-blinded Study.* Korean J Endocrine Surg 2009;9: 127-132
25. Seo J.K, et al., The significance of the Esophagogram with a *Marshmallow Bolus:* The Korean Journal of Gastroenterology, 1996. **28**(3): p. 303-310.
26. Song J.W. et al., Clinical value of Marshmallow Esophagography in Detecting Esophageal Dysmotality. The Korean Journal of Gastroenterology, 2000. **35**(4): p. 405-412.
27. Park W.S., et al., *Anti-adhesive effect and safety of sodium hyaluronate and sodium*

carboxymethyl cellulose solution in thyroid surgery. Asian Journal of Surgery, 2010. **33**(1): p. 25-30
