# Supplementary material for: Efficacy and safety of temperature-sensitive acellular dermal matrix in prevention of postoperative adhesion after thyroidectomy: A randomized, multicenter, double-blind, non-inferiority study
Source: PLoS One. 2022 Sep 19;17(9):e0273215. doi: 10.1371/journal.pone.0273215 (PMC9484646; doi:10.1371/journal.pone.0273215)
Supplement: Attachment — Submitted filename: Response to reviewers.docx [file pone.0273215.s006.docx]

November 4, 2021

Emily Chenette

Editor-in-Chief

*PLOS ONE*

Dear Editor:

Thank you for giving us the opportunity to submit a revised draft of our manuscript titled “Efficacy and Safety of Temperature-sensitive Acellular Dermal Matrix in Prevention of Postoperative Adhesion after Thyroidectomy: A Randomized, Multicenter, Double-blind, Non-inferiority study”

to *PLOS ONE*. We appreciate the time and effort that you and the reviewers have dedicated to providing valuable feedback regarding our manuscript. We are grateful to the reviewers for their insightful comments on our paper. We have been able to incorporate changes to reflect most of the suggestions provided by the reviewers. The changes made within the manuscript have been highlighted to aid navigation.

Here are our point-by-point responses to the reviewers’ comments and concerns.

Comments from Reviewer 1

- **Comment 1**: *As the study aims to prove a non-inferiority hypothesis the appropriate CONSORT Checklist should be applied and the structure of the paper should follow the section/topics of the guideline. That helps by identification of the important information of this study type. Among other, the study type as non-inferiority study should be included in the title. Please note also that ervey point in the guideline should be addressed, e.g. 3b should be commented as: No changes to methods after trial commencement are implemented. In particular this information is important as the trial register does not allow to follow the history of changes. See: Piaggio G (2012), PMID: 23268518.*

**Response:** Thank you for your valuable comment. In this revision, we did our best to comply with the CONSORT guidelines. We have also commented, “No changes to methods after trial commencement are implemented” in the methods section (page 4, paragraph 3, line 100).

- **Comment 2**

*There is no appended individual patient level data file, so that reproducibility is limited.*

**Response:** It is not clear as to what the individual patient level data imply, as the PLOS ONE guidelines do not specifically obligate it. However, we have provided the score frequency in Table 4, which made it easier to analyze the primary outcome and arrive at the same conclusion.

- **Comment 3**

*As most of the details, e.g. the non-inferiorty margin including justification, these information is included only on the side so that study objective is unclear most the time. This would be solved, if the CONSORT recommendation is followed strictly*

Response: Thank you for your comment which made the structure of this manuscript more systematic. As we have commented above, we have tried to follow the CONSORT recommendation strictly in the revised manuscript.

- **Comment 4**

*L83ff: Include the hypothesis and endpoint in the definition of the aim*.

Response: Thank you for your comment. Accordingly, we have described the hypothesis and outcomes (page 4, paragraph 2, line 86–88).

- **Comment 5**

*L105: Block size is missing in the description of PBR.*

**Response:** Thank you for your comment. Accordingly, we have added the information on the block size. (page 5, paragraph 2, line 111).

- **Comment 6**

*L105f: It is unclear whether the randomization is stratified by center.*

**Response:** According to your comment, we have clearly stated that the randomization is stratified by center. (page 5, paragraph 2, line 111).

- **Comment 7**

*L109: It is unclear how blinding is implemented. Please give details. I assume that treatments could not be blinded. Give details for blinding of clinical assessors.*

**Response:** Thank you for your valuable comment. We agree that the implementation of blinding can be explained more clearly based on the study protocol. We have added this information in the manuscript. (page 5, paragraph 2, line 114–121).

- **Comment 8**

*L132: As biostatistican it is not clear to me, whether the primary outcome in the manuskrip “extent of adhesion as assessed using Marshmallow esophagography” equals “esophageal movement using Marshmallow” or “” which is given in the protocol.*

**Response:** Thank you for your insightful comment. We agree that the similarity of the expressions should be resolved. We think that the “extent of adhesion as assessed using marshmallow esophagography” is what we wanted to evaluate with “esophageal movement using marshmallow”. We have changed the expression in the manuscript to “esophageal movement using marshmallow”, following the expression in the guideline. To make this more logical, we have explained it in the discussion section. (page 17, paragraph 2, line 298–304).

- **Comment 9**

*L161: Statistical tests on baseline characterisics is not. Useful in randomized trials. Delete column 4. Give numbers recruited patients in groups Per “center”.*

**Response:** Thank you for your suggestion. As per your comment, we have modified Table 1.

- **Comment 10**

*L172f: Give more details for sample size calculation. I guess your argument is based on the z-statistic as in the protocol. Describe your arguments for the non-inferiority margin. Give arguments for the dropout rate, e.g. by referring to the literature. The ICH E9 stated to do the ITT and PP and they should yield the same results. SO you may proceed similarly by giving the two one sided 95% Cis. Actually, the method does not result in a conservative test decision and the method of Sanchez (Statistics in Medicine, 2006) may be a useful as an alternative. You just have to argue.*

Response: Thank you for your suggestion. I agree that a detailed explanation of the protocol should be presented in the manuscript. We have described it in the manuscript with the arguments for non-inferiority margin (page 8, paragraph 1, lines 168–174). Regarding the ICH E9, we have commented that we obtained the same results with both ITT and PP groups with the reference that you had recommended (page 17, paragraph 3, lines 305–307).

- **Comment 11**

*L183: I assume you mention statistical tests on the secondary endpoint etc. There are good arguments to avoid pretest for normality – so maybe you delete that text. Second, the tests could not be associated to the parameters, so it is best to mention the test in the result section at places where it is used. Give the software used for statistical computation*

**Response:** I appreciate your professional comment. Based on your comment, we have deleted the relevant sentence. We included the details of the software used for statistical computation (SAS ver. 9.4; SAS Institute, NC, USA) at the beginning of the paragraph (page 8, paragraph 1, line 183-184). Further, if possible, we would like to add footnotes to the tables pertaining to the correlated statistical test to add clarity to the manuscript.

- **Comment 12**

*L187f: Describe the setting first. Report on enrollment time, patients per center, and in particular deviations in the randomization process*.

**Response:** I appreciate your comment. We have described the setting including enrollment time and patients per center. We have also commented that no deviations occurred during the randomization process. (page 8, paragraph 2, lines 188–189 and page 9, paragraph 1, lines 191–193).

- **Comment 13**

*L193: I see the exclusion of 11 patients critical with regard to the ITT principle. As there are longitudinal measurements other strategies to handle missing observations may be more appropriate. In particular with regard to figure 1, where only 1 patient is withdrawn in each group.*

**Response:** We admit that Figure 1 may be appear confusing to the readers. Therefore, we have revised it to explain the whole patient setting. Further, the data of the patients who were included in ITT but excluded in mITT were not formally approved at the time of the first postoperative assessment (visit 3). Alternatively, there were no missing observations to substitute in mITT group.

- **Comment 14**

*L209f: Use the analysis population mentioned above. Skip the p-values. Give p-values with at least three decimal digits!*

**Response:** As we have commented previously, it seems that the analysis of mITT group does not contain statistical problems. We have described this in the discussion section, which also corresponds to the answer to your comment 10.

- **Comment 15**

*L226: Please improve the layout of the table 5, so that no line break occurs in numbers. As the data are compared by Wilcoxon test, the appropriate statistic based on provision Probability should be stated (WMWodds, see Divine, 2018). On the other hand, the longitudinal measurements may be analysed by Mixed effects models, rather than splitting over time.*

**Response:** We thoroughly read the article written by Divine et al. Indeed, the Wilcoxon test has a limitation as a test of medians. However, this study aimed to evaluate the difference between two groups rather than perform a median test. As we had planned to use the Wilcoxon-Mann-Whitney test for statistical analysis in this study, based on its widespread usage for analysis of non-normally distributed data, we would like to retain the statistical results. We have described this as a limitation in the discussion section (page 18, paragraph 4, lines 334– page 19, paragraph 1, line 337).

- **Comment 16**

*L288: Limitation should address and discuss potential sources of bias in the trial and success of implemented measures against bias*

**Response:** We agree that we must add a comment pertaining to the potential source of bias in this study. However, as evident from the study guideline (11.3.1), we attempted to minimize the statistical biases with double-blinding and randomization generated using SAS by a statistical expert who was not directly related to the institution. For blinding, before the study was terminated, only a minimum number of personnel who are not directly related to the institution were able to access the randomization table and code. Regarding financial bias, this study was stated to be sponsored by L&C bio in the protocol; however, the study grant was finally sponsored by the Ministry of Health & Welfare as mentioned in the financial disclosure.

- **Comment 17**

*L299: This is rather a result than a clinical conclusion. What does the result mean for practice?*

*‘***Response:** We appreciate your comment. We have added a sentence to the conclusion to show the clinical usefulness of this study (page 19, paragraph 2, line 342–344).

Comments from Reviewer 2

- **Comment 1**

*Well designed study.*
*I would have liked a more detailed explanation of the marshmallow oesophagography technique. The transit time is used as a surrogate marker for adhesions. I would have liked to see some evidence or justification that an increased transit time correlates with the degree of adhesions.
The subjective scoring system to assess adhesions could be improved.*

**Response:** We appreciate your comment. We agree that the association between the extent of adhesion and the transit time on marshmallow esophagography can be explained more thoroughly. Based on your comment, we have added more references and relevant explanations. (page 17, paragraph 2, lines 298–304).

In addition to the above comments, all spelling and grammatical errors have been corrected.

We look forward to hearing from you in due course regarding our submission and will be glad to respond to any further questions and comments that you may have.

Kee-Hyun Nam

Address: Department of Surgery, Yonsei University College of Medicine,

50-1, Yonsei-ro, Seodaemun-gu, Seoul, Republic of Korea

Phone: +82-2-2228-2100

Email: khnam@yuhs.ac
