# Supplementary material for: Efficacy and safety of temperature-sensitive acellular dermal matrix in prevention of postoperative adhesion after thyroidectomy: A randomized, multicenter, double-blind, non-inferiority study
Source: PLoS One. 2022 Sep 19;17(9):e0273215. doi: 10.1371/journal.pone.0273215 (PMC9484646; doi:10.1371/journal.pone.0273215)
Supplement: Attachment — Submitted filename: Responst to Reviewers.docx [file pone.0273215.s007.docx]

January 25, 2022

Emily Chenette

Editor-in-Chief

*PLOS ONE*

Dear Editor:

We wish to once again thank you for your thoughtful review and consideration of our manuscript, entitled “**Efficacy and Safety of Temperature-sensitive Acellular Dermal Matrix in Prevention of Postoperative Adhesion after Thyroidectomy: A Randomized, Multicenter, Double-blind, Non-inferiority study**,” for publication in *PLOS ONE*.

We appreciate the valuable feedback your team has provided during each stage of the submission process, as well as the time and effort required to do so. We have taken care to revise the text in accordance with the comments and suggestions provided and address any remaining spelling/grammatical errors. To aid in the review process, we have highlighted these changes in the revised manuscript, and our point-by-point responses to the editors and reviewers’ concerns are listed below.

We believe that these revisions have substantially improved our paper, which we hope is now suitable for publication in your esteemed journal. We look forward to hearing from you in due course regarding our submission and will be glad to respond to any further questions and comments that you may have.

Sincerely,

Kee-Hyun Nam

Address: Department of Surgery, Yonsei University College of Medicine,

50-1, Yonsei-ro, Seodaemun-gu, Seoul, Republic of Korea

Phone: +82-2-2228-2100

Email: khnam@yuhs.ac

Journal Requirements

*Please review your reference list to ensure that it is complete and correct. If you have cited papers that have been retracted, please include the rationale for doing so in the manuscript text, or remove these references and replace them with relevant current references. Any changes to the reference list should be mentioned in the rebuttal letter that accompanies your revised manuscript. If you need to cite a retracted article, indicate the article’s retracted status in the References list and also include a citation and full reference for the retraction notice.*

**Response:** As per your comment, we have changed or deleted several references. The four references listed below were not retracted articles, but were published in Korean journals, which means that they were not internationally acceptable. We apologize for our disregard for proper referencing.

Reference 3 (Oh et al. Trends of anti-adhesion adjuvant-review) was deleted. The journal (biomaterials research) was originally a Korean journal and became an international journal after 2014. Unfortunately, the reference we had used was from 2013. We found that deleting this reference made no difference in the content, so we decided to delete this article from the reference list.

Reference 12 (Lee et al. The efficacy and safety of HA/CMC anti-adhesion barrier solution with varying viscosities) was changed to reference 11, which is a reference from an international journal (Leach et al, Reduction of postsurgical adhesion formation in the rabbit uterine horn model with use of hyaluronate/carboxymethylcellulose gel. Fertility and sterility. 1998;69: 415-418).

Reference 26 and 27 (Song et al. Clinical value of marshmallow esophagography in detecting esophageal dysmotility and Park et al. Significance of functional esophagogram with a marshmallow bolus) were changed to reference 25, which is an internationally acceptable reference (Ott et al. Evaluation of the esophagus with a marshmallow bolus: Clarifying the cause of dysphagia. Gastrointestinal radiology. 1991;16: 1-4

Comments from Reviewer 1

**Comment 1**: *Thank you for considering my comments. There is only one point to be addressed in the final review. The presentation of the results of the Wilcoxon test needs providing the appropriate statistic based on provision Probability, i.e. WMWodds (see Divine, 2018).*

**Response:** We thank the reviewer for this valuable comment regarding the appropriate reference for the latest analysis. Accordingly, the results are now presented in Table 5 (page 13). We used the SAS program provided in *Divine et al., (2018)*. We hope that this revision is in accordance with the statistical standards of the journal.
